# Generalized Seasonal Autoregressive Integrated Moving Average Models for Count Data with Application to Malaria Time Series with Low Case Numbers

**DOI:** 10.1371/journal.pone.0065761

**Published:** 2013-06-13

**Authors:** Olivier J. T. Briët, Priyanie H. Amerasinghe, Penelope Vounatsou

**Affiliations:** 1 International Water Management Institute, Colombo, Sri Lanka; 2 Department of Epidemiology and Public Health, Swiss Tropical and Public Health Institute, Basel, Switzerland; 3 University of Basel, Basel, Switzerland; 4 International Water Management Institute Sub Regional Office for South Asia, Patancheru, Andhra Pradesh, India; Johns Hopkins University, United States of America

## Abstract

**Introduction:**

With the renewed drive towards malaria elimination, there is a need for improved surveillance tools. While time series analysis is an important tool for surveillance, prediction and for measuring interventions’ impact, approximations by commonly used Gaussian methods are prone to inaccuracies when case counts are low. Therefore, statistical methods appropriate for count data are required, especially during “consolidation” and “pre-elimination” phases.

**Methods:**

Generalized autoregressive moving average (GARMA) models were extended to generalized seasonal autoregressive integrated moving average (GSARIMA) models for parsimonious observation-driven modelling of non Gaussian, non stationary and/or seasonal time series of count data. The models were applied to monthly malaria case time series in a district in Sri Lanka, where malaria has decreased dramatically in recent years.

**Results:**

The malaria series showed long-term changes in the mean, unstable variance and seasonality. After fitting negative-binomial Bayesian models, both a GSARIMA and a GARIMA deterministic seasonality model were selected based on different criteria. Posterior predictive distributions indicated that negative-binomial models provided better predictions than Gaussian models, especially when counts were low. The G(S)ARIMA models were able to capture the autocorrelation in the series.

**Conclusions:**

G(S)ARIMA models may be particularly useful in the drive towards malaria elimination, since episode count series are often seasonal and non-stationary, especially when control is increased. Although building and fitting GSARIMA models is laborious, they may provide more realistic prediction distributions than do Gaussian methods and may be more suitable when counts are low.

## Introduction

There is increasing interest in using malaria prediction models to help clinical and public health services strategically implement prevention and control measures [1]–[5]. The Anti Malaria Campaign Directorate of the Ministry of Health in Sri Lanka has tested a malaria forecasting system that uses multiplicative seasonal autoregressive integrated moving average (SARIMA) models, which assume that logarithmically transformed monthly malaria case count data are approximately Gaussian distributed. Such an approach is widely used in predictive modelling of infectious diseases [4], [6], [7]. Malaria in Sri Lanka is seasonal and unstable and fluctuates in intensity, both spatially and temporally [8]. Malaria was a major public health problem in the country [9] until incidence started to dwindle in 2000 [10]. Sri Lanka entered the pre-elimination phase in 2007 and progressed to the elimination phase in 2011 [11].

Box-Cox class transformation of malaria counts (such as a logarithmic transformation) may yield approximately Gaussian distributed data, however, approximation is less close for observations with a low expected mean [12]. Also, low count data may include zeros, which renders Box-Cox transformation inapplicable. To overcome this problem, a small constant can be added to the data. Gaussian modelling with transformed data may result in inaccurate prediction distributions. This is problematic, particularly when the most recent monthly case counts are low, which tends to be the case in countries in the advanced phase of elimination [3]. Models that assume a negative binomial distribution for malaria count data may be more appropriate [13]–[15]. However, negative binomial models that incorporate a SARIMA structure are not yet available.

Benjamin and colleagues [16] provide a framework for generalized linear autoregressive moving average (GARMA) models, and discuss, 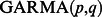 models for Poisson and negative binomially distributed data, among others. GARMA models are observation-driven models that allow for lagged dependence in observations. Alternatively, parameter-driven models (also) allow dependence in latent variables [17]–[20]. GARMA models are easier to estimate and prediction is straightforward, while parameter-driven models are easier to interpret [21], [22]. Jung and colleagues [23] find that both types of models perform similarly.

GARMA models relate predictors and ARMA components to a transformation of the mean parameter of the data distribution (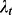), via a link function. A log link function ensures that 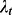 is constrained to the domain of positive real numbers. Lagged observations used as covariates should, therefore, also be logarithmically transformed, which is not possible for observations with a value of zero. To circumvent this problem, Zeger and Qaqish [24] discuss adding a small constant to the data, either to all data or only to zeros. Grunwald and colleagues [25] consider a conditional linear autoregressive (CLAR) model with an identity link function. In order to ensure a positive 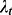, restrictions can be put on the parameters. A variant of the GARMA model, a generalized linear autoregressive moving average (GLARMA) model, is presented by Davis and colleagues [22].

Heinen [26] proposes a class of autoregressive conditional Poisson (ACP) models with methods that allow for over and under dispersion in the marginal distribution of the data. Another class of Poisson models with auto correlated error structure uses “binomial thinning”, and are called integer-valued autoregressive (INAR) models [27]. INAR models may be theoretically extended to moving average (INMA) and INARMA models [28], [29], but these are not easily implemented [30].

An alternative parameter-driven modelling approach assumes an autoregressive process on time specific random effects introduced in the mean structure, using a logarithmic link function [31]. Such a model is sometimes called a stochastic autoregressive mean (SAM) model [23] and has frequently been applied in Bayesian temporal and spatio-temporal modelling [15], [21], [32]–[36].

Of the models discussed above, the GARMA framework appears to be the most flexible for modelling count data with an autoregressive and/or moving average structure. Benjamin and colleagues [16] apply a stationary GARMA model to a time series of polio cases with a seasonal trend, using a sine/cosine function with a mixture of an annual and a semi-annual cycle. However, if the seasonal component is assumed to be stochastic, the GARMA model presented by Benjamin and colleagues [16] is not appropriate. Also, many time series of count data, including malaria cases, are non stationary.

Here, GARMA was extended to a class of generalized multiplicative seasonal autoregressive integrated moving average (GSARIMA) models, analogous to SARIMA models for Gaussian distributed data. The class of GSARIMA models includes generalized autoregressive integrated moving average (GARIMA) models. Model fit was carried out using full Bayesian inference. The effect of incorrect distributional assumptions on the posterior predictive distributions was demonstrated using simulated and real malaria case count data from Sri Lanka. Software code is provided as supporting information.

## Methods

### Model Formulation

Let 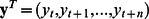 be a time series of count data of length *n* arising from a negative binomial distribution 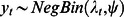 with 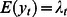 and 
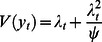
. The limiting form of the negative binomial distribution, that is 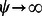, is the Poisson distribution.

The 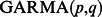 model can be written:
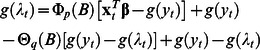
where 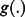 is a link function, 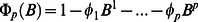, and 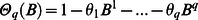. 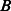 is a backshift operator with 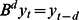 (note that 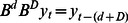). 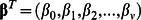 is a vector of coefficients for 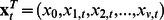 which includes an intercept multiplier (usually taken as 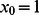) and 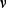 time dependent covariates. In the GARMA framework, count data could be modelled via a logarithmic or an identity link function, whichever is most appropriate for the series. To avoid the problem of taking the logarithm of observations with value zero under the logarithmic link, Zeger and Qaqish [24] propose a transformation 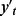 of 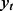 such as 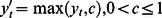, henceforth called “ZQ1”. Zeger and Qaqish [24] also suggest an alternative method, henceforth called “ZQ2”, which translates into the model variant:



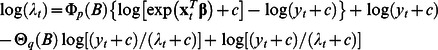



Under an identity link, restrictions may be necessary to ensure a positive 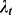, depending on the data and model parameters.

The above models can be extended to 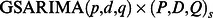 analogues by including seasonality (S) and differencing (I) components as follows: 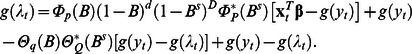
where 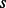 is the length of the period (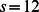 for monthly data with an annual cycle), 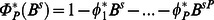, 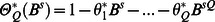, 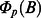, 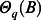, and 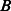 are as above. Examples of negative binomial 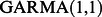 and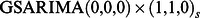 models with log link function and ZQ1 transformation are given in Appendix S1. The influence of link function choice and data transformations choices on the distribution of data are also assessed in Appendix S1.

### Model Fit

Benjamin and colleagues [16] employ maximum likelihood estimation through iterative weighted least squares and base inference on asymptotic results. In this paper, the model was formulated in a Bayesian framework.

In Bayesian inference, prior distributions need to be assigned to all model parameters. A weakly stationary model was assumed and, therefore, the auto correlation and moving average parameters were constrained using an algorithm provided by Jones [37]. For this purpose, the autoregressive and moving average parameters in the likelihood were reparameterized and prior distributions were adopted on the new parameterization. For example, the non seasonal autoregressive parameters 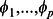 were reparameterized in terms of 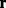, 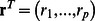, where 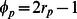 and 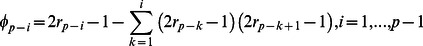. The following prior distributions were assumed: 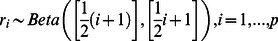, where 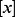 denotes the integer part of 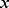. Further priors chosen were 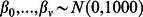 and 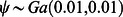.

For the first 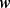 observations, the residuals on the predictor scale (*e.g.* 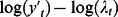 in the case of a logarithmic link function) were set to zero. A restriction can be put on the mean 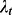 itself, that is 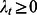 when the identity link is used. The GSARIMA models were estimated using the free Bayesian software programme, “JAGS” [38], which employs Markov chain Monte Carlo (MCMC) simulation methods. Examples of code written for using JAGS within the R software, for negative binomial GSARIMA models with logarithmic link function and ZQ1 transformation, are provided as supporting information [see Additional file S1].

The ability of these models to estimate simulated data series with GSARIMA structure is briefly explored in Appendix S1. The effect of (mis)specifying the link function and data transformation when estimating GARMA model parameters is also assessed and described in Appendix S1.

### Application to Malaria Time Series Analysis

**Figure 1:**
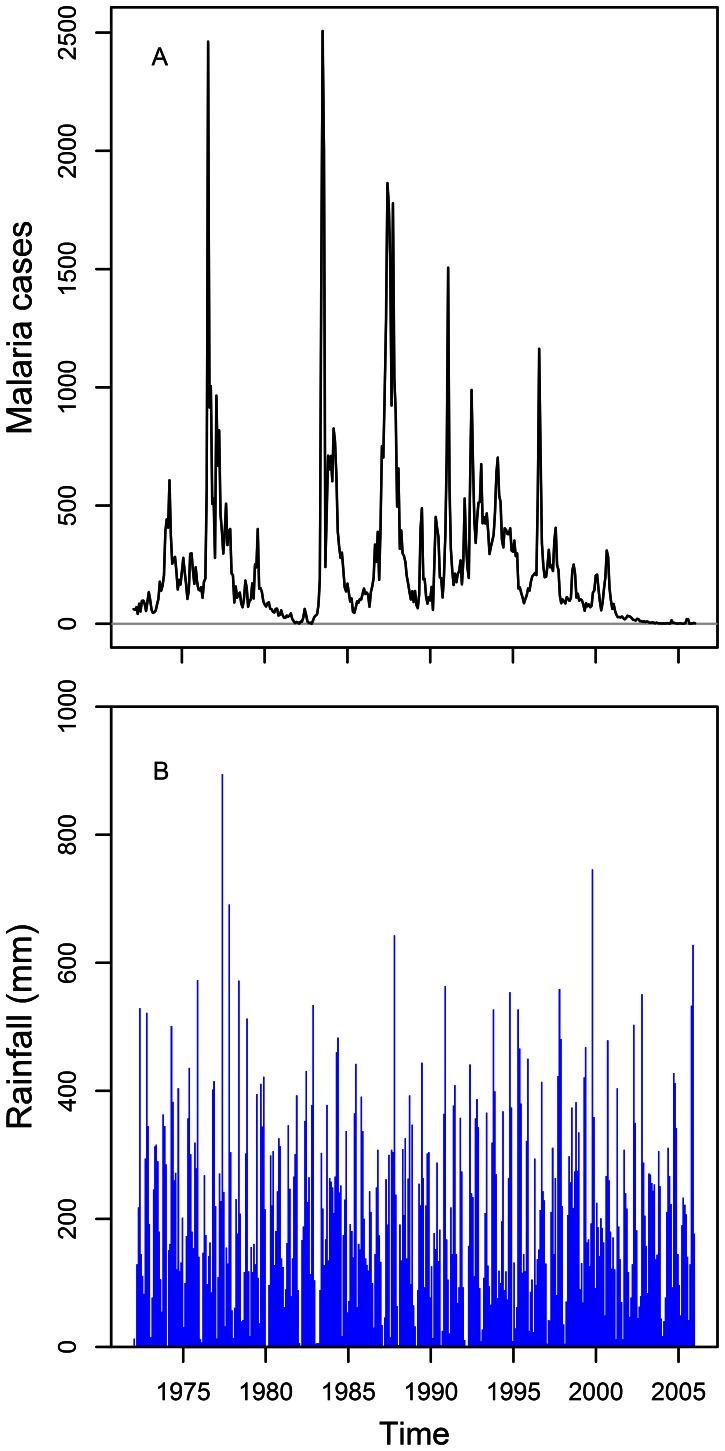
Monthly malaria case counts and rainfall in Gampaha District over time. Panel A shows monthly malaria case counts and panel B shows monthly rainfall.

This section provides an example of a GSARIMA model applied to monthly malaria case count 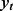 for the period 1972–2005 in the district of Gampaha in Sri Lanka (Figure 1A), with rainfall as covariate (Figure 1B). Code of the analysis is provided as supporting information in Additional File S2. Records of malaria positive blood films were reported monthly by government health facilities and aggregated by the Anti Malaria Campaign (AMC) of Sri Lanka. Rainfall was the monthly district average height of the precipitation column, which was derived from monthly island-wide precipitation surfaces. These rainfall surfaces were generated by spatial interpolation of precipitation records collected by 342 stations across the island. The data was earlier described in previous work [8]. The time series of 408 months contained three months with zero malaria cases: October 1982, and March and August 2005. Rainfall slightly improved malaria prediction by Gaussian SARIMA models fitted to logarithmically transformed malaria case data three to four months ahead [2].

### Preliminary Frequentist Gaussian SARIMA Model Identification

Because Bayesian model fit using MCMC algorithms is computationally expensive, preliminary model identification to choose the SARIMA parameters, *p*, *d*, *q*, *P*, *D*, and *Q*, was performed using standard (frequentist) tools developed for time series with Gaussian marginal errors, rather than through fitting many possible MCMC models. A visual analysis of the malaria time series (Figure 1) detected the presence of a long-term (inter annual) change in the mean level, an unstable variance (which appears to increase with the mean), and multiplicative seasonality (the size of the seasonal effect is proportional to the mean). Thus, for the preliminary Gaussian analysis, the data was transformed using a fitted Box-Cox transformation [39], in order to stabilize the variance, to make the seasonal effect additive, and to make the data approximately normally distributed [40]. The trend in the Box-Cox transformed series was treated as a stochastic trend, which was (first order) difference stationary. The augmented Dickey – Fuller test [41] on a lag order of 15 was used to detect the presence of a unit root, to assess whether the series needed to be integrated (differenced). Gaussian SARIMA models and ARIMA models with a second order harmonic seasonal component, both with *d* = 1 because of the presence of a unit root, were fitted with the (frequentist) R software package ‘stats’, and models were evaluated based on Akaike’s information criterion (AIC). The covariate matrix for the seasonal effect using second order harmonics (*i.e.* using two sine and cosine pairs) is given by 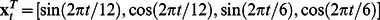. A (time independent) intercept was not included because the intercept drops out of the equation after first order differencing.

### GSARIMA Model Selection

Bayesian negative binomial versions of four SARIMA models and two ARIMA models, with second order harmonics identified in the preliminary analysis, were implemented in JAGS on untransformed data, using a logarithmic link function and ZQ1 transformation. Since there were only three observations with zero counts, the results would not be sensitive to the choice of the transformation constant for ZQ1 and this was set at c = 1. Also, versions with identity link were considered. Models were evaluated based on two criteria. The first was the deviance information criterion (DIC), which was calculated as the mean of the posterior distribution of the deviance conditional on the first 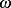 observations (with 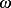 equal to the maximum *w* of the models compared), augmented with the number of effective estimated parameters as penalty to prevent over fitting. Models with lower DIC are considered to have a better fit. A second criterion was defined as the mean absolute relative error of fitted values (MARE): MARE = 
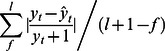
, where 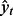 is the fitted number of malaria cases at discrete time interval *t*, and *f* and *l* are the first and last discrete time intervals, respectively, of the time period under consideration.

The MARE was calculated for both the entire series (except for the first 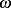 observations), when models were fitted to the entire time series (*f* = 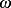
+1, *l* = n = 408), and for the second half of the time series (*f* = 205, *l* = 408), when models were fitted to the first half of the time series only.

Since the (posterior) predictive distributions estimated at each fitted data point were skewed, the median of the posterior distribution was taken for 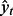. The MARE is similar to the mean absolute percentage error (MAPE), which is applicable to series for which the variance is dependent on the mean [40]. However, since the denominator is equal to or larger than one, this prevents problems with large values caused by dividing by small numbers, and a major critique of the MAPE [5]. The MARE statistic does not have a built-in penalty to prevent over fitting, but among models with similar value of MARE, the model with the least number of parameters is preferred. The MARE estimate is comparable across models with different distributional assumptions, in contrast to the DIC. Models were run with three Markov chains of 11,000 iterations each including a burn-in of 1,000 iterations. Convergence was assessed by studying plots of the Gelman-Rubin convergence statistic (on estimated parameters), as modified by Brooks and Gelman [42].

### Residual Analysis

Knowing whether the selected models and their underlying distributions fit the variation in the data adequately is of interest. If these models are used to predict malaria cases in a discrete time interval (in this case, a month), then not only is the point estimate of the posterior predictive distribution of interest, but also the entire distribution. Let 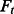 be the cumulative posterior predictive distribution function of 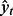. The lower tail residual probability 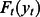, *i.e.* the value of the cumulative posterior predictive distribution calculated at the observed data 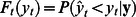, also called the probability integral transform, can be calculated for each month 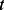. A cumulative distribution function of 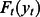 for all months of interest allows for analysis of the appropriateness of the model including the assumed underlying distribution. If the model fits the data appropriately, this ‘cumulative distribution function of residual probability values (C-R plot)’ will follow an approximately straight diagonal line between the origin and point (1,1), similar to a Probability-Probability plot. For example, when the model fits appropriately, 50% of observations have an associated residual probability value of 0.5. More detail about the C-R plot is given as supporting information [see Additional file S3]. An example is also given in the supporting information where C-R plots are used to assess appropriateness of models fitted to a time series with a Poisson GARIMA(1,1,0) structure [see Additional file S4].

Thus, after fitting a model and obtaining posterior distributions, the 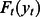 was calculated for each observation. Because of the fact that the cumulative distribution function for the negative binomial models is discrete, the residual probability value was randomized by drawing a random value 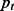 from the uniform distribution in the interval 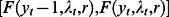, following a procedure by Dunn and Smyth [43], where 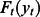 was estimated with 30,000 samples from this distribution. This procedure is advocated by Benjamin and colleagues [16] for discrete GARMA models. The appropriateness of selected models was compared using plots of their cumulative distribution functions of (randomized) residual probability values, both on the entire malaria case time series and on a period comprising the last 50 observations, where case numbers were relatively low.

It is standard practice to test time series model residuals for remaining autocorrelation. However, standard tools presume approximately Gaussian distributed data. Therefore, the randomized residual probability values were converted into normalized randomized quantile residuals, 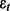, using the quantile function (inverse cumulative distribution function) of the normal distribution with zero mean and unity variance. Prior to conversion, randomized residual probability values of zero (when all 30,000 samples from the posterior predictive distribution function were above the observed value) were set to 0.00001 and randomized residual probability values of one (when all 30,000 samples from the posterior predictive distribution function were below the observed value) were set to 0.99999. The normalized randomized quantile residuals were analysed for remaining autocorrelation with the Ljung-Box test [44] and visual analysis of autocorrelation and partial autocorrelation functions.

## Results and Discussion

For the purpose of Gaussian SARIMA model identification, a Box-Cox transformation was identified by fitting to the malaria case count time series. The fitted Box-Cox parameters were a power of 0.249 and, given that the series contained observations with zero counts, a constant of 0.0251 was added to each observation prior to transformation. As observed for the original series, the presence of long-term change in the mean level was apparent in the transformed time series (Figure S1). Although the changes in the mean level could potentially be related to malaria control efforts, development of parasite and vector resistance, etc., such covariate data were not considered here.

The augmented Dickey – Fuller test supported the presence of a unit-root (p = 0.14) in the Box-Cox transformed series and the series was differenced. Plots of the auto correlation function (ACF) (Figure S2) and the partial auto correlation function (PACF) (Figure S3) of the differenced series showed significant (partial) auto correlation at lags of three and twelve months. Based on the preliminary analysis of the Box-Cox transformed series, four Gaussian SARIMA models and two Gaussian ARIMA models with second order harmonics (SOH) were initially selected, based on AIC (Table 1). ARIMA-SOH models had the lower (better) AIC compared to SARIMA models. ARIMA-SOH models including rainfall as a covariate had a slightly lower AIC than ARIMA-SOH models without rainfall. However, for the SARIMA models, the inverse was true.

**Table 1:** Akaike’s information criterion (AIC) for selected (Gaussian) models on Box-Cox transformed data.

Model	Excluding rainfall	Including rainfall
SARIMA(3**′**,1,0)×(1,0,0)_12_	1638.61	1640.35
SARIMA(3**′**,1,0)×(0,0,1)_12_	1638.95	1640.74
SARIMA(0,1,3**′**)×(1,0,0)_12_	1638.44	1640.36
SARIMA(0,1,3**′**)×(0,0,1)_12_	1638.79	1640.74
ARIMA(3**′**,1,0)-SOH	1632.2	1630.68
ARIMA(0,1,3**′**)-SOH	1631.27	1630.07

Legend: SOH: second order harmonics. For all these models, where applicable, the autoregrdessive (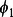 and 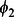) or moving average parameters (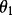 and 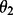) corresponding to the first two lags were omitted.

Bayesian negative binomial variants of these selected models were built. In order to establish 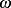, the model with the largest lag required, *w,* needed to be identified for comparison of the DIC of these Bayesian models. This was the model 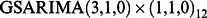
with *w* = 16. Models with logarithmic link function performed better than models with identity link. Based on the DIC, the best negative binomial model was the negative binomial 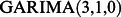 model with parameters for the first two lags (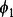 and 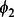) omitted (fixed to zero), with deterministic harmonic seasonality and with rainfall preceding malaria with two months (Table 2). This model also had the best overall MARE. The parameter and deviance estimates for this model, henceforth “
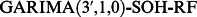
”, are detailed in Table 3. However, based on the MARE on the out of sample predictions for the second half of the time series, when the model was fitted to the first half, the negative binomial 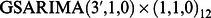
model (the ‘prime’ in the “
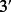
” indicating that also here the parameters for the first two lags were fixed to zero) without rainfall as covariate, was preferred. The estimates for this model, when fitted to the entire time series, are also detailed in Table 3.

**Table 2:** Selection criteria statistics for selected negative binomial models.

Model	nep	DIC on full series	MARE	DIC on first half	MARE out of sample*
GSARIMA(3**′**,1,0)×(1,0,0)_12_-IL	3	4637.8	0.4054	2266.6	0.3970
GSARIMA(3**′**,1,0)×(1,0,0)_12_-LL	3	*4350.7*	0.3883	2243.2	**0.3638**
GSARIMA(3**′**,1,0)×(0,0,1)_12_-LL	3	4351.1	0.3898	2243.0	0.3684
GSARIMA(0,1,3**′**)×(1,0,0)_12_-LL	3	4352.4	0.3883	2243.7	0.3661
GSARIMA(0,1,3**′**)×(0,0,1)_12_-LL	3	4352.8	0.3882	2243.1	0.3669
GSARIMA(3**′**,1,0)×(1,0,0)_12_-RF-LL	4	4352.5	0.3876	2240.0	0.3795
GSARIMA(3**′**,1,0)×(0,0,1)_12_-RF-LL	4	4352.9	0.3896	2240.6	0.3726
GSARIMA(0,1,3**′**)×(1,0,0)_12_-RF-LL	4	4354.3	0.3869	4354.2	0.3721
GSARIMA(0,1,3**′**)×(0,0,1)_12_-RF-LL	4	4355.0	0.3893	2241.1	0.3775
GARIMA(3**′**,1,0)-SOH-LL	6	4335.7	0.3933	2246.2	0.3796
GARIMA(0,1,3**′**)-SOH-LL	6	4336.5	0.3910	2246.1	0.3750
GARIMA(3**′**,1,0)-SOH-RF-IL	7	4399.8	0.4000	2212.3	0.3979
GARIMA(3**′**,1,0)-SOH-RF-LL	7	**4333.3**	**0.3862**	2236.7	0.3859
GARIMA(0,1,3**′**)-SOH-RF-LL	7	4333.8	0.3899	2237.1	0.3845

Legend: IL: identity link; LL: logarithmic link function with transformation method “ZQ1” corresponding to equation 2.2 in Zeger and Qaqish [24] and with 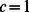; nep: number of estimated parameters; DIC: Deviance Information Criterion; MARE: mean absolute relative error of fitted values; RF: with rainfall lagged at two months; SOH: second order harmonics; *: The ‘MARE out of sample’ was calculated for the second half of the series, with the model fitted to the first half of the series only. For all models, where applicable, the autoregressive (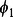 and 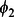) or moving average parameters (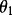 and 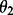) corresponding to the first two lags were omitted.

**Table 3:** Parameter estimates (mean and 95% credible interval) of selected negative binomial models.

Parameter	GARIMA(3′,1,0)-SOH-RF	GSARIMA(3′,1,0)×(1,0,0)_12_
*β*_rain_	−0.34 (−0.66, −0.02)	
*β*_sin(2πt/12)_	−0.10 (−0.23, 0.02)	
*β*_cos(2πt/12)_	−0.15 (−0.28, −0.02)	
*β*_sin(2πt/6)_	0.14 (0.06, 0.21)	
*β*_cos(2πt/6)_	0.16 (0.07, 0.24)	
*φ*_3_	−0.10 (−0.19, 0.00)	−0.13 (−0.23, −0.04)
*φ*_1_ ^*^		0.12 (0.03, 0.22)
*ψ*	4.54 (3.87, 5.27)	4.32 (3.69, 5.04)
*Amplitude AH^$^*	0.19 (0.07, 0.32)	
*Amplitude SAH^$^*	0.21 (0.13, 0.29)	
*Phase shift AH^$^*	4.83 (3.30, 6.35)	
*Phase shift SAH^$^*	−0.69 (−1.05, −0.34)	

Legend: GARIMA(3**′**,1,0)-SOH-RF = GARIMA(3,1,0) model with parameters for the first two lags (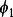 and 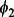) omitted, second order harmonics and rainfall lagged at 2 months (in m); GSARIMA(3**′**,1,0)×(1,0,0)_12_ = GSARIMA(3,1,0)×(1,0,0)_12_ model with parameters for the first two lags (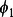 and 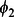) fixed to zero; AH = annual harmonic, SAH = semi-annual harmonic; ^$^ = derived parameter, phase shift = phase shift of the cosine function expressed in months.

Despite the 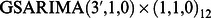 model having a higher (worse) DIC than the 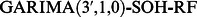 model, the out of sample MARE of the 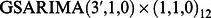 model was 5.7 per cent better than the out of sample MARE of the 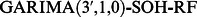 model, and required less than half the number of fitted parameters. This indicates that the 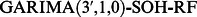 model was probably over-fitting the data, describing the random error rather than the underlying process. The 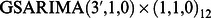 model was selected for further analysis.

**Figure 2:**
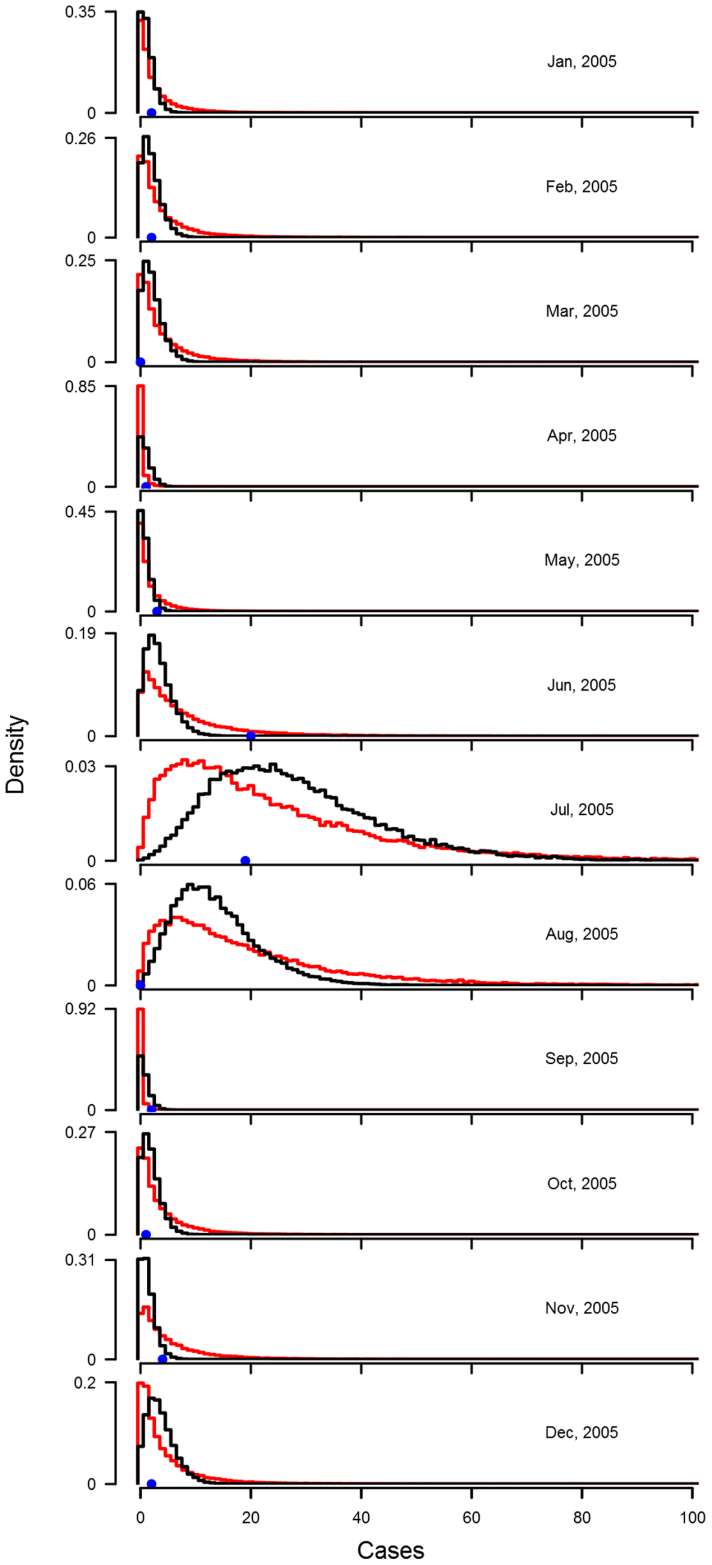
Posterior predictive distributions for the last 12 months of the Gampaha malaria case count series. In each panel, representing each a month in the last year of the series, the black and the red lines are the outline histogram of the density of the posterior predictive distribution of the negative binomial 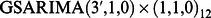 model and a (Bayesian) Gaussian 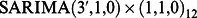 model on Box-Cox transformed data, respectively. Models were fitted to the entire data set. In each panel, the observed case count is represented by a blue dot.

Figure 2 illustrates posterior predictive distributions for the last 12 months of the series by the 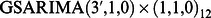 model and those by a (Bayesian) Gaussian 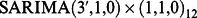 model on Box-Cox transformed data, when fitted to the entire data set. Differences in the posterior predictive distributions between the two models are apparent with the Gaussian model predictive distributions having longer right tails.

**Figure 3:**
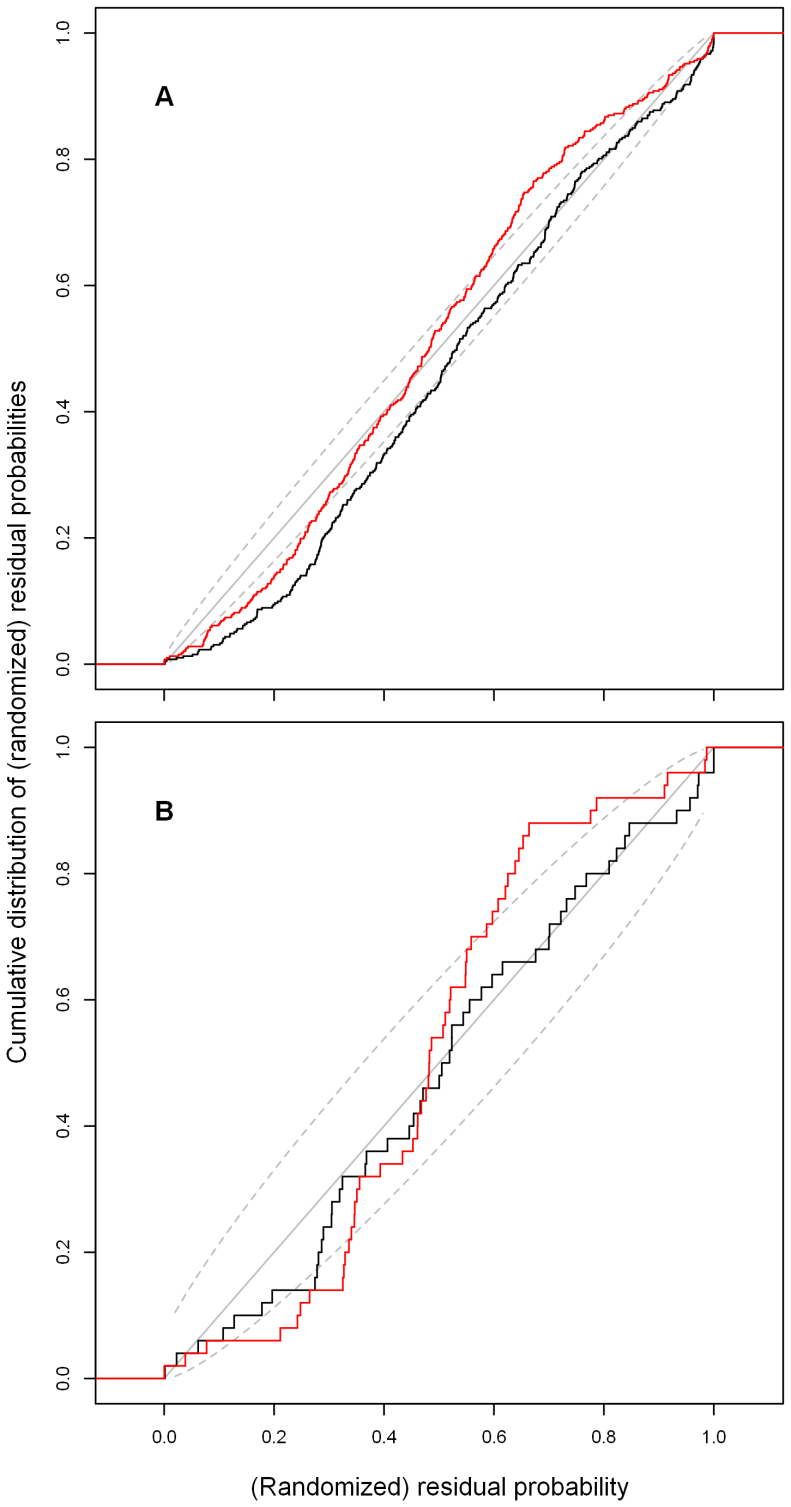
Cumulative distribution function of randomized cumulative probabilities. The black line represents the cumulative distribution function of randomized cumulative probabilities of the 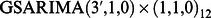 model on monthly numbers of malaria cases in Gampaha, Sri Lanka. The red line represents the cumulative distribution function of randomized residual probabilities of the Gaussian 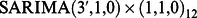 model on Box-Cox transformed data. The light grey diagonal line (cumulative distribution equals randomized probability) represents on average appropriate predictive distributions. Dotted lines represent 95% confidence boundaries for proportions equalling probability. **A**: for the last 392 months in the series. **B**: for the last fifty months in the series.

The C-R plot of the negative binomial 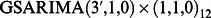
model fit was compared to that of a (Bayesian) Gaussian 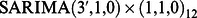 on Box-Cox transformed data in Figure 3. The C-R plot on the entire series (Figure 3A) is not entirely satisfactory for either model. For the Gaussian 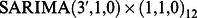, the posterior predictive distribution appears to be platykurtic (for values of the residual probability below 0.5, there are too few observations, and for values above 0.5, there are too many). For the negative binomial 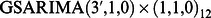
model, for randomized residual probability values below about 0.5, cumulatively fewer observations had these values than the posterior density distributions had indicated. Therefore, on average, the part of the posterior density distributions below the median was spread out too much to the left. The lower boundaries of credibility intervals of the distributions were thus on average too low. For the values above 0.5, the cumulative distribution function followed the diagonal. Figure 3B compares both models for the last 50 months of the series only, where numbers of monthly cases were smaller than 35. For these low numbers, the negative binomial 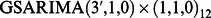 model was much more appropriate.

**Figure 4:**
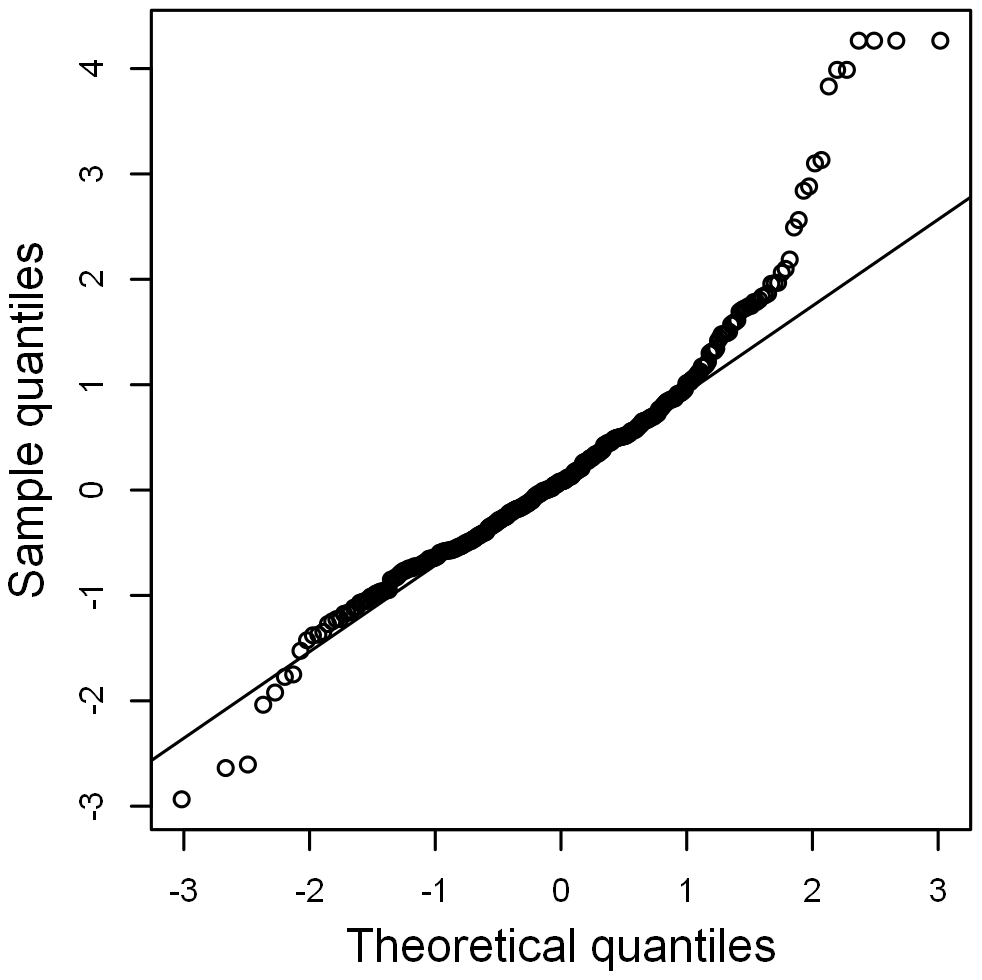
Normal Q-Q plot of normalized randomized quantile residuals of the selected 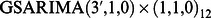 model.

**Figure 5:**
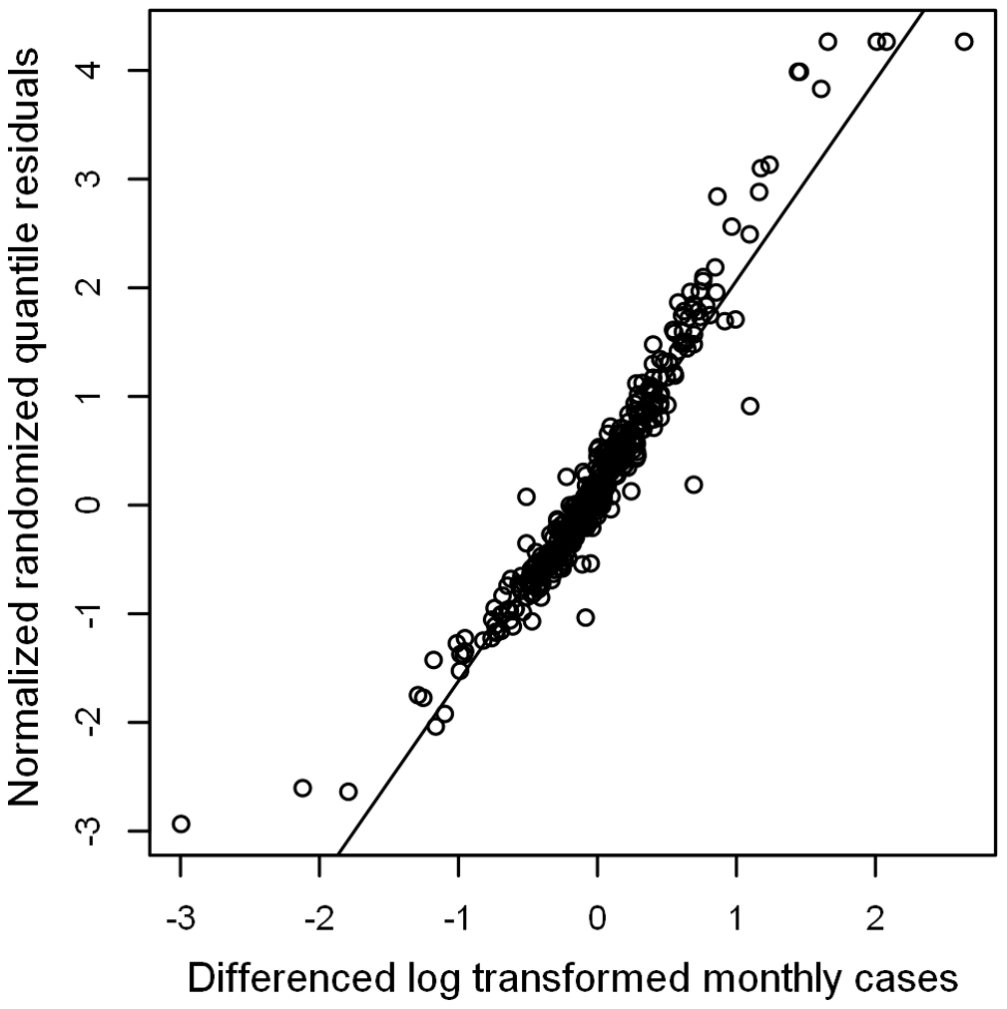
Plot of normalized randomized quantile residuals of the 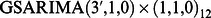 model against the logarithm of relative change. Monthly malaria case counts were logarithmically transformed after adding one. Then for each month, the difference between this value and the value for the previous month was taken. The diagonal is the fitted regression line.

Figure 4 shows the normal Q-Q plot for the normalized randomized quantile residuals of the 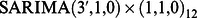 model, for which the distribution is slightly leptokurtic. A plot of these normalized randomized quantile residuals against time (Figure S4) appears a random scatter at first sight, but upon closer inspection, extreme residuals occur more often during periods with stronger relative changes. This is because the residuals, 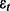, are positively correlated with a relative change in malaria cases, with linear regression line 
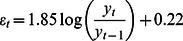
, 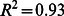 (Figure 5).

**Figure 6:**
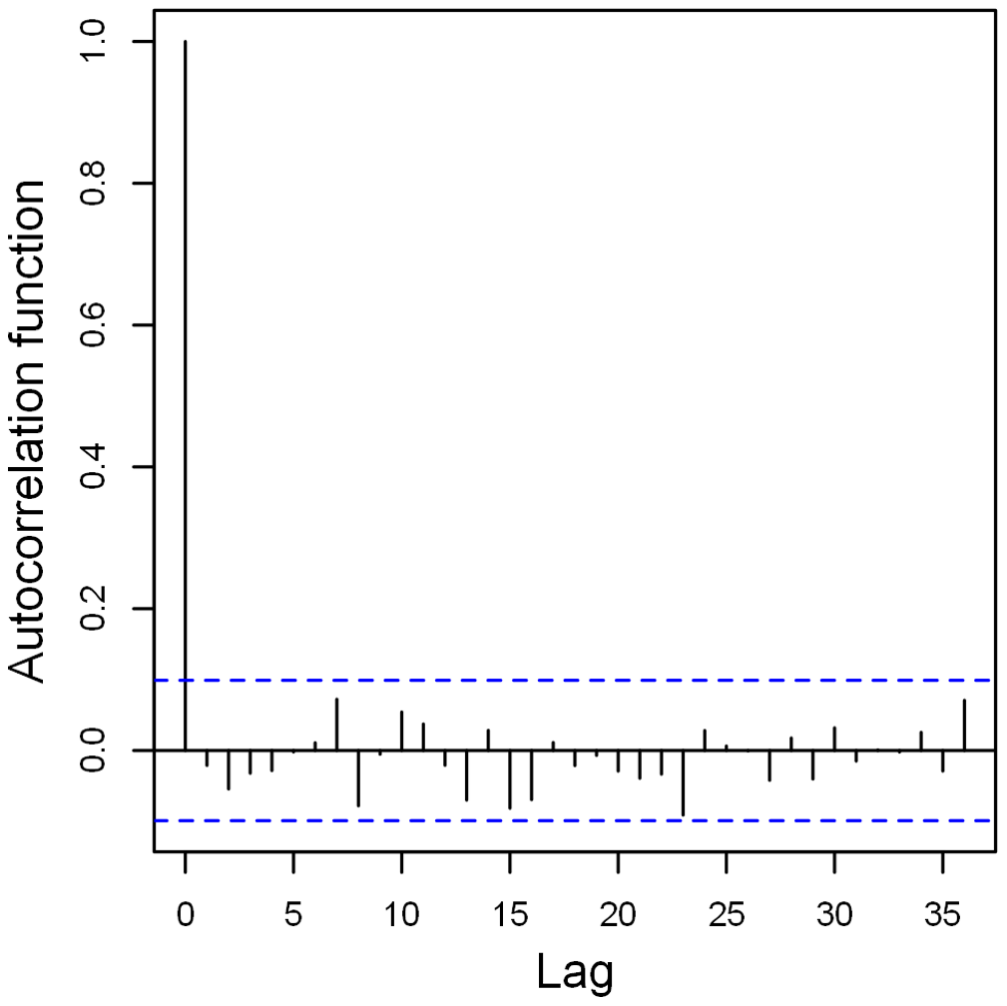
Plot of the autocorrelation function of normalized randomized quantile residuals of the selected 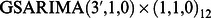
model.

The fact that this line does not go through the origin but has a (small but significant; p<0.05) positive intercept is another indication that the posterior distributions have, on average, too much mass to the left, and therefore, on average, overestimate the residuals. Figure 6 shows a plot of the autocorrelation function of the normalized randomized quantile residuals of the 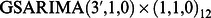 model. There is no indication of significant autocorrelation in the residuals, which was confirmed by the Ljung-Box test [44]. The Ljung-Box statistic was 19.8 based on 24 lags, which was not significant (p = 0.65) because the quantile corresponding to the 95^th^ percentile of a chi-squared distribution with 23 degrees freedom (24 degrees minus one fitted ARMA parameter) is 35.17. The Ljung-Box test is valid under these mild conditions of non-normality, although for stronger non-normality, the Ljung-Box test is not robust and tends to reject the null hypothesis of no autocorrelation too quickly [45].

### Conclusions

To model a series of monthly counts of new malaria episodes in a district in Sri Lanka, GSARIMA models and GARIMA models with a deterministic seasonality component were developed. GSARIMA and GARIMA models are an extension of the class of GARMA models [16], and are suitable for parsimonious modelling of non-stationary seasonal time series of (over dispersed) count data with negative binomial conditional distribution.

Models were presented with a choice of identity link function or logarithmic link function, and for the latter models, with a choice between two transformation methods to deal with zero value observations and using a threshold parameter. When a count time series has many observations of zero, both transformation methods and several threshold parameters should be explored in order to find the best fitting model.

Bayesian GSARIMA and GARIMA models were applied to malaria case count time series data from Gampaha District in Sri Lanka. Both a GSARIMA and a GARIMA model with a deterministic seasonality component were selected, based on different criteria. The GARIMA model with deterministic seasonality showed a lower DIC, but the GSARIMA model had a lower mean absolute relative error on out of sample data, and needed fewer parameters. Bayesian modelling allowed for analysis of the posterior predictive distributions. The performance of the selected negative binomial model was compared with that of a Gaussian version of the model on Box-Cox transformed data. These distributions did not perfectly mirror the distribution of the residuals for either model. This is possibly an indication that the assumptions about the underlying distributions were not entirely appropriate for either case. However, analysis of the residuals showed that the posterior predictive distributions were much better for the negative binomial GSARIMA model than for its Gaussian version on transformed data when counts were low. Both models could account for autocorrelation in the data, but the negative binomial model had an 8% better MARE than the Gaussian version on transformed data (0.388 *vs* 0.423).

The fact that the cumulative distribution functions do not perfectly match the diagonal in Figure 3A indicates that there is room for improvement, through modelling a more complex autocorrelation structure (*e.g.* through time varying SARIMA parameters) and through the inclusion of covariates. It is also possible that assuming an underlying negative binomial distribution is not entirely appropriate. In the latter case, the DIC, which was based on this assumption, has less value than the MARE for comparison between models. Apart from the fact that the MARE does not depend on the assumption of a true underlying distribution, it is easier to for malaria control staff to interpret.

G(S)ARIMA models may be particularly useful in the drive towards malaria elimination, but could also be applied to other fields. Although building and fitting Bayesian GSARIMA models is laborious, they may provide more realistic prediction distributions for time series of counts than do Gaussian methods on transformed data, especially when counts are low.

## Supporting Information

Figure S1 **Box-Cox transformed monthly malaria case counts in Gampaha.**
(PDF)

Figure S2 **Autocorrelation function of Box-Cox transformed monthly malaria case counts in Gampaha.**
(PDF)

Figure S3 **Partial autocorrelation function of Box-Cox transformed monthly malaria case counts in Gampaha.**
(PDF)

Figure S4 **Normalized randomized quantile residuals of negative binomial GSARIMA(3′,1,0)×(1,0,0)_12_ model.**
(PDF)

Additional File S1 **R code example for simulating and estimating a time series with GSARIMA(2,1,0)x(0,0,1)_12_-x structure, and one with GSARIMA(0,1,2)x(1,0,0)_12_-x structure.**
(RTF)

Additional File S2 **R code for the analysis of monthly malaria case count data for the district of Gampaha, Sri Lanka.**
(RTF)

Additional File S3 **R code for examples illustrating how plots of a cumulative distribution function of residual probability values, here called "C-R plots" can be used to estimate the appropriateness of the posterior predictive distributions**
(RTF)

Additional File S4 **R code for an example of simulating and estimating a time series with a Poisson GARIMA(1,1,0) structure, and the use of C-R plots to estimate the appropriateness of the posterior predictive distributions, comparing Poisson and Gaussian models fitted to Poisson data.**
(RTF)

Additional File S5 **R code for an example of simulating and estimating a time series with a Poisson GARIMA(1,0,0) structure, and the effects of misspecification.**
(RTF)

Appendix S1 **Appendix.**
(PDF)
