# Supplementary figures and images for: Generalized Seasonal Autoregressive Integrated Moving Average Models for Count Data with Application to Malaria Time Series with Low Case Numbers
Source: PLoS One. 2013 Jun 13;8(6):e65761. doi: 10.1371/journal.pone.0065761 (PMC3681978; doi:10.1371/journal.pone.0065761)

**Box-Cox transformed monthly malaria case counts in Gampaha**

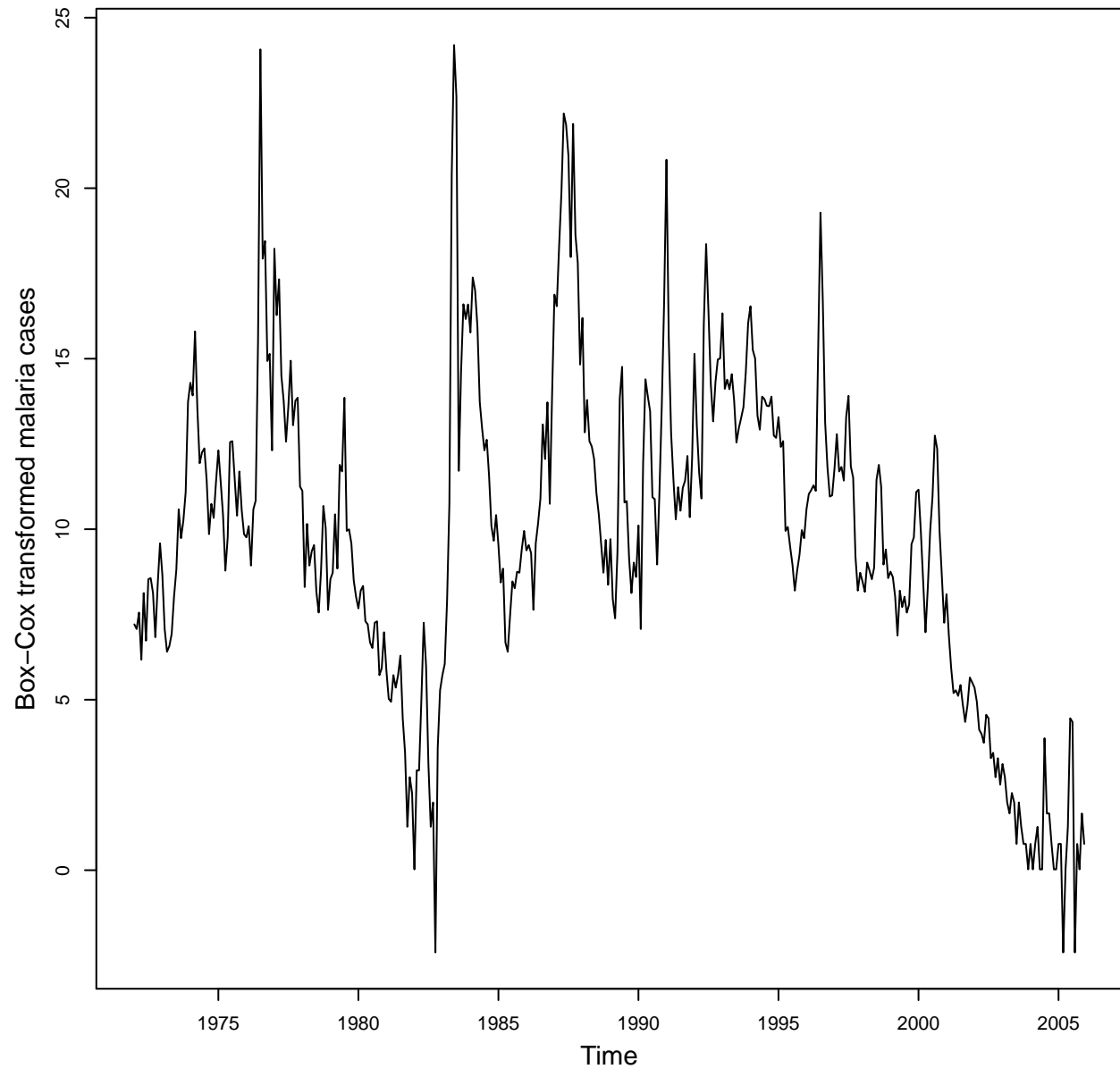

Supplement: Figure S1 — Box-Cox transformed monthly malaria case counts in Gampaha. (PDF) [file pone.0065761.s001.pdf]

Autocorrelation function of Box-Cox transformed monthly malaria case counts in Gampaha

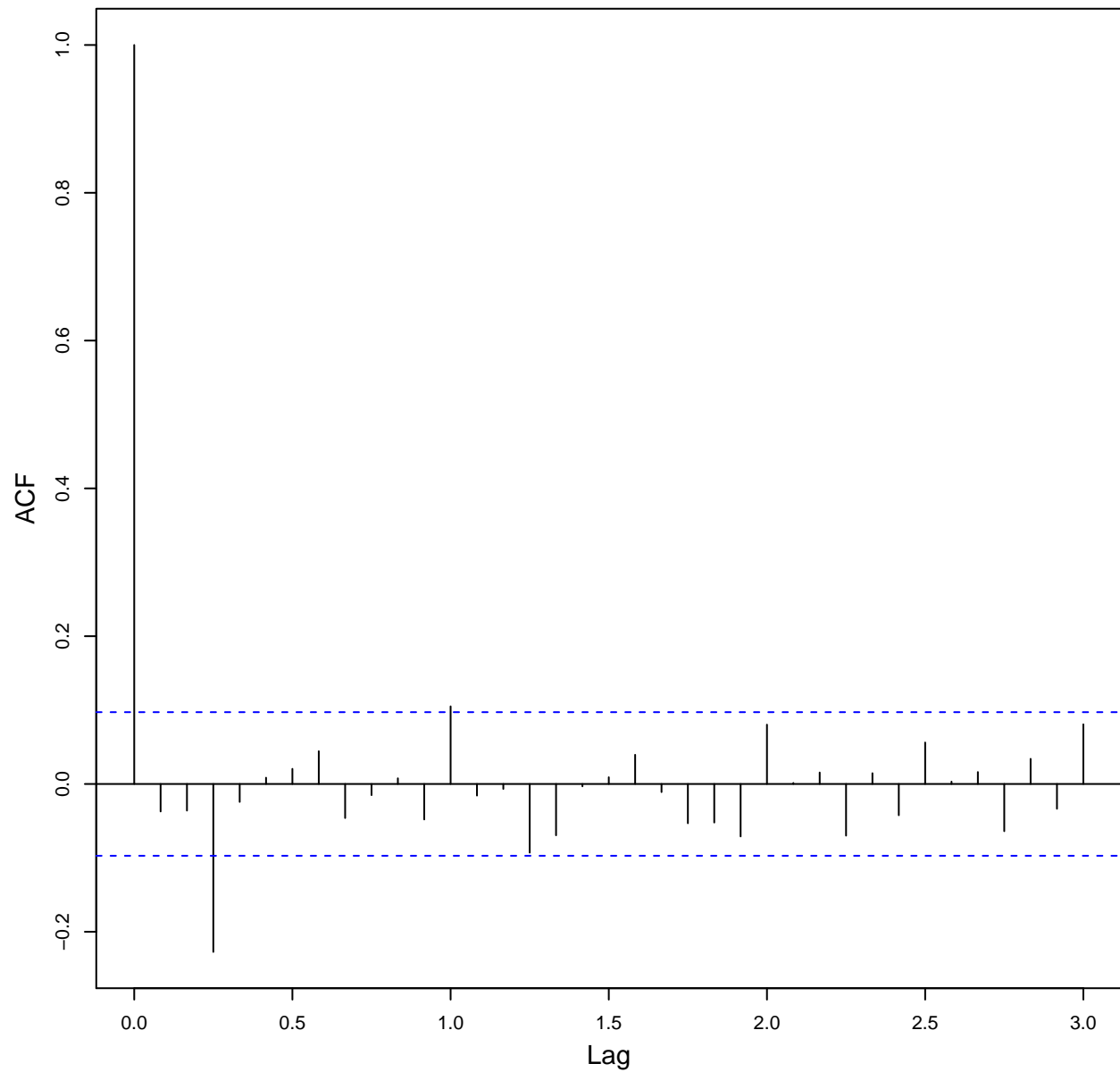

Supplement: Figure S2 — Autocorrelation function of Box-Cox transformed monthly malaria case counts in Gampaha. (PDF) [file pone.0065761.s002.pdf]

Normalized randomized quantile residuals of negative binomial GSARIMA (3', 1, 0)  $\times$  (1, 0, 0)<sub>12</sub> model

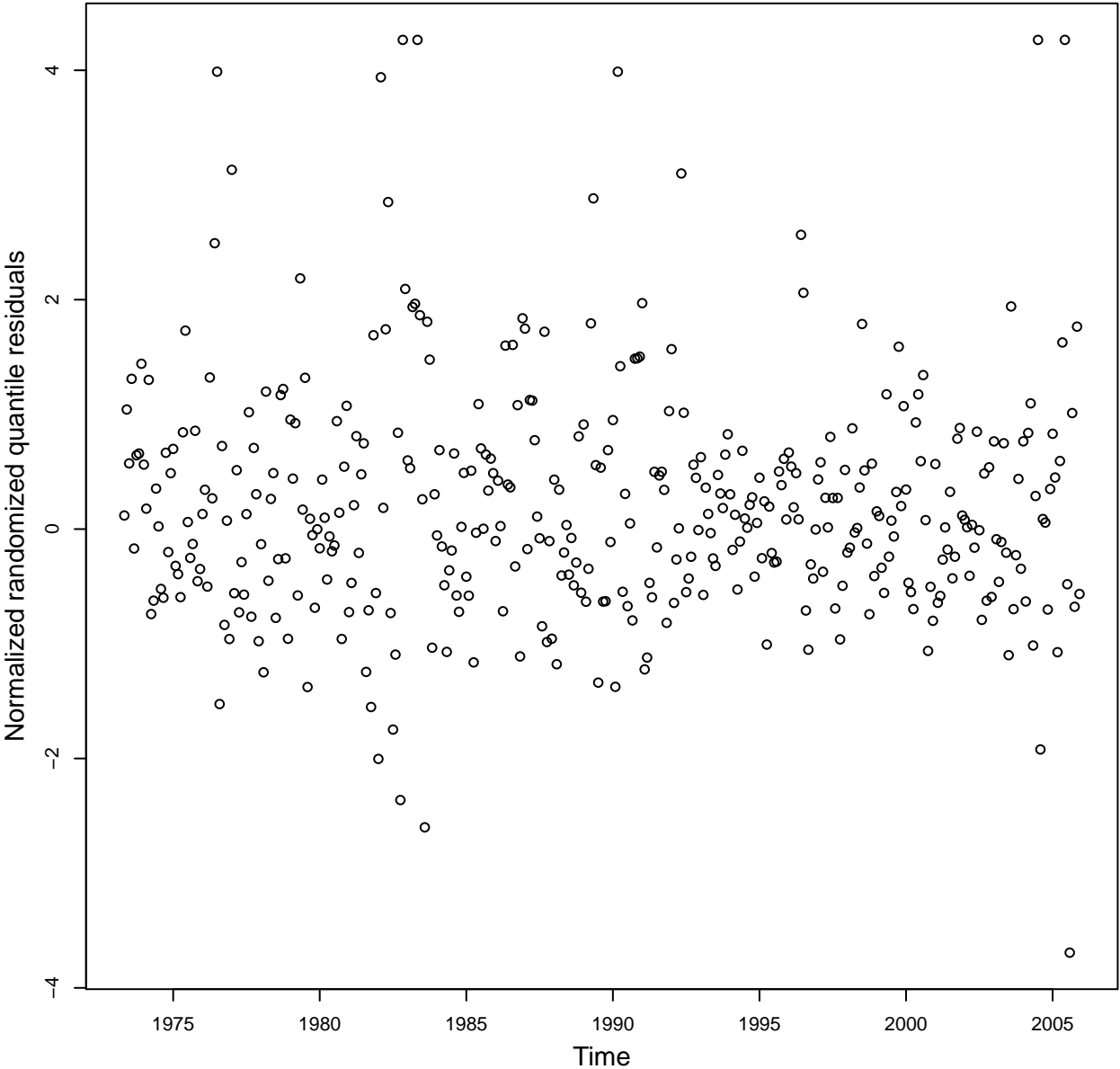

Supplement: Figure S4 — Normalized randomized quantile residuals of negative binomial GSARIMA(3′,1,0)×(1,0,0)12 model. (PDF) [file pone.0065761.s004.pdf]
