# Supplementary material for: Generalized Seasonal Autoregressive Integrated Moving Average Models for Count Data with Application to Malaria Time Series with Low Case Numbers
Source: PLoS One. 2013 Jun 13;8(6):e65761. doi: 10.1371/journal.pone.0065761 (PMC3681978; doi:10.1371/journal.pone.0065761)

Partial autocorrelation function of Box−Cox transformed monthly malaria case counts in Gampaha

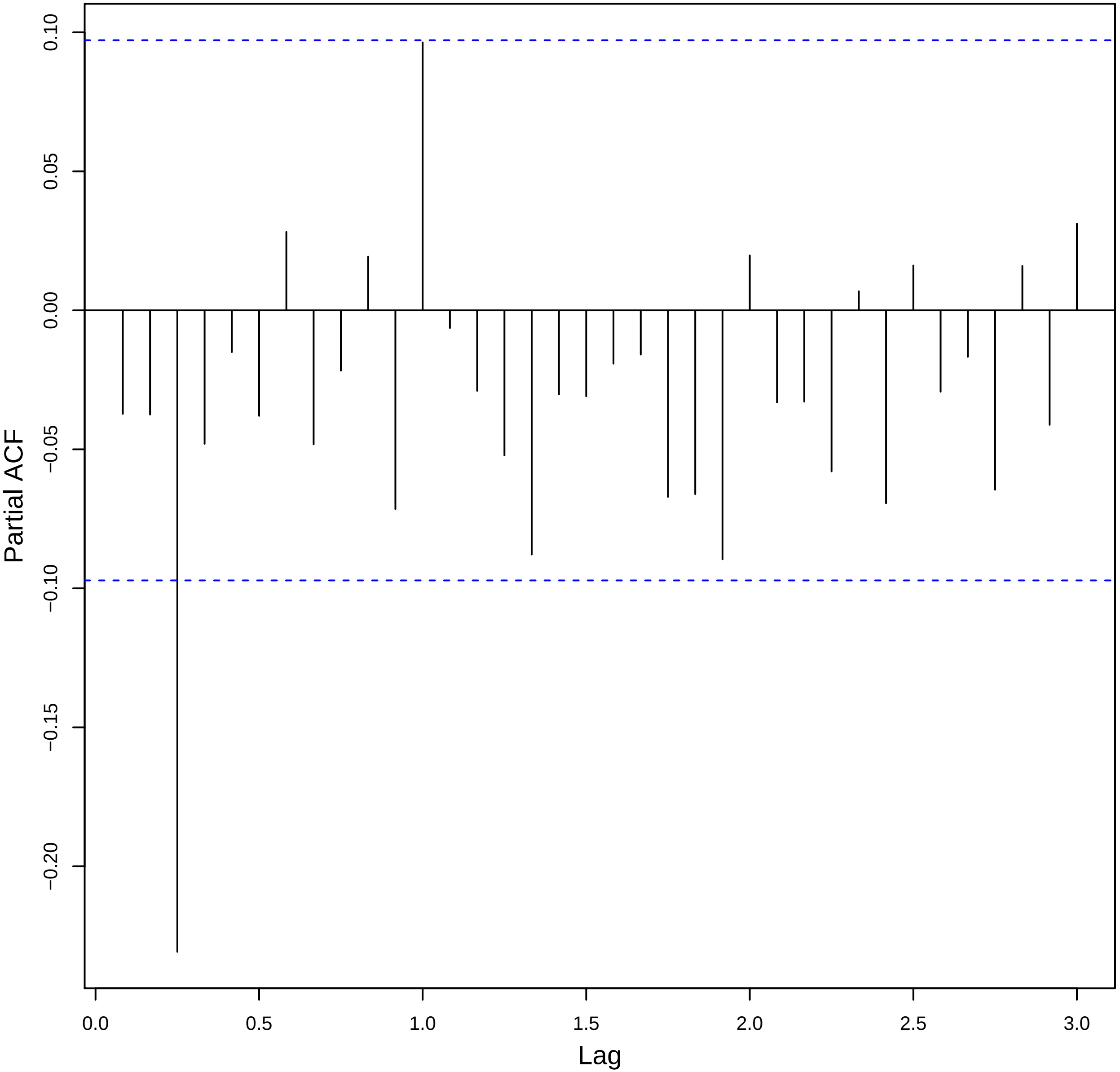

Supplement: Figure S3 — Partial autocorrelation function of Box-Cox transformed monthly malaria case counts in Gampaha. (PDF) [file pone.0065761.s003.pdf]
