# Supplementary material for: Generalized Seasonal Autoregressive Integrated Moving Average Models for Count Data with Application to Malaria Time Series with Low Case Numbers
Source: PLoS One. 2013 Jun 13;8(6):e65761. doi: 10.1371/journal.pone.0065761 (PMC3681978; doi:10.1371/journal.pone.0065761)
Supplement: Additional File S1 — R code example for simulating and estimating a time series with GSARIMA(2,1,0)x(0,0,1)12-x structure, and one with GSARIMA(0,1,2)x(1,0,0)12-x structure. (RTF) [file pone.0065761.s005.rtf]

Additional file S1
##Additional File S1, supporting information to Briët et al.: “Generalized seasonal autoregressive integrated moving average models for count data with application to malaria time series with low case numbers”
##This file contains computer code for use in the free software R [http://cran.r-project.org/]. It gives an example of simulating and estimating a time series with GSARIMA(2,1,0)x(0,0,1)12-x structure, and one with GSARIMA(0,1,2)x(1,0,0)-x structure. For the code to run, it requires a few R packages, and JAGS [http://mcmc-jags.sourceforge.net/] to be installed. 

##Example of GSARIMA(2,1,0)x(0,0,1)12-x
phi<-c(0.5,0.2)
theta<-c(0)
Theta<-c(0.5)
Phi<-c(0)
d<-1
D<-0
frequency<-12
ar<-arrep(phi=phi, theta=theta, Phi=Phi, Theta=Theta, frequency= frequency, d=d, D=D)
N<-1000
intercept<-10
theta<-5
x<- rnorm(N)
beta.x<-0.7
X<-matrix(c(rep(log(intercept), N+length(ar)), rep(0, length(ar)), x), ncol=2)
c<-1
y.sim <- garsim(n=(N+length(ar)), phi=ar, beta=c(1,beta.x), link= "log", family= "negative.binomial", zero.correction = "zq1", c=c, theta=theta, X=X) 
y<-y.sim[(1+length(ar)):(N+length(ar))]
tsy<-ts(y, freq=frequency)
plot(tsy)
plot(log(tsy))

#cat(y, sep=", ") ##These simulated data were used in analysis in the appendix
#y<-c(6, 1, 3, 1, 0, 1, 0, 0, 4, 10, 20, 29, 29, 89, 101, 166, 28, 22, 3, 8, 10, 13, 3, 3, 1, 18, 0, 9, 0, 3, 1, 1, 0, 0, 3, 4, 3, 17, 111, 155, 693, 1159, 944, 1323, 9645, 748, 1714, 1324, 748, 802, 46, 50, 145, 63, 89, 16, 89, 19, 12, 3, 1, 1, 0, 0, 9, 6, 10, 12, 10, 23, 8, 51, 254, 64, 200, 78, 154, 258, 487, 979, 1536, 5750, 5548, 6146, 37779, 43717, 117723, 35824, 137352, 102898, 281734, 64783, 10241, 37001, 14486, 4837, 8313, 4231, 1896, 5312, 1223, 2023, 264, 279, 206, 67, 9, 7, 3, 5, 1, 1, 2, 1, 2, 1, 0, 1, 1, 0, 1, 0, 32, 62, 149, 465, 2844, 294, 1082, 1479, 967, 450, 2416, 4453, 7466, 4716, 6426, 5577, 3882, 5242, 809, 3170, 684, 423, 165, 51, 416, 187, 150, 132, 121, 78, 26, 33, 55, 5, 6, 8, 3, 0, 1, 1, 0, 0, 5, 7, 11, 3, 4, 0, 1, 1, 0, 1, 0, 34, 52, 452, 470, 1998, 10766, 5217, 2400, 1968, 2277, 3705, 2186, 10626, 4925, 4895, 7233, 5483, 3109, 2836, 602, 40, 23, 0, 1, 6, 9, 2, 2, 5, 0, 3, 0, 0, 2, 4, 10, 208, 44, 16, 9, 13, 2, 4, 3, 10, 23, 66, 554, 2836, 505, 1303, 601, 3198, 255, 1396, 170, 62, 35, 88, 139, 129, 55, 20, 268, 19, 99, 38, 8, 5, 1, 0, 0, 0, 1, 0, 1, 2, 0, 5, 5, 21, 5, 22, 3, 0, 2, 0, 1, 1, 1, 2, 2, 4, 2, 22, 1, 0, 0, 1, 3, 1, 3, 3, 4, 21, 94, 93, 42, 53, 470, 66, 247, 378, 814, 4339, 1088, 21351, 30033, 2166, 2266, 507, 228, 5407, 2785, 2663, 1336, 4782, 72296, 32912, 31744, 8310, 2845, 10814, 3374, 4725, 1594, 137, 78, 112, 136, 107, 124, 57, 32, 59, 28, 25, 15, 5, 9, 0, 1, 8, 0, 1, 0, 0, 23, 78, 65, 234, 1070, 85, 193, 67, 286, 160, 264, 931, 4384, 7012, 2343, 2274, 7506, 3487, 5223, 1115, 14606, 1721, 6394, 4542, 8668, 28850, 29355, 102967, 259438, 223259, 1309523, 5724889, 3159661, 2894955, 5933577, 16542145, 6900891, 9457175, 4997983, 681924, 244294, 283416, 358868, 486698, 57040, 61644, 499670, 388416, 38540, 12352, 12422, 4903, 415, 198, 204, 581, 429, 248, 756, 48, 83, 19, 22, 8, 13, 2, 2, 4, 5, 2, 6, 1, 0, 4, 12, 4, 4, 23, 17, 40, 97, 268, 117, 258, 1477, 2588, 911, 2358, 233, 962, 4394, 6224, 9807, 5895, 5398, 5384, 1984, 506, 799, 71, 168, 71, 73, 34, 6, 5, 1, 1, 0, 0, 10, 2, 3, 9, 2, 3, 0, 0, 2, 1, 11, 4, 1, 0, 3, 1, 2, 1, 22, 32, 32, 131, 249, 23, 29, 10, 1, 0, 2, 9, 23, 3, 15, 75, 51, 221, 551, 175, 44, 238, 842, 295, 280, 134, 137, 143, 144, 541, 253, 49, 105, 56, 97, 35, 122, 14, 51, 46, 15, 27, 6, 4, 0, 1, 2, 0, 1, 0, 1, 0, 4, 7, 23, 24, 1, 0, 0, 3, 1, 1, 1, 0, 4, 6, 27, 10, 18, 9, 5, 20, 6, 5, 10, 9, 5, 0, 1, 0, 2, 13, 62, 6, 5, 5, 0, 1, 0, 11, 16, 7, 33, 15, 18, 15, 2, 0, 0, 3, 111, 21, 35, 142, 246, 170, 161, 52, 122, 237, 145, 414, 183, 205, 378, 553, 888, 1085, 955, 6035, 1258, 731, 933, 505, 402, 679, 1515, 6030, 4648, 23518, 6746, 3662, 11539, 1729, 1138, 237, 868, 240, 306, 2411, 2341, 1566, 197, 74, 86, 20, 27, 1, 3, 9, 2, 1, 1, 2, 1, 2, 2, 2, 1, 0, 2, 0, 7, 2, 2, 9, 8, 1, 0, 1, 1, 3, 0, 1, 0, 2, 11, 8, 8, 16, 2, 34, 29, 9, 4, 13, 20, 15, 16, 0, 2, 1, 0, 0, 4, 5, 3, 38, 9, 22, 3, 11, 1, 2, 0, 1, 2, 0, 0, 1, 0, 0, 0, 4, 0, 0, 0, 0, 0, 12, 6, 2, 16, 4, 4, 0, 8, 22, 73, 60, 475, 133, 1757, 913, 2631, 1238, 2193, 4272, 10059, 65842, 191067, 643733, 1682433, 3415585, 17370816, 71112331, 19550241, 86132601, 322646306, 1057183452, 265519875, 462034473, 448366942, 301272664, 102779704, 47748602, 251064984, 213220075, 50858675, 20483199, 6062297, 40222414, 62155400, 39421947, 57601064, 47624320, 8653833, 5771287, 2531444, 2040600, 2001335, 2802411, 1717988, 1854774, 3574352, 188671, 221000, 67221, 61687, 113469, 208747, 121524, 82464, 35783, 19906, 18038, 12146, 1090, 82, 160, 65, 18, 1, 0, 3, 2, 0, 3, 2, 0, 1, 0, 0, 3, 1, 5, 4, 9, 44, 17, 3, 15, 48, 19, 290, 196, 263, 502, 49, 80, 16, 0, 1, 1, 4, 9, 1, 2, 1, 0, 1, 0, 0, 0, 1, 3, 2, 4, 1, 7, 5, 7, 15, 61, 5, 9, 26, 15, 6, 1, 0, 0, 0, 4, 3, 14, 25, 5, 5, 6, 1, 1, 1, 1, 2, 2, 0, 5, 8, 12, 86, 3, 2, 2, 3, 11, 7, 5, 22, 16, 3, 3, 0, 1, 1, 5, 45, 3, 16, 54, 23, 240, 217, 107, 168, 68, 218, 161, 70, 14, 13, 49, 43, 26, 28, 68, 60, 17, 7, 5, 1, 0, 0, 2, 4, 3, 1, 2, 2, 3, 0, 1, 5, 9, 25, 44, 68, 164, 165, 114, 1322, 1674, 1267, 4696, 2529, 1760, 1216, 720, 404, 690, 1460, 480, 89, 15, 18, 92, 4, 4, 7, 6, 6, 7, 16, 13, 1, 0, 2, 2, 3, 1, 5, 7, 19, 86, 58, 6, 2, 1, 0, 8, 4, 15, 10, 52, 15, 30, 13, 2, 1, 1, 43, 240, 152, 396, 211, 127, 359, 330, 301, 33, 46, 231, 256, 3, 126, 3, 7, 0, 0, 0, 0, 5, 2, 0, 5, 0, 9, 0, 3, 0, 6, 40, 56, 51, 61, 530, 244, 401, 452, 123, 98, 659, 1061, 1188, 382, 288, 92, 158, 23, 90, 69, 100, 47, 70, 60, 150, 58, 116, 22, 14) 

#cat(x, sep=", ")
#x<-c(1.123864, -0.9889375, -0.1331789, -1.082886, -0.7070339, 0.2053386, -0.2755704, -1.275805, -0.5757, 0.4458147, 0.5831621, -0.01767126, -0.1188393, -0.1362523, 0.5290077, 0.7966444, 0.1576836, 1.043497, -1.304347, -0.01205971, -0.6604155, -0.06000824, -0.2274308, -0.4986921, -0.7381525, 2.060209, -2.058291, -0.01364356, -1.584835, 0.01583264, -0.05706434, 0.8422598, 0.0877418, -0.5648019, 0.9173895, 0.8981901, -0.8441297, -0.3394345, 0.119574, -0.7908411, -0.05399792, -0.33522, -0.7988216, -0.5691593, 1.787027, -1.90904, -1.092724, 0.4053384, 0.4007372, 1.603095, -1.542842, -1.116978, 0.156364, -0.1051124, 0.6201602, -2.382055, 0.9082133, -0.1450971, 0.307575, -0.1476459, -1.750176, -0.197539, -1.237438, -1.884924, 0.9080842, 0.4917742, 0.4422944, -0.2603255, -0.02098885, 0.2849409, -1.480535, 0.3509629, 0.821517, -2.013839, -0.4200755, -2.429301, -0.8714, 0.05240783, 0.2963095, -0.04961894, 0.3511394, 0.9305478, 0.2764555, -1.415571, 0.1515853, -0.8721427, 0.911375, -0.5254322, 1.094238, -0.25529, 1.080479, -0.9441073, -2.096997, 1.111753, 0.6172078, -1.00206, 0.357432, -0.161169, -0.633728, 1.219146, 0.005326097, 0.7935907, -1.241602, -1.09559, -0.02703619, 0.1054188, -0.3991424, 0.4324588, -0.5604485, 0.05386297, -0.2016963, 1.081838, 0.806697, 0.1162551, -1.01408, -0.2727702, 1.099604, 0.272161, 2.329532, 0.08653523, -0.9496778, -2.751141, 0.5197514, 0.5075645, -0.03751165, 0.1002497, 0.4318126, -2.020999, 0.7470019, 0.1730521, 0.2786718, -0.8121039, 1.537912, 1.029731, 0.991895, -0.2670179, 1.192465, 0.4242699, 0.1734738, 0.5614674, -0.9057283, 1.478979, -0.7094664, 0.4741349, 0.08986982, -2.055693, 1.403441, -0.2550486, -0.1717027, -0.2268178, 0.6832545, 0.02326707, -1.171063, -1.177981, 0.1026343, -1.981742, -0.05404807, 0.4559685, 0.849279, -1.345044, -0.27875, -1.064493, -0.1850661, -1.209282, 0.09388464, -0.9610342, 0.1939746, -0.6691423, 0.1272827, -0.376735, 0.9668639, -0.8571353, 0.5266661, 0.08055797, -2.133661, 1.103301, -0.6358954, 0.3845638, -0.8481874, -0.02618846, 0.6816614, 0.7761569, 0.3670046, 0.5263061, 0.937241, 1.168893, -0.3234232, 0.9873871, -0.2368658, -0.2049503, 0.6019941, 0.4077145, -0.5150763, 0.440221, -0.526806, -1.178239, 1.212898, -0.9128715, -1.521923, 0.241764, 1.013117, -0.0008951717, 0.4986556, 1.040107, -0.7223109, 0.623718, 0.5391772, -1.400904, 0.1095111, 0.1772003, -0.8332139, 2.430834, 0.2020476, 0.01179089, 0.09712849, 0.8749771, -1.218111, -0.27252, -0.2018552, 0.3876274, 0.4503249, -0.3200454, 0.2363248, 1.192604, -1.332953, 0.07830725, -1.028495, 1.635571, -0.8035294, 2.159717, -0.1708336, -0.8630413, -1.441837, 0.281023, 0.6317584, 1.224217, 0.4288027, -1.255606, 2.031445, -2.466227, 0.817235, 0.6598292, -0.1352785, -0.02831207, 0.1870206, -1.270344, -0.570031, 0.5503712, -1.30708, -0.6448291, 0.4530036, 1.577649, -1.352919, 0.09186998, -0.6309195, 0.3236146, -0.9113694, 0.5505443, 0.1130559, 0.3222194, 1.123799, 0.2195892, -0.1144144, -0.8139474, -0.9329815, -0.4625572, -0.8110472, -0.1414574, -0.9597951, 2.482504, 1.574074, 0.949287, -0.4212226, -0.9026161, 0.4742163, -1.030656, 0.7454542, 0.06286792, -0.6799075, 0.4078687, 1.474614, 0.368981, 1.048032, -0.3141561, 1.890041, -1.523312, 0.5052135, -0.03063537, -0.1812723, 0.5906073, -3.121271, 0.2007751, 0.7530114, -1.658598, 0.9336835, -0.4650073, -1.462019, 2.235685, 1.298409, 0.7269946, -1.471991, -0.8342447, 1.360999, -0.3951121, -0.2074053, -1.755657, -1.510649, 1.310844, 0.1556859, 1.006973, 0.100858, -1.757834, -1.533148, -0.1111837, -0.06857662, -0.1353461, 0.730463, 0.5255219, -0.6424738, 0.2418791, -0.8880968, 0.1806138, 0.2770524, -0.4064886, 2.329089, 1.336485, -0.7007003, 1.396216, 0.2506808, 0.8485276, 0.6959113, -2.987235, -0.248572, 0.7245918, -0.4089327, 0.9429503, 2.96197, 0.03184167, 0.03383717, -2.392359, 0.4891286, -0.7418132, 0.1317313, -0.7976542, 1.10234, 1.193196, -0.7249441, -0.8018743, 0.1443767, -0.1382338, 0.8005982, -1.726581, 1.337633, -1.657656, 0.8525519, -0.7505956, -0.5276248, 0.05544931, -0.004678294, 0.8143502, 0.3395111, -0.8701066, -0.07185977, 1.625616, -0.04410675, -0.1469374, 0.3847093, 1.529549, 0.1333284, 0.9479964, 0.7454173, -1.403134, -1.504152, -0.5172075, 0.4418704, 1.328946, -0.7284403, -0.6698911, 1.598608, 2.045127, -0.5353709, -0.2927103, 0.8229469, 0.8496788, -0.7278082, -0.5545287, -0.0336518, 0.6496707, 1.54537, 0.6041043, 2.457242, 0.3781433, 1.634661, 0.2058815, 0.7917272, -0.7259298, 0.8143947, -0.2537511, -1.2359, -1.179863, -0.576451, -1.362019, 0.6323203, 0.4560461, -1.469038, 0.3344935, 0.9665501, -0.7784179, -0.2783502, 1.23888, -0.1212549, -0.1492155, -0.3223093, 0.6283661, -0.589878, -0.0456115, 0.898512, 1.204413, 0.4762317, 1.266752, -2.466457, -1.242885, -0.2266665, -0.1127159, 0.4691368, 0.03494407, 1.112898, 0.8375851, 0.2529229, -0.954601, 1.462298, -1.000291, 1.278611, 0.5840442, 1.165406, 0.4717554, -1.067797, -0.1363591, -1.414558, -0.6298658, -0.2937561, -1.042784, 0.6779249, -1.191607, -0.122519, 0.3295524, -0.3402975, 1.33278, 0.4659011, 0.5081396, 1.328699, -0.4799021, 1.777418, 1.952968, -0.3489249, 0.1435661, 2.256666, 1.045201, 1.075223, -1.493977, 0.9462041, 0.4396593, -0.1842622, 1.256998, 0.4537135, -0.670919, 0.9618929, 0.3755627, -0.0586726, -1.066979, -0.7027138, -0.05473841, 0.4856082, -2.734774, -0.9911917, 0.7850441, -0.8738879, 0.7973333, 2.739657, 1.3148, -0.7145026, 0.6143026, 2.017837, 0.6412208, 0.8244266, -0.4546372, -0.9904545, -1.942163, -1.413132, 0.03171354, -0.8139976, -2.137059, -0.01020876, -0.4141979, 0.7488522, -0.6141782, 1.507088, -1.672687, 0.03504654, -0.7272652, -0.837869, 1.206503, 0.3045396, 0.5942119, 0.2013439, 0.8324106, 1.590089, -0.07985655, -0.1458268, 0.9537931, 0.4128874, 0.2991811, 1.068897, -0.01593985, 1.154212, 0.9795956, -0.8929546, 0.1127118, -0.04905733, 0.7304665, -1.177795, -0.5059839, -0.4622379, -0.4323988, -0.1305146, -1.08445, -0.2631727, -0.7506539, 0.8370473, -0.3303029, -1.207621, 0.3978009, -0.6451311, -0.7112153, 0.9179799, 1.622818, 1.427021, 0.01959129, -0.7179959, -0.4635325, -0.09525004, 0.2336147, 2.033225, -0.6779065, 0.9693504, 1.313946, -0.5083426, 0.530301, -1.719763, -0.02918128, 0.02455378, -1.952822, 0.4205124, -0.6731217, -0.4214074, 0.9907362, -0.3642928, -1.442634, -1.698527, -1.402633, 2.61315, -1.235118, -1.424661, 0.07877874, 0.4656505, 0.6088052, 0.253844, -1.356584, -0.183693, 0.5529984, 0.2354166, 1.158487, 0.2837288, 0.3977226, 0.4318069, 0.1717401, -0.5358616, -0.1918126, -0.9636851, 1.866944, -0.1161839, -1.15118, -0.9427516, -0.774172, -0.9683697, -0.4677881, -0.5619965, 0.9669993, -1.01076, 0.9677016, -1.07556, -0.7235349, 0.6522312, -0.889422, 0.3244094, -0.9926882, -0.18261, -1.705443, -1.756654, 1.363838, 0.4685941, 0.5468064, -1.343228, -1.170541, -1.164278, -1.215251, 1.294172, -1.14603, 1.037274, 1.714062, -0.6574762, -0.2790777, -0.845417, 0.473552, -1.994673, -0.1186627, -0.1346078, 1.126195, -0.5076234, -2.218447, -0.1402415, -1.728261, 0.7127917, 0.491851, -0.4789024, 1.481255, 0.07666979, -0.6280361, -0.1591145, 0.7737696, 0.5131709, 1.487569, -0.7952057, -0.7613349, -1.725068, -0.4910341, 1.171656, -0.003535699, -0.2401443, 2.833901, -2.071451, 1.70019, 0.7038061, -0.4181606, -1.404733, 0.4426538, 0.7964918, -0.5818622, 0.1461364, -2.253607, -0.2356653, 0.7249878, -0.3913796, -0.7004883, -0.4412545, -0.3456742, -1.004739, 2.350605, -0.2725976, -0.5316285, -2.118796, 0.9608248, -1.146196, 1.557605, -1.661092, -1.083196, -1.080946, -0.5511399, -1.074823, 0.8643694, 0.7397021, 0.1190481, -0.4670363, 0.4055022, -0.2492683, 0.5066869, -0.2100623, 0.4877414, -1.377375, 2.182191, -1.523079, -1.994307, 1.242817, 0.3662506, 1.082458, -2.365951, 0.3160132, 0.6425694, 0.7482273, -0.2495454, 1.086804, -1.47301, 0.8967451, -0.4332789, 0.06148769, -1.047484, -0.8734985, -1.157814, -1.051964, -0.04036733, -0.8232843, -0.05990444, -0.313392, -0.7706553, 0.7407132, 1.465334, -1.148255, -0.09572636, 0.9665478, 2.108467, -0.3339657, 0.6045657, -0.09284189, 0.1567415, -0.6233129, -1.443489, 1.21628, 0.7197523, 1.132003, -0.2526335, -1.548734, 0.7056614, 0.3994308, -0.2729337, 1.160706, 1.499382, 0.06861384, -0.4691241, -0.6535419, -1.339055, -0.258593, 0.1287391, -0.06465758, 0.1113027, 0.9602015, -1.718157, 0.2114756, -1.242299, -0.8658954, 0.2265295, 1.265814, 1.137428, 0.680548, -0.1146248, 0.6272912, 1.558373, 1.797743, 0.468379, -1.205372, 1.239773, 0.8029987, 0.7194438, -0.9033602, -1.606568, -0.4225149, 0.320648, -1.968752, -0.5057577, 0.3513472, -1.018402, -0.4476382, -0.07561492, -2.417428, 0.357013, -0.5273412, 0.535155, -0.3742867, 0.6497054, 1.580892, 0.6427628, -0.7036373, 0.2138398, 1.065993, -0.3490374, 1.586427, 0.2338042, 0.7711334, 1.740468, -0.6710801, 1.782727, 0.7121023, -1.183374, 1.064276, -1.695126, -0.2557385, 1.084438, -1.033578, 0.3141361, -0.9898091, -1.749729, -0.798597, 0.1490704, 2.23765, 0.2637828, 0.1453707, -0.04522033, -0.5075411, 0.3174352, -0.8230605, 1.015454, -0.01264923, -0.7928892, 0.07596593, 1.31203, -0.4897025, -0.1421514, 0.9896246, 0.6661193, 0.2344822, -0.6292128, -0.756409, 0.4040022, -0.4656512, -0.727107, -2.120602, -0.6552732, 1.433691, -1.445645, -1.513317, 0.07238387, -0.9635762, 1.031878, -0.0807357, 0.1064419, 1.047048, 1.456908, -0.805635, 1.015547, 1.056404, 0.1695087, 2.189564, -1.909823, 0.4568543, 0.4544218, -0.2096834, 0.5164022, -0.5706892, -1.225019, 0.5360669, 0.09647246, -1.090865, -0.2540544, -0.8054039, 0.6203786, -0.008791115, 0.117899, 1.796581, -1.872101, 0.13701, 0.2097139, -1.870792, 0.6623708, -0.06896637, -0.945677, -0.7705362, -1.509929, -0.005022428, 0.3228592, 0.4778416, -0.2186944, -0.595186, 0.3222623, 0.3482313, -0.3071618, -0.4595305, 0.6423125, -0.4690747, -1.752882, -1.379413, -0.01212552, 0.3902972, 0.7560513, -0.5660916, -0.2698721, 0.4533189, 0.799166, -0.5684291, 0.4093116, -0.9793326, -0.1590964, -0.6618587, -0.6973736, 0.7552651, -0.2923501, -0.101449, 0.07985463, -0.333099, 0.6388357, -0.3686107, -1.128198, 0.8527766, -0.05808835, -0.2614065, 0.5784443, -0.2705435, -0.5306369, 0.06652519, 0.1354608, -0.3815814, 0.8839219, 2.717485, 1.79515, 0.4734002, -1.138634, -0.4310051, 1.383635, -1.780314, -0.757142, 1.030547, 1.027722, -0.2636767, -0.8287573, 0.3980142, 0.3615886, -1.906882, -1.146163, 1.040275, 0.1453398, 0.1091221, -2.090463, -0.2469429, -0.9505968, -0.6768735, 1.04206, 1.097725, 0.5227192, 0.9352021, 0.3548014, -1.913862, 0.2352292, -1.224717, 0.1165188, -0.6604012, 1.024439, -0.9048131, -0.05033326, 0.06670278, 0.09185714, -0.1663515, -2.090608, 0.3794374, 1.037357, -0.4691749, 0.6840477, -0.04661748, -0.8881475, 0.4184137, 0.2720396, 0.6894528, -0.9946459, -0.04782868, 2.368767, 2.169457, -3.261632, 1.707746, -2.0399, 0.07674637, -0.2864999, -1.023442, -2.101809, -1.887759, 0.6246228, 0.7685984, -2.091363, 0.1310026, -2.766369, 1.032194, -0.8743405, 1.275031, -0.0184739, 0.7026404, 1.728222, 0.8195225, -0.447591, -0.3287751, 0.7547457, -0.1541807, 0.2658986, 0.3446286, -0.2905111, -0.3184831, 1.620896, 1.373413, 1.632094, 0.01310539, 0.06113945, -0.2276173, 1.211462, -0.5739729, 0.8032242, -0.5694138, 0.44919, -0.4648346, -0.7288542, -1.217562, 0.2146502, 0.06582939, 1.443551, 0.9444352, 1.33335)

##Estimation
#install.packages("R2jags")
library(R2jags)

model210001x<-function(){
	##Priors##
	beta~dnorm(0, 0.001) #use this option in a model with external variable
	phi.star1<-0 #use this option in a model without a first order seasonal autoregressive parameter
	r.theta.star1~dbeta(1,1)
	theta.star1<-2*r.theta.star1-1
	r~dgamma(0.01,0.01)
	## priors for non seasonal phi's##
	phi[1] <-y.phi[1,p] #use this option if phi[1] is included
	phi[2] <-y.phi[2,p] #use this option if phi[2] is included
	
	for (k in 1:p){
		alpha.phi[k] <-round(0.5*(k+1)-0.01)
		beta.phi[k] <-round((0.5*k)+1-0.01)
		r.phi[k] ~dbeta(alpha.phi[k], beta.phi[k])
		r.phi.map[k] <-2*r.phi[k]-1
	}
	y.phi[1,1]<-r.phi.map[1]
	for (k in 2:p){
		for (i in 1:(k-1)){
		y.phi[i,k] <-y.phi[i,k-1]-r.phi.map[k]*y.phi[k-i,k-1]
		}
		y.phi[k,k] <-r.phi.map[k]
	}
	##
	##Deviance##
	Dev <- -2*sum(LL[(w+1) : N])
	##
	##Error for first w observations##
	for (t in 1: w){
		u[t] <- 0
	}
	##
	##Likelihood ##
	for (t in (w+1):N){
		y[t]~dnegbin(pr[t],r)
		y.p1[t]~dnegbin(pr[t],r)
		pr[t]<-r/(r+lambda[t])
		lambda[t]<-exp(m[t])
		m[t] <- beta * x[t] 
+log(max(c,y[t-1])) 
-beta * x[t-1] 
+ sum(AR[ ,t]) +(theta.star1)*u[t-12]
		for (k in 1:p){
			AR[k,t]<-
 (phi[k])*log(max(c,y[t-k]))
-phi[k] * beta * x[t-k] 
-(phi[k])*log(max(c,y[t-k-1]))
+phi[k] * beta * x[t-k-1]
		}
		u[t] <- log(max(c,y[t])/lambda[t])
		LL[t] <-r*log(pr[t])+y[t]*log(1-pr[t])+loggam(y[t]+r)- loggam(y[t]+1) - loggam(r)
	}
}
write.model(model210001x, con = "model210001x.txt")

N<-1000
w<-12
c<-1
p<-2
inits<- list(list(r=2, r.theta.star1=0.2, beta=1), list(r=4, r.theta.star1=0.4, beta=0), list(r=8, r.theta.star1=0.8, beta=-1))
parameters<-c("r", "phi", "theta.star1", "Dev", "beta", paste("y.p1[",w+1,":",N,"]",sep="")) 
data<-list(N=N, w=w, p=p, c=c, y= y[1:N], x=x)

##estimation:
ptm <- proc.time()
jags.output.model210001x <- jags(data= data, inits, parameters, model.file= "model210101x.txt",
    n.iter=2000, n.burnin=1000, n.chains=3, n.thin=1)
proc.time() - ptm
alarm()

print(jags.output.model210001x, digits=2)
#Inference for Bugs model at "model210001x.txt", fit using jags,
# 3 chains, each with 2000 iterations (first 1000 discarded)
# n.sims = 3000 iterations saved
#       mu.vect  sd.vect     2.5%      25%       50%       75%     97.5% Rhat n.eff
#Dev  9.986e+03     3.21  9982.72  9984.58 9.986e+03 9.988e+03 9.994e+03 1.00  3000
#beta 7.000e-01     0.01     0.68     0.69 7.000e-01 7.000e-01 7.100e-01 1.00  1200
#phi[1] 4.7e-01     0.03     0.41     0.45 4.700e-01 4.900e-01 5.300e-01 1.00  3000
#phi[2] 1.7e-01     0.03     0.11     0.15 1.700e-01 1.900e-01 2.300e-01 1.00  3000
#r    5.040e+00     0.29     4.49     4.83 5.030e+00 5.230e+00 5.640e+00 1.00  1300
#theta.star1 5e-01  0.02     0.46     0.49 5.000e-01 5.200e-01 5.500e-01 1.00  3000
#deviance 9.986e+03 3.21  9982.72  9984.58 9.986e+03 9.988e+03 9.994e+03 1.00  3000

#For each parameter, n.eff is a crude measure of effective sample size,
and Rhat is the potential scale reduction factor (at convergence, Rhat=1).

#DIC info (using the rule, pD = var(deviance)/2)
#pD = 5.2 and DIC = 9992.1
#DIC is an estimate of expected predictive error (lower deviance is better).
res.mcmc<-as.mcmc(jags.output.model210001x)
res.mcmc.sel<-res.mcmc[][,1:4]
res.list<-mcmc.list(res.mcmc.sel[[1]],res.mcmc.sel[[2]],res.mcmc.sel[[2]])
gelman.diag(res.list)
gelman.plot(res.list)
re<-(jags.output.model210001x$BUGSoutput$median$y.p1[1:(N-w)]-y[(w+1):N])/ (y[(w+1):N]+1)
mean(abs(re))
#[1] 0.4461987


##Example of GSARIMA(0,1,2)x(1,0,0)-x
theta<-c(0.6,0.2)
phi<-c(0)
Phi<-c(0.4)
Theta<-c(0)
d<-1
D<-0
frequency<-12
ar<-arrep(phi=phi, theta=theta, Phi=Phi, Theta=Theta, frequency= frequency, d=d, D=D)
N<-1000
intercept<-10
theta<-3
x<- x ##(use previous x)
beta.x<- -0.3
X<-matrix(c(rep(log(intercept), N+length(ar)), rep(0, length(ar)), x), ncol=2)
c<-1
y.sim <- garsim(n=(N+length(ar)), phi=ar, beta=c(1,beta.x), link= "log", family= "negative.binomial", zero.correction = "zq1", c=c, theta=theta, X=X) 
y<-y.sim[(1+length(ar)):(N+length(ar))]
tsy<-ts(y, freq=frequency)
plot(tsy)
plot(log(tsy))

#cat(y, sep=", ")
#y<-c(12, 3, 3, 2, 7, 10, 30, 68, 63, 5, 1, 0, 0, 0, 0, 0, 3, 3, 6, 9, 29, 10, 9, 0, 2, 0, 7, 2, 8, 10, 4, 0, 4, 2, 1, 1, 3, 2, 2, 2, 2, 5, 7, 5, 7, 52, 13, 1, 0, 1, 2, 1, 0, 4, 4, 3, 0, 0, 0, 1, 1, 0, 0, 1, 0, 4, 5, 4, 7, 6, 4, 0, 3, 18, 25, 87, 81, 57, 10, 8, 17, 5, 0, 0, 0, 2, 0, 2, 1, 1, 0, 10, 26, 7, 5, 18, 19, 18, 8, 10, 7, 3, 4, 14, 10, 1, 0, 1, 1, 1, 1, 0, 1, 1, 0, 0, 1, 1, 2, 9, 4, 1, 0, 4, 9, 8, 3, 6, 9, 21, 20, 52, 24, 13, 7, 8, 10, 23, 10, 1, 1, 0, 11, 26, 23, 48, 12, 24, 23, 86, 44, 10, 3, 1, 0, 3, 4, 2, 2, 4, 3, 10, 2, 0, 0, 4, 11, 22, 33, 56, 66, 49, 16, 17, 22, 9, 24, 30, 82, 46, 36, 24, 42, 13, 11, 6, 0, 0, 11, 91, 259, 186, 101, 23, 14, 2, 0, 2, 4, 4, 12, 25, 23, 5, 2, 1, 1, 1, 5, 6, 2, 0, 4, 4, 5, 3, 7, 7, 5, 3, 4, 2, 0, 1, 3, 0, 1, 0, 3, 3, 16, 14, 25, 1, 0, 1, 1, 2, 1, 5, 1, 0, 0, 2, 5, 5, 6, 13, 26, 11, 5, 2, 4, 1, 0, 3, 3, 0, 0, 2, 4, 4, 2, 1, 0, 0, 0, 0, 0, 0, 3, 5, 7, 8, 3, 0, 2, 6, 10, 1, 0, 1, 1, 2, 1, 2, 2, 3, 5, 3, 12, 0, 0, 0, 0, 6, 27, 13, 35, 21, 19, 6, 0, 2, 9, 30, 50, 35, 66, 17, 30, 43, 28, 4, 0, 1, 4, 26, 131, 56, 62, 27, 21, 12, 4, 1, 0, 0, 2, 2, 1, 0, 0, 2, 2, 0, 1, 3, 2, 5, 2, 9, 14, 10, 1, 0, 0, 2, 2, 2, 4, 23, 4, 4, 2, 0, 1, 2, 4, 6, 6, 7, 9, 19, 32, 30, 17, 4, 1, 0, 2, 10, 20, 50, 131, 86, 123, 123, 16, 5, 1, 0, 0, 3, 10, 13, 12, 1, 0, 0, 0, 2, 1, 9, 40, 115, 112, 219, 257, 57, 56, 104, 110, 212, 611, 1059, 473, 335, 156, 58, 101, 38, 21, 32, 166, 142, 70, 75, 95, 29, 18, 9, 7, 2, 0, 0, 1, 5, 12, 42, 26, 54, 16, 22, 6, 2, 0, 2, 1, 0, 0, 0, 1, 0, 0, 1, 2, 0, 1, 1, 0, 1, 1, 3, 23, 27, 30, 50, 80, 194, 73, 14, 1, 0, 0, 0, 0, 1, 1, 2, 1, 0, 0, 3, 3, 9, 46, 25, 16, 28, 21, 12, 0, 1, 2, 10, 32, 131, 150, 50, 25, 17, 1, 0, 2, 2, 0, 0, 0, 3, 4, 2, 2, 6, 1, 0, 0, 2, 1, 1, 2, 12, 15, 33, 25, 53, 43, 27, 16, 7, 11, 6, 8, 6, 9, 44, 144, 235, 31, 8, 3, 0, 0, 1, 1, 0, 0, 1, 2, 2, 6, 9, 5, 7, 3, 2, 5, 4, 2, 1, 0, 1, 0, 5, 11, 11, 19, 38, 106, 59, 6, 31, 41, 25, 15, 11, 20, 48, 11, 2, 5, 5, 4, 4, 5, 3, 2, 1, 0, 0, 7, 3, 2, 0, 1, 2, 5, 8, 14, 5, 10, 17, 44, 126, 110, 81, 41, 102, 45, 17, 17, 8, 9, 1, 1, 0, 3, 17, 14, 13, 18, 5, 3, 0, 1, 0, 2, 1, 0, 1, 0, 6, 11, 16, 21, 21, 52, 105, 87, 26, 9, 4, 21, 23, 25, 23, 25, 16, 4, 2, 10, 0, 0, 2, 1, 0, 0, 5, 4, 2, 0, 1, 0, 1, 0, 1, 0, 1, 3, 5, 7, 3, 18, 9, 15, 18, 17, 7, 0, 0, 1, 0, 1, 1, 4, 9, 12, 1, 0, 1, 1, 3, 1, 5, 10, 18, 5, 6, 3, 1, 0, 6, 4, 15, 9, 5, 1, 1, 4, 10, 22, 8, 18, 67, 27, 18, 21, 13, 5, 1, 6, 24, 73, 65, 71, 53, 17, 63, 47, 49, 50, 21, 33, 72, 47, 79, 253, 401, 297, 413, 365, 277, 136, 87, 193, 658, 189, 114, 47, 100, 148, 269, 673, 2578, 1629, 2193, 6040, 18390, 36087, 7173, 8218, 7420, 7829, 7778, 2763, 750, 871, 435, 306, 749, 422, 71, 50, 1, 0, 2, 4, 1, 1, 0, 3, 5, 1, 2, 2, 0, 0, 1, 2, 0, 1, 2, 0, 2, 0, 0, 3, 1, 1, 2, 3, 4, 1, 0, 0, 1, 0, 4, 31, 37, 70, 152, 206, 135, 152, 137, 45, 8, 5, 10, 15, 9, 9, 15, 35, 9, 7, 15, 18, 12, 10, 3, 7, 4, 1, 0, 4, 2, 1, 0, 0, 6, 1, 4, 31, 21, 19, 22, 16, 18, 19, 8, 10, 2, 1, 2, 2, 0, 1, 2, 4, 1, 1, 0, 1, 0, 0, 0, 0, 0, 4, 6, 9, 4, 5, 2, 4, 11, 16, 2, 1, 2, 13, 24, 26, 14, 33, 6, 3, 0, 0, 0, 0, 3, 6, 6, 2, 1, 0, 0, 0, 2, 2, 1, 5, 8, 11, 11, 8, 1, 0, 4, 13, 7, 3, 2, 8, 10, 11, 8, 3, 1, 1, 1, 4, 0, 0, 1, 1, 0, 0, 1, 0, 0, 2, 1, 1, 1, 4, 5, 8, 4, 6, 2, 2, 1, 0, 3, 2, 1, 1, 4, 8, 2, 0, 0, 0, 0, 1, 6, 7, 1, 0, 6, 1, 0, 0, 2, 2, 1, 0, 1, 7, 14, 7, 13, 1, 0, 1, 3, 2, 2, 3, 11, 4, 0, 1, 0, 0, 1, 3, 1, 1, 2, 0, 0, 0, 0, 3, 3, 2, 0, 0, 0, 0, 0, 0, 1, 2, 8) 

##Estimation
model012100x<-function(){
	##Priors
	beta~dnorm(0,0.001)
	r~dgamma(0.01,0.01)
	phi[1]<-0
	r.phi.star~dbeta(1,1)
	phi.star<-2*r.phi.star-1
	##priors for non seasonal theta's
	theta[1] <-y.theta[1,q] #use this option if phi[1] is included
	theta[2] <-y.theta[2,q] #use this option if phi[2] is included
	for (k in 1:q){
		alpha.theta[k] <-round(0.5*(k+1)-0.01)
		beta.theta[k] <-round((0.5*k)+1-0.01)
		r.theta[k] ~dbeta(alpha.theta[k], beta.theta[k])
		r.theta.map[k] <-2*r.theta[k]-1
	}
	y.theta[1,1]<-r.theta.map[1]
	for (k in 2:q){
		for (i in 1:(k-1)){
		y.theta[i,k] <-y.theta[i,k-1]-r.theta.map[k]*y.theta[k-i,k-1]
		}
		y.theta[k,k] <-r.theta.map[k]
	}
	
	for (t in 1: (1+12)){
		u[t] <- 0
	}
##likelihood
	for (t in (1+12+1):N){
		y[t]~dnegbin(pr[t],r)
		y.p1[t]~dnegbin(pr[t],r)
		pr[t]<-r/(r+lambda[t])
		lambda[t]<-exp(m[t])
		m[t] <- beta * x[t] 
+log(max(c,y[t-1])) 
-beta * x[t-1] 
+ SAR[t] + theta[1]*u[t-1] +theta[2]*u[t-2]
		SAR[t]<-
 (phi.star)*log(max(c,y[t-12])) 
-phi.star * beta * x[t-12]
-(phi.star)*log(max(c,y[t-1-12]))
+ phi.star * beta * x[t-1-12]
		u[t] <- log(max(c,y[t])/lambda[t])
	}
}
write.model(model012100x, con ="model012100x.txt")

inits<- list(list(r=2, r.phi.star=0.2, beta=1), list(r=4, r.phi.star=0.4, beta=0), list(r=8, r.phi.star=0.8, beta=-1))
q<-2
w<-13
c<-1
data<-list( N=N, q=q,  c=c, y= y[1:N], x=x[1:N])
parameters<-c("r", "theta", "phi.star",  "beta", paste("y.p1[",w+1,":",N,"]",sep="")) 
#estimation:
ptm <- proc.time()
jags.output.model012100x<- jags(data= data, inits, parameters, model.file= "model012100x.txt",
    n.iter=2000, n.burnin=1000, n.thin=1, n.chains=3)
alarm()
proc.time() - ptm

print(jags.output.model012100x, digits=2)

#Inference for Bugs model at "model012100x.txt", fit using jags,
# 3 chains, each with 2000 iterations (first 1000 discarded)
# n.sims = 3000 iterations saved
#           mu.vect  sd.vect    2.5%      25%      50%      75%    97.5% Rhat n.eff
#beta         -0.32     0.01   -0.34    -0.32    -0.32    -0.31    -0.29 1.00  1600
#phi.star      0.38     0.03    0.32     0.36     0.38     0.40     0.44 1.00  3000
#r             3.30     0.24    2.85     3.13     3.29     3.46     3.77 1.00  3000
#theta[1]      0.60     0.03    0.53     0.57     0.60     0.62     0.66 1.00   930
#theta[2]      0.22     0.03    0.16     0.20     0.22     0.24     0.28 1.00   540
#y.p1[14]      0.44     0.71    0.00     0.00     0.00     1.00     2.00 1.00  1900
#For each parameter, n.eff is a crude measure of effective sample size,
#and Rhat is the potential scale reduction factor (at convergence, Rhat=1).
#
#DIC info (using the rule, pD = var(deviance)/2)
#pD = 5.3 and DIC = 5525.2
#DIC is an estimate of expected predictive error (lower deviance is better).

res.mcmc<-as.mcmc(jags.output.model012100x)
res.mcmc.sel<-res.mcmc[][,1:5]
res.list<-mcmc.list(res.mcmc.sel[[1]],res.mcmc.sel[[2]],res.mcmc.sel[[2]])
gelman.diag(res.list)
gelman.plot(res.list)
re<-(jags.output.model012100Jxnodsum$BUGSoutput$median$y.p1[1:(N-w)]-y[(w+1):N])/ (y[(w+1):N]+1)
mean(abs(re))
#[1] 0.5461414
