# Supplementary material for: Generalized Seasonal Autoregressive Integrated Moving Average Models for Count Data with Application to Malaria Time Series with Low Case Numbers
Source: PLoS One. 2013 Jun 13;8(6):e65761. doi: 10.1371/journal.pone.0065761 (PMC3681978; doi:10.1371/journal.pone.0065761)
Supplement: Additional File S2 — R code for the analysis of monthly malaria case count data for the district of Gampaha, Sri Lanka. (RTF) [file pone.0065761.s006.rtf]

Additional file S2
##Additional File S2, supporting information to Briët et al.: “Generalized seasonal autoregressive integrated moving average models for count data with application to malaria time series with low case numbers”
##This file contains computer code for use in the free software R [http://cran.r-project.org/]. For the code to run, it requires a few R packages, and JAGS [http://mcmc-jags.sourceforge.net/] to be installed. It describes the analysis of monthly malaria case count data for the district of Gampaha, Sri Lanka. 

##Data
##Monthly malaria cases in Gampaha, Sri Lanka, from January 1972
y <- c(62,59,70,42,85,52,97,98,85,54,92,134,101,59,46,49,56,82,106,178,140,162,204,390,442,407,607,374,254,275,283,223,145,186,166,221,279,221,170,105,141,296,298,226,169,239,177,145,141,155,110,178,191,573,2462,915,1005,507,530,279,965,668,818,463,392,297,379,508,333,393,401,213,206,90,158,110,125,132,86,70,105,183,152,72,97,103,171,107,251,239,401,149,151,134,96,82,73,87,91,64,62,51,48,63,64,35,38,57,37,26,25,35,30,35,44,20,12,3,8,6,1,9,9,26,63,39,10,3,5,0,13,29,35,40,79,187,1402,2507,2008,240,476,711,653,710,603,826,770,625,390,326,279,301,231,155,137,170,132,94,107,51,46,68,95,89,104,103,127,149,126,132,123,72,134,159,194,335,262,389,186,408,751,703,973,1287,1864,1771,1536,923,1778,1039,895,496,657,317,395,298,287,262,202,171,134,103,138,92,139,80,66,124,399,489,188,190,117,85,113,99,156,59,253,452,405,366,196,193,111,204,381,721,1506,580,315,224,164,212,176,211,223,268,167,264,530,333,237,194,631,989,684,439,342,444,512,515,675,425,451,424,467,393,295,326,350,377,473,638,703,543,515,356,323,404,397,380,379,404,310,305,352,286,298,149,154,130,111,87,105,121,150,140,178,201,207,215,206,543,1163,724,339,246,197,199,244,313,239,247,223,352,406,248,228,119,87,103,95,86,113,105,97,108,224,251,213,111,127,98,104,99,82,55,87,74,82,70,76,133,141,204,208,148,95,57,90,149,197,310,282,150,100,63,84,55,38,28,29,27,31,24,19,24,34,32,30,25,17,16,14,21,20,11,12,8,11,7,10,8,5,4,6,5,2,5,3,2,2,1,2,1,2,3,1,1,15,4,4,2,1,1,2,2,0,1,3,20,19,0,2,1,4,2,5,1,0,0,1,2,1,0,0,1,0,0)
length(y)
#[1] 420

##Monthly rainfall in Gampaha, Sri Lanka, from 1971, November
x <- c(53,203,13,2,129,218,529,145,111,83,294,522,345,192,15,77,246,313,316,290,180,106,55,363,345,285,2,151,161,501,383,260,272,121,404,118,132,202,31,100,173,357,436,301,180,154,319,279,573,141,12,7,147,268,175,98,142,163,85,402,415,220,13,109,271,228,895,242,32,155,130,691,304,57,14,61,231,177,572,208,40,42,117,302,513,118,17,156,117,161,129,395,108,37,411,344,422,215,1,1,97,299,221,306,128,181,243,326,314,138,125,62,90,162,346,247,79,138,266,301,393,89,6,1,167,188,353,431,226,265,114,378,534,104,5,6,6,89,303,216,128,168,378,135,264,258,249,209,266,460,483,241,252,17,175,230,337,54,72,192,181,140,365,442,62,161,152,391,337,200,138,128,119,243,211,100,30,145,249,308,174,133,34,1,46,262,191,300,15,308,304,643,238,64,3,191,135,309,206,326,138,263,393,98,347,65,32,2,89,255,221,444,264,93,221,302,304,77,126,14,178,153,288,134,183,3,25,364,564,168,105,21,218,145,377,409,145,68,93,358,274,93,11,1,1,158,441,240,234,110,357,387,343,92,9,27,108,209,366,127,95,66,319,527,399,270,76,119,100,196,368,89,118,74,264,554,374,9,132,29,62,527,466,380,116,145,118,322,450,25,86,105,23,294,97,137,152,214,414,243,229,130,5,42,41,211,311,145,264,81,423,559,481,220,35,5,71,206,298,257,374,217,274,382,275,335,90,131,69,421,468,163,168,126,193,746,359,92,225,187,142,201,186,163,49,267,479,260,180,122,171,121,12,404,188,137,70,12,308,240,216,117,47,77,129,503,350,182,45,63,79,551,288,205,158,84,271,269,256,244,254,137,144,306,251,44,17,40,77,211,311,267,223,93,428,412,342,145,97,49,190,233,222,207,141,42,129,533,628,177)
length(x)
#[1] 410

tsy<-ts(y, start=c(1972,1), freq=12)
win.tsy<-window(tsy, end=c(2005,12))
length(win.tsy)
#[1] 408


tsx<-ts(x, start=c(1971,11), freq=12)
win.tsx<-window(tsx,start=c(1971,11), end=c(2005,12))
length(win.tsx)


N<-length(win.tsy)

tiff(filename = "Figure1.tif",
     width = 8.3, height = 16.6, units = "cm", pointsize = 8,
     compression = c("lzw"),
     bg = "white", res = 1000, family = "Arial", restoreConsole = TRUE,
     type = c("cairo"), antialias=c("subpixel"))
op <- par(mar=c(2,3.6,0,0),omi=c(0.205, 0.02778, 0.02778, 0.02778), mgp=c(2.5,1,0), cex.lab=1.4, mfrow=c(2,1))
plot(y[1:408], ylab= "Malaria cases", xaxt="n", type ="n", xlab="") ##Figure 1 in main article
abline(h=0, lty=1, col=8)
lines(y[1:408])
text(20, 2400, "A")

axis(1, at=c(0,1,2,3,4,5,6)*12*5+36, labels=FALSE)
bp<-barplot(x[3:410], ylab= "Rainfall (mm)", col=4, border=NA, width=1, space=c(0,0), xaxt="n", main=NULL, ylim=c(0,1000)) ##Figure 1 in main article
#bp
axis(1, at=c(0,1,2,3,4,5,6)*12*5+36, labels=c(1975,1980,1985, 1990, 1995, 2000, 2005))
box()
text(20, 900, "B")
mtext("Time", side=1, outer=FALSE, line=2.5, cex=1.3) 
par(op)
dev.off()


##Preliminary frequentist Gaussian SARIMA model identification
#install.packages("geoR")
library(geoR)
#bc.fit<-boxcox.fit(win.tsy, lambda2=TRUE) ##Prior to geoR VERSION 1.7-1
bc.fit<-boxcoxfit(win.tsy, lambda2=TRUE)

bc.fit
#Fitted parameters: boxcoxfit
#    lambda    lambda2       beta    sigmasq 
# 0.2491499  0.0250700  9.7501517 22.7459922 
#
#Convergence code returned by optim: 0
#install.packages("car")
library(car)
#win.tsy.bc <-box.cox(win.tsy, bc.fit$lambda[1], start= bc.fit$lambda[2]) ##Prior to car VERSION 2.0
win.tsy.bc <-bcPower(win.tsy+ bc.fit$lambda[2], bc.fit$lambda[1])

box.cox.bt<-function(x, lambda, start){
y<- (lambda*x+1)^(1/lambda) -start
}

win.tsy.bcbt <-box.cox.bt(win.tsy.bc, bc.fit$lambda[1], start= bc.fit$lambda[2])
plot(win.tsy, win.tsy.bcbt) ##Check for Box-Cox transformation and back transformation

qqnorm(win.tsy.bc, main=NULL)
qqline(win.tsy.bc) ##Visual check of transformation

tsy.bc <-ts(win.tsy.bc, start=c(1972,1), freq=12)
plot(tsy.bc, ylab= "Box-Cox transformed malaria cases") ##Figure S1


#install.packages("extrafont")
library(extrafont)
#font_import()
fonttable()
loadfonts()
pdf(file = "FigureS1.pdf",
     width = 6.83, height = 6.83, pointsize = 8,
     bg = "white", family = "Arial")
op <- par(mar=c(4,4,4,2),omi=c(0.02778, 0.02778, 0.02778, 0.02778), mgp=c(2.5,1,0), cex.lab=1.4)
plot(tsy.bc, ylab= "Box-Cox transformed malaria cases", main="Box-Cox tranformed monthly malaria case counts in Gampaha") 
par(op)
dev.off()

plot(diff(tsy.bc), ylab= "First order differenced Box-Cox transformed malaria cases")
#install.packages("tseries")
library(tseries)
adf.test(tsy.bc, k=15)
#        Augmented Dickey-Fuller Test
#
#data:  tsy.bc 
#Dickey-Fuller = -3.0428, Lag order = 15, p-value = 0.1369
#alternative hypothesis: stationary

##So this series contains a unit root

acf(diff(win.tsy.bc), lag.max=36, main= "") #Figure S2

#install.packages("extrafont")
library(extrafont)
#font_import()
#fonttable()
loadfonts()
pdf(file = "FigureS2.pdf",
     width = 6.83, height = 6.83, pointsize = 8,
     bg = "white", family = "Arial")
op <- par(mar=c(4,4,4,2),omi=c(0.02778, 0.02778, 0.02778, 0.02778), mgp=c(2.5,1,0), cex.lab=1.4)
acf(diff(win.tsy.bc), lag.max=36, main= "Autocorrelation function of Box-Cox transformed monthly malaria case counts in Gampaha") #Figure S2
par(op)
dev.off()


pacf(diff(win.tsy.bc), lag.max=36, main= "") #Figure S3

pdf(file = "FigureS3.pdf",
     width = 6.83, height = 6.83, pointsize = 8,
     bg = "white", family = "Arial")
op <- par(mar=c(4,4,4,2),omi=c(0.02778, 0.02778, 0.02778, 0.02778), mgp=c(2.5,1,0), cex.lab=1.4)
pacf(diff(win.tsy.bc), lag.max=36, main= "Partial autocorrelation function of Box-Cox transformed monthly malaria case counts in Gampaha") #Figure S2
par(op)
dev.off()

arima.fit<-arima(tsy.bc, order=c(3,1,0), seasonal=list(order=c(1,0,0)), fixed=c(0,0,NA,NA))
arima.fit
#Call:
#arima(x = tsy.bc, order = c(3, 1, 0), seasonal = list(order = c(1, 0, 0)), fixed = c(0, 
#    0, NA, NA))
#
#Coefficients:
#      ar1  ar2      ar3    sar1
#        0    0  -0.2205  0.0947
#s.e.    0    0   0.0484  0.0506
#
#sigma^2 estimated as 3.231:  log likelihood = -816.3,  aic = 1638.61
tsdiag(arima.fit)
bt<-Box.test(arima.fit$res, lag = 15, type = "Ljung-Box")
bt$statistic
#X-squared 
# 8.905198 
pchisq(q= bt$statistic[[1]], df=15-2, lower.tail=FALSE)
#[1] 0.7800652
qchisq(p=0.95, df=(15-2))
#[1] 22.36203
tsdiag(arima.fit)

arima.fit<-arima(tsy.bc, order=c(3,1,0), seasonal=list(order=c(0,0,1)), fixed=c(0,0,NA,NA))
arima.fit
#sigma^2 estimated as 3.234:  log likelihood = -816.48,  aic = 1638.95
tsdiag(arima.fit)

arima.fit<-arima(tsy.bc, order=c(0,1,3), seasonal=list(order=c(1,0,0)), fixed=c(0,0,NA,NA))
arima.fit
#sigma^2 estimated as 3.23:  log likelihood = -816.22,  aic = 1638.44
tsdiag(arima.fit)

arima.fit<-arima(tsy.bc, order=c(0,1,3), seasonal=list(order=c(0,0,1)), fixed=c(0,0,NA,NA))
arima.fit
#sigma^2 estimated as 3.233:  log likelihood = -816.4,  aic = 1638.79
tsdiag(arima.fit)

arima.fit<-arima(tsy.bc, order=c(3,1,0), seasonal=list(order=c(1,0,0)), xreg=x[1:408]/1000, fixed=c(0,0,NA,NA,NA))
arima.fit
#sigma^2 estimated as 3.229:  log likelihood = -816.17,  aic = 1640.35
tsdiag(arima.fit)

arima.fit<-arima(tsy.bc, order=c(3,1,0), seasonal=list(order=c(0,0,1)), xreg=x[1:408]/1000, fixed=c(0,0,NA,NA,NA))
arima.fit
#sigma^2 estimated as 3.232:  log likelihood = -816.37,  aic = 1640.74
tsdiag(arima.fit)

arima.fit<-arima(tsy.bc, order=c(0,1,3), seasonal=list(order=c(1,0,0)), xreg=x[1:408]/1000, fixed=c(0,0,NA,NA,NA))
arima.fit
#sigma^2 estimated as 3.229:  log likelihood = -816.18,  aic = 1640.36
tsdiag(arima.fit)

arima.fit<-arima(tsy.bc, order=c(0,1,3), seasonal=list(order=c(0,0,1)), xreg=x[1:408]/1000, fixed=c(0,0,NA,NA,NA))
arima.fit
#sigma^2 estimated as 3.232:  log likelihood = -816.37,  aic = 1640.74
tsdiag(arima.fit)

SOH<-matrix(nrow= length(tsy.bc), ncol=4)
for (t in 1:length(tsy.bc)){
	SOH[t,1]<-sin(2*pi*t/12)
	SOH[t,2]<- cos(2*pi*t/12)
	SOH[t,3]<- sin(2*pi*t/6)
	SOH[t,4]<- cos(2*pi*t/6)
}

arima.fit<-arima(tsy.bc, order=c(3,1,0), xreg=SOH,fixed=c(0,0,NA,NA,NA,NA,NA))
arima.fit
#sigma^2 estimated as 3.135:  log likelihood = -810.1,  aic = 1632.2
tsdiag(arima.fit)

rainSOH<-cbind(x[1:408]/1000,SOH)
arima.fit<-arima(tsy.bc, order=c(3,1,0), xreg=rainSOH, fixed=c(0,0,NA,NA,NA,NA,NA,NA))
arima.fit
#sigma^2 estimated as 3.108:  log likelihood = -808.34,  aic = 1630.68
tsdiag(arima.fit)

arima.fit<-arima(tsy.bc, order=c(0,1,3), xreg=SOH, fixed=c(0,0,NA,NA,NA,NA,NA))
arima.fit
#sigma^2 estimated as 3.128:  log likelihood = -809.63,  aic = 1631.27
tsdiag(arima.fit)

arima.fit<-arima(tsy.bc, order=c(0,1,3), xreg=rainSOH, fixed=c(0,0,NA,NA,NA,NA,NA,NA))
arima.fit
#sigma^2 estimated as 3.104:  log likelihood = -808.04,  aic = 1630.07
tsdiag(arima.fit)

SOHres<-matrix(nrow= 365, ncol=4)
for (t in (1:365)/1){
	SOHres[t*1,1]<-sin(2*pi*t/365)*arima.fit$coef[5]
	SOHres[t*1,2]<- cos(2*pi*t/365) *arima.fit$coef[6]
	SOHres[t*1,3]<- sin(2*pi*t/(365/2)) *arima.fit$coef[7]
	SOHres[t*1,4]<- cos(2*pi*t/(365/2)) *arima.fit$coef[8]
}
plot(rowSums(SOHres), type="l", ylab="Deterministic seasonal component", xlab="Days")

##Above results are summarized in Table 1 in the main article. Other models that were tried but not selected:

arima.fit<-arima(tsy.bc, order=c(2,1,0), seasonal=list(order=c(0,0,1)), fixed=c(0,NA,NA))
arima.fit

arima.fit<-arima(tsy.bc, order=c(4,1,0), seasonal=list(order=c(0,0,1)), fixed=c(0,0,NA,NA,NA))
arima.fit

arima.fit<-arima(tsy.bc, order=c(3,1,0), seasonal=list(order=c(0,0,1)), fixed=c(NA,NA,NA,NA))
arima.fit

arima.fit<-arima(tsy.bc, order=c(2,1,0), seasonal=list(order=c(1,0,0)), fixed=c(0,NA,NA))
arima.fit

arima.fit<-arima(tsy.bc, order=c(4,1,0), seasonal=list(order=c(1,0,0)), fixed=c(0,0,NA,NA,NA))
arima.fit

arima.fit<-arima(tsy.bc, order=c(3,1,0), seasonal=list(order=c(1,0,0)), fixed=c(NA,NA,NA,NA))
arima.fit

##Of the G(S)ARIMA models presented in Table 2 in the main article, only the code for the two selected models is presented:

#install.packages("R2jags")
library(R2jags)
model310SOHx<-function(){
	##Deviance
	Dev <- -2*sum(LL[(w+1) : N])
	##
	for (t in 1:w){
		x[1,t]<-rainlag2[t]/1000
		x[2,t]<-sin(2*pi*t/12)
		x[3,t]<- cos(2*pi*t/12)
		x[4,t]<- sin(2*pi*t/6)
		x[5,t]<- cos(2*pi*t/6)
	}
	for (t in (w+1):N){
		y[t]~dnegbin(pr[t],r)
		y.p1[t]~dnegbin(pr[t],r)
		pr[t]<-r/(r+lambda[t])
		lambda[t]<-exp(m[t])
		m[t]<- inprod(beta[],x[,t]) + log(max(c,y[t-1])) - 	inprod(beta[],x[,t-1]) + sum(AR[ ,t])
		for (k in 1:p){
			AR[k,t]<-
			 (phi[k])*log(max(c,y[t-k]))
			-phi[k] * inprod(beta[],x[,t-k])
			-(phi[k]) * log(max(c,y[t-k-1]))
			+phi[k] * inprod(beta[],x[,t-k-1])
		}
		x[1,t]<-rainlag2[t]/1000
		x[2,t]<-sin(2*pi*t/12)
		x[3,t]<- cos(2*pi*t/12)
		x[4,t]<- sin(2*pi*t/6)
		x[5,t]<- cos(2*pi*t/6)
		LL[t] <-r*log(pr[t])+y[t]*log(1-pr[t])+loggam(y[t]+ r)- loggam(y[t]+1) - loggam(r)
	}
	##Prediction out of sample (activate in case N<M) 
	#for (t in (N+1):M){
	#	y.p1[t]~dnegbin(pr[t],r)
	#	pr[t]<-r/(r+lambda[t])
	#	lambda[t]<-exp(m[t])
	#	m[t]<- inprod(beta[],x[,t]) + log(max(c,y[t-1])) - inprod(beta[],x[,t-1]) + sum(AR[ ,t])
	#	for (k in 1:p){
	#		AR[k,t]<-
	#		 (phi[k])*log(max(c,y[t-k]))
	#		-phi[k] * inprod(beta[],x[,t-k])
	#		-(phi[k]) * log(max(c,y[t-k-1]))
	#		+phi[k] * inprod(beta[],x[,t-k-1])
	#	}
	#	x[1,t]<-rainlag2[t]/1000
	#	x[2,t]<-sin(2*pi*t/12)
	#	x[3,t]<- cos(2*pi*t/12)
	#	x[4,t]<- sin(2*pi*t/6)
	#	x[5,t]<- cos(2*pi*t/6)
	#	LL[t] <-r*log(pr[t])+y[t]*log(1-pr[t])+loggam(y[t]+ r)- loggam(y[t]+1) - loggam(r)
	#}
	
	##priors
	r~dgamma(0.01,0.01)
	for(i in 1:5){
		beta[i]~dnorm(0,0.001)
	}
	##priors for phi's
	phi[1]<-0 #use this option if phi[1] is omitted
	phi[2]<-0 #use this option if phi[2] is omitted
	#phi[1] <-y.phi[1,p] #use this option if phi[1] is included
	#phi[2] <-y.phi[2,p] #use this option if phi[2] is included
	phi[3] <-y.phi[3,p] 
	
	#for (k in 1:p){
	#	phi[k] <-y.phi[k,p]
	#}
	
	for (k in 1:p){
		alpha.phi[k] <-round(0.5*(k+1)-0.01)
		beta.phi[k] <-round((0.5*k)+1-0.01)
		r.phi[k] ~dbeta(alpha.phi[k], beta.phi[k])
		r.phi.map[k] <-2*r.phi[k]-1
	}
	y.phi[1,1]<-r.phi.map[1]
	for (k in 2:p){
		for (i in 1:(k-1)){
		y.phi[i,k] <-y.phi[i,k-1]-r.phi.map[k]*y.phi[k-i,k-1]
		}
		y.phi[k,k] <-r.phi.map[k]
	}
}
write.model(model310SOHx, con = "model310SOHx.txt")

w<-16
c<-1
p<-3
N<-408
parameters<-c("r", "phi", "beta", paste("y.p1[",w+1,":",N,"]",sep=""), "Dev") 
data<-list(w=w, c=c, pi = 3.14159265358979, N=N, p=p, y=y[1:N], rainlag2= x[1:N])
##In case of fitting to half of the series, and predicting the other:
#N<-204
#M<-408
#parameters<-c("r", "phi", "beta", paste("y.p1[",w+1,":",M,"]",sep=""), "Dev") 
#data<-list(w=w, c=c, pi = 3.14159265358979, N=N, M=M, p=p, y=y[1:M], rainlag2= x[1:M])


inits<- list(list(r=10, beta=rep(0,5)), list(r=5, beta=rep(1,5)), list(r=2, beta=rep(-1,5)))

##estimation:
ptm <- proc.time()
jags.output.model310SOHx <- jags(data= data, inits, parameters, model.file= "model310SOHx.txt",
    n.iter=11000, n.burnin=1000, n.thin=1, n.chains=3)
proc.time() - ptm
alarm()

#module glm loaded
#Compiling model graph
#   Resolving undeclared variables
#   Allocating nodes
#   Graph Size: 16699
#
#   user  system elapsed 
#1773.23    3.92 1802.89

print(jags.output.model310SOHx, digits=2)
#Inference for Bugs model at "model310SOHx.txt", fit using jags,
# 3 chains, each with 11000 iterations (first 1000 discarded)
# n.sims = 30000 iterations saved
#          mu.vect sd.vect    2.5%     25%     50%     75%   97.5% Rhat n.eff
#Dev       4326.20    3.77 4320.87 4323.43 4325.51 4328.27 4335.27    1 13000
#beta[1]     -0.34    0.16   -0.65   -0.44   -0.34   -0.23   -0.02    1  4800
#beta[2]     -0.10    0.07   -0.23   -0.15   -0.10   -0.06    0.03    1  9800
#beta[3]     -0.15    0.07   -0.27   -0.19   -0.15   -0.10   -0.02    1  7100
#beta[4]      0.14    0.04    0.06    0.11    0.14    0.16    0.21    1 26000
#beta[5]      0.16    0.04    0.07    0.13    0.16    0.18    0.24    1 11000
#phi[1]       0.00    0.00    0.00    0.00    0.00    0.00    0.00    1     1
#phi[2]       0.00    0.00    0.00    0.00    0.00    0.00    0.00    1     1
#phi[3]      -0.10    0.05   -0.19   -0.13   -0.10   -0.07    0.00    1 30000
#r            4.54    0.36    3.87    4.29    4.53    4.78    5.27    1 30000
#y.p1[17]    62.15   30.46   17.00   40.00   57.00   79.00  134.00    1 30000
#y.p1[408]    3.65    2.60    0.00    2.00    3.00    5.00   10.00    1 30000
#deviance  4326.20    3.77 4320.87 4323.43 4325.51 4328.27 4335.27    1 13000
#
#For each parameter, n.eff is a crude measure of effective sample size,
#and Rhat is the potential scale reduction factor (at convergence, Rhat=1).
#
#DIC info (using the rule, pD = var(deviance)/2)
#pD = 7.1 and DIC = 4333.3
#DIC is an estimate of expected predictive error (lower deviance is better).

getwd()
save(jags.output.model310SOHx, file= "outmodel310SOHx1000.Rdata")
#load("outmodel310SOHx1000.Rdata")

res.mcmc<-as.mcmc(jags.output.model310SOHx)
res.mcmc.sel<-res.mcmc[][,1:5]
res.list<-mcmc.list(res.mcmc.sel[[1]],res.mcmc.sel[[2]],res.mcmc.sel[[2]])
gelman.diag(res.list)
#Potential scale reduction factors:
#
#        Point est. Upper C.I.
#Dev              1          1
#beta[1]          1          1
#beta[2]          1          1
#beta[3]          1          1
#beta[4]          1          1
#
#Multivariate psrf
#
#1

re<-(jags.output.model310SOHx$BUGSoutput$median$y.p1[1:(N-w)]-y[(w+1):N])/ (y[(w+1):N]+1)
mean(abs(re))
#[1] 0.386191

#In case of out of sample prediction on the second half of the series
#re<-(jags.output.model310SOHxpred$BUGSoutput$median$y.p1[(N-w+1):(M-w)]-y[(N+1):M])/ (y[(N+1):M]+1)
#mean(abs(re))
#[1] 0.3858906


loop.rrpv<-function(n, y, output){
	y.p1<-output$BUGSoutput $sims.list$y.p1[,(n)]
	F<-ecdf(y.p1)
	rrpv<- runif(1,F(y[(n+w)]-1), F(y[(n+w)]))
}

rrpv.310SOHx<- apply(as.matrix(1: (N-w)), MARGIN = 1, loop.rrpv, y= y, output= jags.output.model310SOHx)

plot(ecdf(rrpv.310SOHx), verticals=TRUE, do.p = FALSE, xlab = "Randomized residual probability", ylab = "Cumulative distribution of randomized cumulative probabilities", main = NULL, col.01line = NULL)
lines(c(0,1),c(0,1), col=8)

loop.ptest<-function(n, N){
	test<-binom.test(n, N, n/N)
	low <-test$conf.int[1]
	high <-test$conf.int[2]
	res<-c(low, high)
	names(res)<-c("low", "high")
	res
}
bounds<- as.data.frame(t(apply(as.matrix(4: (N-w-4)), MARGIN = 1, loop.ptest, N=(N-16))))
z<-(4: (N-w-4))/(N-w)
lines(z,bounds$low, lty=2, col=8)
lines(z,bounds$high, lty=2, col=8)


len<-length(rrpv.310SOHx)
length(rrpv.310SOHx[c(len-49): len])
##Last 50 only
plot(ecdf(rrpv.310SOHx[c(len-49): len]), verticals=TRUE, do.p = FALSE, xlab = "Randomized residual probability", ylab = "Cumulative distribution of randomized cumulative probabilities", main = NULL, col.01line = NULL)
lines(c(0,1),c(0,1), col=8)
bounds<- as.data.frame(t(apply(as.matrix(1: (50-1)), MARGIN = 1, loop.ptest, N=50)))
z<-(1: (50-1))/(50)
lines(z,bounds$low, lty=2, col=8)
lines(z,bounds$high, lty=2, col=8)

model310SOHxid<-function(){
	##Deviance
	Dev <- -2*sum(LL[(w+1) : N])
	##
	for (t in 1:w){
		x[1,t]<-rainlag2[t]/1000
		x[2,t]<-sin(2*pi*t/12)
		x[3,t]<- cos(2*pi*t/12)
		x[4,t]<- sin(2*pi*t/6)
		x[5,t]<- cos(2*pi*t/6)
	}
	for (t in (w+1):N){
		y[t]~dnegbin(pr[t],r)
		y.p1[t]~dnegbin(pr[t],r)
		pr[t]<-r/(r+lambda[t])
		lambda[t]<-max(0.000000001,m[t])
		#lambda[t]<-exp(m[t])
		m[t]<- inprod(beta[],x[,t]) + ((y[t-1])) - 	inprod(beta[],x[,t-1]) + sum(AR[ ,t])
		for (k in 1:p){
			AR[k,t]<-
			 (phi[k])*((y[t-k]))
			-phi[k] * inprod(beta[],x[,t-k])
			-(phi[k]) * ((y[t-k-1]))
			+phi[k] * inprod(beta[],x[,t-k-1])
		}
		x[1,t]<-rainlag2[t]/1000
		x[2,t]<-sin(2*pi*t/12)
		x[3,t]<- cos(2*pi*t/12)
		x[4,t]<- sin(2*pi*t/6)
		x[5,t]<- cos(2*pi*t/6)
		LL[t] <-r*log(pr[t])+y[t]*log(1-pr[t])+loggam(y[t]+ r)- loggam(y[t]+1) - loggam(r)
	}
	##Prediction out of sample (activate in case N<M) 
	#for (t in (N+1):M){
	#	y.p1[t]~dnegbin(pr[t],r)
	#	pr[t]<-r/(r+lambda[t])
	#	lambda[t]<-max(0.000000001,m[t])
	#	m[t]<- inprod(beta[],x[,t]) + ((y[t-1])) - inprod(beta[],x[,t-1]) + sum(AR[ ,t])
	#	for (k in 1:p){
	#		AR[k,t]<-
	#		 (phi[k])*((y[t-k]))
	#		-phi[k] * inprod(beta[],x[,t-k])
	#		-(phi[k]) * ((y[t-k-1]))
	#		+phi[k] * inprod(beta[],x[,t-k-1])
	#	}
	#	x[1,t]<-rainlag2[t]/1000
	#	x[2,t]<-sin(2*pi*t/12)
	#	x[3,t]<- cos(2*pi*t/12)
	#	x[4,t]<- sin(2*pi*t/6)
	#	x[5,t]<- cos(2*pi*t/6)
	#	LL[t] <-r*log(pr[t])+y[t]*log(1-pr[t])+loggam(y[t]+ r)- loggam(y[t]+1) - loggam(r)
	#}
	
	##priors
	r~dgamma(0.01,0.01)
	for(i in 1:5){
		beta[i]~dnorm(0,0.001)
	}
	##priors for phi's
	phi[1]<-0 #use this option if phi[1] is omitted
	phi[2]<-0 #use this option if phi[2] is omitted
	#phi[1] <-y.phi[1,p] #use this option if phi[1] is included
	#phi[2] <-y.phi[2,p] #use this option if phi[2] is included
	phi[3] <-y.phi[3,p] 
	
	#for (k in 1:p){
	#	phi[k] <-y.phi[k,p]
	#}
	
	for (k in 1:p){
		alpha.phi[k] <-round(0.5*(k+1)-0.01)
		beta.phi[k] <-round((0.5*k)+1-0.01)
		r.phi[k] ~dbeta(alpha.phi[k], beta.phi[k])
		r.phi.map[k] <-2*r.phi[k]-1
	}
	y.phi[1,1]<-r.phi.map[1]
	for (k in 2:p){
		for (i in 1:(k-1)){
		y.phi[i,k] <-y.phi[i,k-1]-r.phi.map[k]*y.phi[k-i,k-1]
		}
		y.phi[k,k] <-r.phi.map[k]
	}
}
write.model(model310SOHxid, con = "model310SOHxid.txt")

w<-16
c<-1
p<-3
N<-408
parameters<-c("r", "phi", "beta", paste("y.p1[",w+1,":",N,"]",sep=""), "Dev") 
data<-list(w=w, pi = 3.14159265358979, N=N, p=p, y=y[1:N], rainlag2= x[1:N])


inits<- list(list(r=10, beta=rep(0,5)), list(r=5, beta=rep(1,5)), list(r=2, beta=rep(-1,5)))

##estimation:
ptm <- proc.time()
jags.output.model310SOHxid <- jags(data= data, inits, parameters, model.file= "model310SOHxid.txt",
    n.iter=11000, n.burnin=1000, n.thin=1, n.chains=3)
proc.time() - ptm
alarm()


print(jags.output.model310SOHxid, digits=2)

#Inference for Bugs model at "model310SOHxid.txt", fit using jags,
# 3 chains, each with 11000 iterations (first 1000 discarded)
# n.sims = 30000 iterations saved
#          mu.vect sd.vect    2.5%     25%     50%     75%   97.5%  Rhat n.eff
#Dev       4393.90   23.72 4356.56 4362.58 4407.96 4411.00 4417.81 12.50     3
#beta[1]     -6.51    0.88   -8.15   -7.11   -6.55   -5.94   -4.69  1.11    23
#beta[2]     -1.31    0.41   -2.10   -1.58   -1.33   -1.05   -0.46  1.03  1300
#beta[3]     -0.75    1.10   -2.24   -1.64   -1.20    0.56    1.02  5.04     3
#beta[4]      0.86    0.58   -0.09    0.19    1.05    1.33    1.71  4.25     3
#beta[5]      0.71    0.27    0.22    0.51    0.70    0.91    1.27  1.68     6
#phi[1]       0.00    0.00    0.00    0.00    0.00    0.00    0.00  1.00     1
#phi[2]       0.00    0.00    0.00    0.00    0.00    0.00    0.00  1.00     1
#phi[3]      -0.07    0.04   -0.12   -0.10   -0.08   -0.03    0.00  3.60     4
#r            4.31    0.35    3.66    4.06    4.30    4.54    5.01  1.04    62
#y.p1[17]    52.34   26.36   14.00   33.00   48.00   67.00  115.00  1.01   410
#y.p1[408]    1.50    1.51    0.00    0.00    1.00    2.00    5.00  1.02   170
#deviance  4393.90   23.72 4356.56 4362.58 4407.96 4411.00 4417.81 12.50     3
#
#For each parameter, n.eff is a crude measure of effective sample size,
#and Rhat is the potential scale reduction factor (at convergence, Rhat=1).
#
#DIC info (using the rule, pD = var(deviance)/2)
#pD = 5.9 and DIC = 4399.8
#DIC is an estimate of expected predictive error (lower deviance is better).
getwd()
save(jags.output.model310SOHxid, file= "outmodel310SOHx1000id.Rdata")
#load("outmodel310SOHx1000.Rdata")

res.mcmc<-as.mcmc(jags.output.model310SOHxid)
res.mcmc.sel<-res.mcmc[][,1:5]
res.list<-mcmc.list(res.mcmc.sel[[1]],res.mcmc.sel[[2]],res.mcmc.sel[[2]])
gelman.diag(res.list)

#Potential scale reduction factors:
#
#        Point est. Upper C.I.
#Dev          12.27      23.48
#beta[1]       1.11       1.33
#beta[2]       1.03       1.04
#beta[3]       4.89      10.23
#beta[4]       4.12       8.56
#
#Multivariate psrf
#
#10.9

re<-(jags.output.model310SOHxid$BUGSoutput$median$y.p1[1:(N-w)]-y[(w+1):N])/ (y[(w+1):N]+1)
mean(abs(re))
#[1] 0.3999568


model310SOHxpredid<-function(){
	##Deviance
	Dev <- -2*sum(LL[(w+1) : N])
	##
	for (t in 1:w){
		x[1,t]<-rainlag2[t]/1000
		x[2,t]<-sin(2*pi*t/12)
		x[3,t]<- cos(2*pi*t/12)
		x[4,t]<- sin(2*pi*t/6)
		x[5,t]<- cos(2*pi*t/6)
	}
	for (t in (w+1):N){
		y[t]~dnegbin(pr[t],r)
		y.p1[t]~dnegbin(pr[t],r)
		pr[t]<-r/(r+lambda[t])
		lambda[t]<-max(0.000000001,m[t])
		#lambda[t]<-exp(m[t])
		m[t]<- inprod(beta[],x[,t]) + ((y[t-1])) - 	inprod(beta[],x[,t-1]) + sum(AR[ ,t])
		for (k in 1:p){
			AR[k,t]<-
			 (phi[k])*((y[t-k]))
			-phi[k] * inprod(beta[],x[,t-k])
			-(phi[k]) * ((y[t-k-1]))
			+phi[k] * inprod(beta[],x[,t-k-1])
		}
		x[1,t]<-rainlag2[t]/1000
		x[2,t]<-sin(2*pi*t/12)
		x[3,t]<- cos(2*pi*t/12)
		x[4,t]<- sin(2*pi*t/6)
		x[5,t]<- cos(2*pi*t/6)
		LL[t] <-r*log(pr[t])+y[t]*log(1-pr[t])+loggam(y[t]+ r)- loggam(y[t]+1) - loggam(r)
	}
	##Prediction out of sample (activate in case N<M) 
	for (t in (N+1):M){
		y.p1[t]~dnegbin(pr[t],r)
		pr[t]<-r/(r+lambda[t])
		lambda[t]<-max(0.000000001,m[t])
		m[t]<- inprod(beta[],x[,t]) + ((y[t-1])) - inprod(beta[],x[,t-1]) + sum(AR[ ,t])
		for (k in 1:p){
			AR[k,t]<-
			 (phi[k])*((y[t-k]))
			-phi[k] * inprod(beta[],x[,t-k])
			-(phi[k]) * ((y[t-k-1]))
			+phi[k] * inprod(beta[],x[,t-k-1])
		}
		x[1,t]<-rainlag2[t]/1000
		x[2,t]<-sin(2*pi*t/12)
		x[3,t]<- cos(2*pi*t/12)
		x[4,t]<- sin(2*pi*t/6)
		x[5,t]<- cos(2*pi*t/6)
		LL[t] <-r*log(pr[t])+y[t]*log(1-pr[t])+loggam(y[t]+ r)- loggam(y[t]+1) - loggam(r)
	}
	
	##priors
	r~dgamma(0.01,0.01)
	for(i in 1:5){
		beta[i]~dnorm(0,0.001)
	}
	##priors for phi's
	phi[1]<-0 #use this option if phi[1] is omitted
	phi[2]<-0 #use this option if phi[2] is omitted
	#phi[1] <-y.phi[1,p] #use this option if phi[1] is included
	#phi[2] <-y.phi[2,p] #use this option if phi[2] is included
	phi[3] <-y.phi[3,p] 
	
	#for (k in 1:p){
	#	phi[k] <-y.phi[k,p]
	#}
	
	for (k in 1:p){
		alpha.phi[k] <-round(0.5*(k+1)-0.01)
		beta.phi[k] <-round((0.5*k)+1-0.01)
		r.phi[k] ~dbeta(alpha.phi[k], beta.phi[k])
		r.phi.map[k] <-2*r.phi[k]-1
	}
	y.phi[1,1]<-r.phi.map[1]
	for (k in 2:p){
		for (i in 1:(k-1)){
		y.phi[i,k] <-y.phi[i,k-1]-r.phi.map[k]*y.phi[k-i,k-1]
		}
		y.phi[k,k] <-r.phi.map[k]
	}
}
write.model(model310SOHxpredid, con = "model310SOHxpredid.txt")

w<-16
c<-1
p<-3
N<-408
parameters<-c("r", "phi", "beta", paste("y.p1[",w+1,":",N,"]",sep=""), "Dev") 
data<-list(w=w, pi = 3.14159265358979, N=N, p=p, y=y[1:N], rainlag2= x[1:N])
##In case of fitting to half of the series, and predicting the other:
N<-204
M<-408
parameters<-c("r", "phi", "beta", paste("y.p1[",w+1,":",M,"]",sep=""), "Dev") 
data<-list(w=w,pi = 3.14159265358979, N=N, M=M, p=p, y=y[1:M], rainlag2= x[1:M])


inits<- list(list(r=10, beta=rep(0,5)), list(r=5, beta=rep(1,5)), list(r=2, beta=rep(-1,5)))

##estimation:
ptm <- proc.time()
jags.output.model310SOHxpredid <- jags(data= data, inits, parameters, model.file= "model310SOHxpredid.txt",
    n.iter=11000, n.burnin=1000, n.thin=1, n.chains=3)
proc.time() - ptm
alarm()


print(jags.output.model310SOHxpredid, digits=2)

#Inference for Bugs model at "model310SOHxpredid.txt", fit using jags,
# 3 chains, each with 11000 iterations (first 1000 discarded)
# n.sims = 30000 iterations saved
#          mu.vect sd.vect    2.5%     25%     50%     75%   97.5% Rhat n.eff
#Dev       2205.52    3.69 2200.21 2202.79 2204.90 2207.56 2214.36 1.00  6600
#beta[1]    -24.66    6.78  -37.57  -29.28  -24.77  -20.15  -10.92 1.00   710
#beta[2]      0.88    3.21   -4.89   -1.38    0.69    2.95    7.71 1.00  1600
#beta[3]     -2.16    1.96   -5.81   -3.50   -2.23   -0.91    1.89 1.00  9200
#beta[4]      1.67    2.27   -3.11    0.20    1.80    3.28    5.73 1.00   700
#beta[5]      4.42    1.25    1.81    3.61    4.48    5.30    6.70 1.00  1400
#phi[1]       0.00    0.00    0.00    0.00    0.00    0.00    0.00 1.00     1
#phi[2]       0.00    0.00    0.00    0.00    0.00    0.00    0.00 1.00     1
#phi[3]      -0.01    0.03   -0.07   -0.03   -0.01    0.01    0.05 1.00  3700
#r            3.91    0.42    3.13    3.63    3.90    4.19    4.76 1.00 30000
#y.p1[17]    50.11   26.38   12.00   31.00   46.00   65.00  113.00 1.00 30000
#y.p1[408]    0.24    1.00    0.00    0.00    0.00    0.00    3.00 1.01  1900
#deviance  2205.52    3.69 2200.21 2202.79 2204.90 2207.56 2214.36 1.00  6600
#
#For each parameter, n.eff is a crude measure of effective sample size,
#and Rhat is the potential scale reduction factor (at convergence, Rhat=1).
#
#DIC info (using the rule, pD = var(deviance)/2)
#pD = 6.8 and DIC = 2212.3
#DIC is an estimate of expected predictive error (lower deviance is better).

getwd()
save(jags.output.model310SOHxpredid, file= "outmodel310SOHx1000predid.Rdata")
#load("outmodel310SOHx1000predid.Rdata")

res.mcmc<-as.mcmc(jags.output.model310SOHxpredid)
res.mcmc.sel<-res.mcmc[][,1:5]
res.list<-mcmc.list(res.mcmc.sel[[1]],res.mcmc.sel[[2]],res.mcmc.sel[[3]])
gelman.diag(res.list)

#Potential scale reduction factors:
#
#        Point est. Upper C.I.
#Dev              1       1.00
#beta[1]          1       1.00
#beta[2]          1       1.00
#beta[3]          1       1.00
#beta[4]          1       1.01
#
#Multivariate psrf

#In case of out of sample prediction on the second half of the series
re<-(jags.output.model310SOHxpredid$BUGSoutput$median$y.p1[(N-w+1):(M-w)]-y[(N+1):M])/ (y[(N+1):M]+1)
mean(abs(re))
#[1] 0.3979054


library(R2jags)
model310100<-function(){
	##Priors
#	beta~dnorm(0,0.001)
	beta<-0 #use this option in a model without external variable
	r~dgamma(0.01,0.01)
#	phi.star<-0
	#phi.star~dunif(-0.999999, 0.999999)
	r.phi.star ~dbeta(1,1)
	phi.star <-2*r.phi.star -1
	phi[1]<-0
	phi[2]<-0
	#phi[3]~dunif(-0.999999, 0.999999)
	r.phi ~dbeta(1,1)
	phi[3]<-2*r.phi -1

	##Ljung-Box test
	Chlag ~ dchisqr(df)
	df<-lag-2
	##Ljung-Box test on Pearson residuals e
	for(k in 1: lag){
		for(t in (k + 1 +3+1+12): (N)){
			e.lag[k,t] <- (y[t-k]-lambda[t-k])/sqrt(V[t-k])
			p1[k,t] <- e[t]*e.lag[k,t]
		}
		auto[k] <- sum(p1[k,(k + 1 +3+1+12):(N)])/sum(e2[(1 +3+1+12) : (N)])
		auto2[k] <- auto[k]*auto[k]
		auto2div[k] <- auto2[k]/(N-k)
	}
	LB <-  N*(N+2)*sum(auto2div[])
	P.LB <- step(LB-Chlag)
	
	##Ljung-Box test on relative residuals eta
	for(k in 1: lag){
		for(t in (k + 1 +3+1+12): (N)){
			eta.lag[k,t] <- (y[t-k]-lambda[t-k])/sqrt(V[t-k])
			p1.eta[k,t] <- eta[t]*eta.lag[k,t]
		}
		auto.eta[k] <- sum(p1.eta[k,(k + 1 +3+1+12):(N)])/sum(eta2[(1 +3+1+12) : (N)])
		auto.eta2[k] <- auto.eta[k]*auto.eta[k]
		auto.eta2div[k] <- auto.eta2[k]/(N-k)
	}
	LB.eta <-  N*(N+2)*sum(auto.eta2div[])
	P.LB.eta <- step(LB.eta-Chlag)
		
	##Predictive check on overdispersion
	Diagobs <- pow(sd(y[(1 +3+1+12) : (N)]),2)/mean(y[(1 +3+1+12) : (N)])
	Diagnew <- pow(sd(y.p1[(1 +3+1+12) : (N)]),2)/mean(y.p1[(1 +3+1+12) : (N)])
	PrCh <- step(Diagnew-Diagobs)
	
	##Deviance
	Dev <- -2*sum(LL[(3+1+12+1) : (N)])
	
	##Predicting preseries latent observations
	for (t in 1: (12+3+1)){
		u[t] <- 0
	}
	
	##likelihood
	for (t in (12+3+1+1):N){
		y[t]~dnegbin(pr[t],r)
		y.p1[t]~dnegbin(pr[t],r)
		pr[t]<-r/(r+lambda[t])
		lambda[t]<-exp(m[t])
		m[t] <- beta * x[t] 
+log(max(c,y[t-1])) 
-beta * x[t-1] 
+ sum(AR[ ,t]) + sum(ARSAR[ ,t]) + SAR[t]
		for (k in 1:p){
			AR[k,t]<-
			 (phi[k])*log(max(c,y[t-k]))
			-phi[k] * beta * x[t-k] 
			-(phi[k])*log(max(c,y[t-k-1]))
			+phi[k] * beta * x[t-k-1]
			ARSAR[k,t]<- 
			-(phi.star)*(phi[k])*log(max(c,y[t-k-12]))
			+phi.star*phi[k] * beta * x[t-k-12] 
			+(phi.star) *(phi[k])*log(max(c,y[t-k-1-12])) 
			-phi.star*phi[k] * beta * x[t-k-1-12] 
		}
		SAR[t]<-
		 (phi.star)*log(max(c,y[t-12])) 
		-phi.star * beta * x[t-12]
		-(phi.star)*log(max(c,y[t-1-12]))
		+ phi.star * beta * x[t-1-12]
		u[t] <- log(max(c,y[t])/lambda[t])
		V[t] <- lambda[t]+ lambda[t]*lambda[t]/r
		e[t] <- (y[t]-lambda[t])/sqrt(V[t])
		e2[t] <- e[t]*e[t]
		eta[t] <- (y[t]-lambda[t])/(y[t]+1)
		eta2[t] <- eta[t]*eta[t]
		LL[t] <-r*log(pr[t])+y[t]*log(1-pr[t])+loggam(y[t]+r)- loggam(y[t]+1) - loggam(r)
	}
##prediction out of series(activate in case N<M) 
	#for (t in (N+1):M){
#		y.p1[t]~dnegbin(pr[t],r)
#		pr[t]<-r/(r+lambda[t])
#		lambda[t]<-exp(m[t])
#		m[t] <- beta * x[t] 
#			+log(max(c,y[t-1])) 
#			-beta * x[t-1] 
#			+ sum(AR[ ,t]) + sum(ARSAR[ ,t]) + SAR[t]
#		for (k in 1:p){
#			AR[k,t]<-
#			 (phi[k])*log(max(c,y[t-k]))
#			-phi[k] * beta * x[t-k] 
#			-(phi[k])*log(max(c,y[t-k-1]))
#			+phi[k] * beta * x[t-k-1]
#			ARSAR[k,t]<- 
#			-(phi.star)*(phi[k])*log(max(c,y[t-k-12]))
#			+phi.star*phi[k] * beta * x[t-k-12] 
#			+(phi.star) *(phi[k])*log(max(c,y[t-k-1-12])) 
#			-phi.star*phi[k] * beta * x[t-k-1-12] 
#		}
#		SAR[t]<-
#		 (phi.star)*log(max(c,y[t-12])) 
#		-phi.star * beta * x[t-12]
#		-(phi.star)*log(max(c,y[t-1-12]))
#		+ phi.star * beta * x[t-1-12]
#		u[t] <- log(max(c,y[t])/lambda[t])
#}
}
write.model(model310100, con = "model310100.txt")

w<-16
N<-408
parameters<-c("r", "phi", "phi.star", paste("y.p1[",w+1,":",N,"]",sep=""), "LB", "Chlag", "P.LB", "Dev", "Diagnew", "PrCh", "P.LB.eta", "LB.eta") 
data<-list(lag=15, c=1, N=N, p=3, y= y[1:N], x=x[1:N]/1000)

#In case of predicting on out of sample part of the series
#M<-408
#N<-204
#parameters<-c("r", "phi", "phi.star", paste("y.p1[",w+1,":",M,"]",sep=""), "LB", "Chlag", "P.LB", "Dev", "Diagnew", "PrCh", "P.LB.eta", "LB.eta") 
#data<-list(lag=15, c=1, N=N, M=M, p=3, y= y[1:M], x=x[1:M]/1000)

#estimation:
rnorm(1)
inits<- list(list(r=10, r.phi=0.1, r.phi.star=0.1), list(r=5, r.phi=0.5, r.phi.star=0.1), list(r=2, r.phi=0.9, r.phi.star=0.1))

ptm <- proc.time()
jags.output.model310100 <- jags(data= data, inits, parameters, model.file= "model310100.txt",
    n.iter=11000, n.burnin=1000, n.thin=1, n.chains=3)
proc.time() - ptm
alarm()

#module glm loaded
#Compiling model graph
#   Resolving undeclared variables
#   Allocating nodes
#   Graph Size: 33052
#
#   user  system elapsed 
#1377.45    2.72 1393.31

print(jags.output.model310100, digits=2)

#Inference for Bugs model at "model310100.txt", fit using jags,
# 3 chains, each with 11000 iterations (first 1000 discarded)
# n.sims = 30000 iterations saved
#          mu.vect sd.vect    2.5%     25%     50%     75%   97.5% Rhat n.eff
#Chlag       12.93    5.05    5.03    9.25   12.30   15.90   24.55    1 21000
#Dev       4347.64    2.46 4344.85 4345.86 4347.00 4348.73 4354.21    1 30000
#Diagnew    553.79  102.73  395.26  481.48  539.46  610.00  791.37    1 30000
#LB          28.33    1.15   26.64   27.57   28.15   28.87   31.18    1 10000
#LB.eta     285.14   34.67  223.82  260.94  282.85  307.19  358.65    1 30000
#P.LB         0.99    0.08    1.00    1.00    1.00    1.00    1.00    1 30000
#P.LB.eta     1.00    0.00    1.00    1.00    1.00    1.00    1.00    1     1
#PrCh         0.89    0.32    0.00    1.00    1.00    1.00    1.00    1 21000
#phi[1]       0.00    0.00    0.00    0.00    0.00    0.00    0.00    1     1
#phi[2]       0.00    0.00    0.00    0.00    0.00    0.00    0.00    1     1
3phi[3]      -0.13    0.05   -0.23   -0.16   -0.13   -0.10   -0.04    1 30000
#phi.star     0.12    0.05    0.03    0.09    0.12    0.15    0.22    1 28000
#r            4.33    0.35    3.69    4.09    4.32    4.56    5.04    1 30000
#y.p1[17]    57.61   29.14   15.00   36.00   53.00   73.00  128.00    1 30000
#y.p1[408]    3.65    2.58    0.00    2.00    3.00    5.00   10.00    1 17000
#deviance  4347.64    2.46 4344.85 4345.86 4347.00 4348.73 4354.21    1 30000
#
#For each parameter, n.eff is a crude measure of effective sample size,
#and Rhat is the potential scale reduction factor (at convergence, Rhat=1).
#
#DIC info (using the rule, pD = var(deviance)/2)
#pD = 3.0 and DIC = 4350.7
#DIC is an estimate of expected predictive error (lower deviance is better).

save(jags.output.model310100, file= "outmodel3101001000.Rdata")
#load("outmodel3101001000.Rdata")

res.mcmc<-as.mcmc(jags.output.model310100)
res.mcmc.sel<-res.mcmc[][,8:9]

res.mcmc.sel<-res.mcmc[][,12:13]
res.list<-mcmc.list(res.mcmc.sel[[1]],res.mcmc.sel[[2]],res.mcmc.sel[[2]])
gelman.diag(res.list)

#Potential scale reduction factors:
#
#         Point est. Upper C.I.
#phi[3]            1          1
#phi.star          1          1
#
#Multivariate psrf
#
#1
gelman.plot(res.list)

re<-(jags.output.model310100$BUGSoutput$median$y.p1[1:(N-w)]-y[(w+1):N])/ (y[(w+1):N]+1)
mean(abs(re))
#[1] 0.3883272

loop.rrpv<-function(n, y, output){
	y.p1<-jags.output.model310100$BUGSoutput$sims.list$y.p1[,(n)]
	F<-ecdf(y.p1)
	rrpv<- runif(1,F(y[(n+w)]-1), F(y[(n+w)]))
}

rrpv.model310100 <- apply(as.matrix(1: (N-w)), MARGIN = 1, loop.rrpv, y= y, output= jags.output.model310100)

plot(ecdf(rrpv.model310100), verticals=TRUE, do.p = FALSE, xlab = "Randomized residual probability", ylab = "Cumulative distribution of randomized cumulative probabilities", main = NULL, col.01line = NULL)
lines(c(0,1),c(0,1), col=8)

loop.ptest<-function(n, N){
	test<-binom.test(n, N, n/N)
	low <-test$conf.int[1]
	high <-test$conf.int[2]
	res<-c(low, high)
	names(res)<-c("low", "high")
res
}
bounds<- as.data.frame(t(apply(as.matrix(4: (N-w-4)), MARGIN = 1, loop.ptest, N=(N-16))))
z<-(4: (N-w-4))/(N-w)
lines(z,bounds$low, lty=2, col=8)
lines(z,bounds$high, lty=2, col=8)

#plot.stepfun(rrpv.model310100, col.hor=1, col.vert=1, do.points=FALSE, add=TRUE)

len<-length(rrpv.model310100)
length(rrpv.model310100[c(len-49): len])

##Last 50 only
plot(ecdf(rrpv.model310100[c(len-49): len]), verticals=TRUE, do.p = FALSE, xlab = "Randomized residual probability", ylab = "Cumulative distribution of randomized cumulative probabilities", main = NULL, col.01line = NULL)
lines(c(0,1),c(0,1), col=8)
bounds<- as.data.frame(t(apply(as.matrix(1: (50-1)), MARGIN = 1, loop.ptest, N=50)))
z<-(1: (50-1))/(50)
lines(z,bounds$low, lty=2, col=8)
lines(z,bounds$high, lty=2, col=8)

nrqr<-qnorm(pmax(0.00001, pmin(0.99999, rrpv.model310100)))
nrqr.ts<-ts(nrqr, start=c(1973,5), freq=12)
plot(nrqr.ts, type = "p", ylab="Normalized randomized quantile residuals") #Figure S4

pdf(file = "FigureS4.pdf",
     width = 6.83, height = 6.83, pointsize = 8,
     bg = "white", family = "Arial")
op <- par(mar=c(4,4,4,2),omi=c(0.02778, 0.02778, 0.02778, 0.02778), mgp=c(2.5,1,0), cex.lab=1.4)
plot(nrqr.ts, type = "p", ylab="Normalized randomized quantile residuals", main=expression(paste("Normalized randomized quantile residuals of negative binomial GSARIMA ", group("(",list("3'", 1, 0),")") %*% group("(",list(1, 0, 0),")")[12], " model")))
par(op)
dev.off()


group("(",list(1, 0),")")[12]


plot(density(nrqr))
qqnorm(nrqr)
qqline(nrqr)
#Figure 4 in main text


tiff(filename = "Figure4.tif",
     width = 8.3, height = 8.3, units = "cm", pointsize = 8,
     compression = c("lzw"),
     bg = "white", res = 300, family = "Arial", restoreConsole = TRUE,
     type = c("cairo"), antialias=c("subpixel"))
op <- par(mar=c(3.6,3.6,0,0),omi=c(0.02778, 0.02778, 0.02778, 0.02778), mgp=c(2.5,1,0), cex.lab=1.4)
qqnorm(nrqr, main=NULL, xlab="Theoretical quantiles", ylab="Sample quantiles")
qqline(nrqr)
par(op)
dev.off()


plot(diff(log(tsy+1)))
lmres<-lm(nrqr[2: (N-w)]~ diff(log(y[(w+1):N]+1)))
summary(lmres)
#Call:
#lm(formula = nrqr[2:(N - w)] ~ diff(log(y[(w + 1):N] + 1)))
#
#Residuals:
#     Min       1Q   Median       3Q      Max 
#-1.29898 -0.13928 -0.03403  0.10211  1.75384 
#
#Coefficients:
#                            Estimate Std. Error t value Pr(>|t|)    
#(Intercept)                  0.22477    0.01398   16.08   <2e-16 ***
#diff(log(y[(w + 1):N] + 1))  1.85043    0.02627   70.44   <2e-16 ***
#---
#Signif. codes:  0 '***' 0.001 '**' 0.01 '*' 0.05 '.' 0.1 ' ' 1 
#
#Residual standard error: 0.2764 on 389 degrees of freedom
#Multiple R-squared: 0.9273,     Adjusted R-squared: 0.9271 
#F-statistic:  4962 on 1 and 389 DF,  p-value: < 2.2e-16 

plot(density(nrqr))

plot(diff(log(y[(w+1):N]+1)), nrqr[2:(N-w)], ylab= "Normalized randomized quantile residuals", xlab="Differenced log transformed monthly malaria case counts")
lines(c(-3,3), c(lmres$coef[1]+ -3*lmres$coef[2], lmres$coef[1]+ 3*lmres$coef[2]))

#Figure 5 in article 


tiff(filename = "Figure5.tif",
     width = 8.3, height = 8.3, units = "cm", pointsize = 8,
     compression = c("lzw"),
     bg = "white", res = 300, family = "Arial", restoreConsole = TRUE,
     type = c("cairo"), antialias=c("subpixel"))
op <- par(mar=c(3.6,3.6,0,0),omi=c(0.02778, 0.02778, 0.02778, 0.02778), mgp=c(2.5,1,0), cex.lab=1.2)
plot(diff(log(y[(w+1):N]+1)), nrqr[2:(N-w)], ylab= "Normalized randomized quantile residuals", xlab="Differenced log transformed monthly cases")
lines(c(-3,3), c(lmres$coef[1]+ -3*lmres$coef[2], lmres$coef[1]+ 3*lmres$coef[2]))
par(op)
dev.off()


acf(nrqr, lag.max=36)
#Figure 6 in article

tiff(filename = "Figure6.tif",
     width = 8.3, height = 8.3, units = "cm", pointsize = 8,
     compression = c("lzw"),
     bg = "white", res = 300, family = "Arial", restoreConsole = TRUE,
     type = c("cairo"), antialias=c("subpixel"))
op <- par(mar=c(3.6,3.6,0,0),omi=c(0.02778, 0.02778, 0.02778, 0.02778), mgp=c(2.5,1,0), cex.lab=1.4)
acf(nrqr, lag.max=36, main=NULL, ylab="Autocorrelation function")
par(op)
dev.off()


pacf(nrqr, lag.max=36)

bt<-Box.test(nrqr, lag = 24, type = "Ljung-Box")
bt$statistic
#X-squared 
# 19.84402

pchisq(q= bt$statistic[[1]], df=24-1, lower.tail=FALSE)
#[1] 0.6513021

qchisq(p=0.95, df=(24-1))
#[1] 35.17246

#A comparisson with a Gaussian version of this model
library(R2jags)
modelG310100<-function(){
	##priors
#	beta~dnorm(0,0.001)
	beta<-0 #use this option in a model without external variable
	tau~dgamma(0.01,0.01)
#	phi.star<-0
	#phi.star~dunif(-0.999999, 0.999999)
	r.phi.star ~dbeta(1,1)
	phi.star <-2*r.phi.star -1
	phi[1]<-0
	phi[2]<-0
	#phi[3]~dunif(-0.999999, 0.999999)
	r.phi ~dbeta(1,1)
	phi[3]<-2*r.phi -1
	
	##Predicting preseries latent observations
	for (t in 1: (12+3+1)){
		u[t] <- 0
	}
	
	##likelihood
	for (t in (12+3+1+1):N){
		y.bc[t]~dnorm(m[t],tau)
		y.bc.p1[t]~dnorm(m[t],tau)
		m[t] <- beta * x[t] 
+ y.bc[t-1] 
-beta * x[t-1] 
+ sum(AR[ ,t]) + sum(ARSAR[ ,t]) + SAR[t]
		for (k in 1:p){
			AR[k,t]<-
 (phi[k])* y.bc[t-k]
-phi[k] * beta * x[t-k] 
-(phi[k])* y.bc[t-k-1]
+phi[k] * beta * x[t-k-1]
			ARSAR[k,t]<- 
-(phi.star)*(phi[k])* y.bc[t-k-12]
+phi.star*phi[k] * beta * x[t-k-12] 
+(phi.star) *(phi[k])* y.bc[t-k-1-12] 
-phi.star*phi[k] * beta * x[t-k-1-12] 
		}
		SAR[t]<-
 (phi.star)* y.bc[t-12] 
-phi.star * beta * x[t-12]
-(phi.star)* y.bc[t-1-12]
+ phi.star * beta * x[t-1-12]
		u[t] <- y.bc[t]-m[t]
	y.p1[t]<- ifelse((y.bc.p1[t]*l1+1)<0,0-l2, (pow((y.bc.p1[t]*l1+1),(1/l1)))-l2) 
	}
}
write.model(modelG310100, con = "modelG310100.txt")

N<-408
w<-16
parameters<-c("tau", "phi", "phi.star", paste("y.p1[",w+1,":",N,"]",sep="")) 

#Box-Cox transformation of malaria case data
l1<-0.249149
l2<-0.02507
y.bc<-(((y+l2)^l1)-1)/l1

data<-list(lag=15, N=N, p=3, y.bc= y.bc[1:N], x=x[1:N]/1000, l1=0.25, l2=0.02507)

#estimation:
rnorm(1)
inits<- list(list(tau=1, r.phi=0.1, r.phi.star=0.1), list(tau=0.5, r.phi=0.5, r.phi.star=0.1), list(tau=2, r.phi=0.9, r.phi.star=0.1))

ptm <- proc.time()
jags.output.modelG310100 <- jags(data= data, inits, parameters, model.file= "modelG310100.txt",
    n.iter=11000, n.burnin=1000, n.thin=1, n.chains=3)
proc.time() - ptm
alarm()

#module glm loaded
#Compiling model graph
#   Resolving undeclared variables
#   Allocating nodes
#   Graph Size: 13876
#
#
#  user  system elapsed 
# 322.95    3.20  328.69

print(jags.output.modelG310100, digits=2)
#Inference for Bugs model at "modelG310100.txt", fit using jags,
# 3 chains, each with 11000 iterations (first 1000 discarded)
# n.sims = 30000 iterations saved
#          mu.vect sd.vect    2.5%     25%     50%     75%   97.5% Rhat n.eff
#phi[1]       0.00    0.00    0.00    0.00    0.00    0.00    0.00    1     1
#phi[2]       0.00    0.00    0.00    0.00    0.00    0.00    0.00    1     1
#phi[3]      -0.22    0.05   -0.32   -0.25   -0.22   -0.19   -0.12    1 30000
#phi.star     0.10    0.05   -0.01    0.06    0.10    0.13    0.20    1  5100
#tau          0.30    0.02    0.26    0.29    0.30    0.31    0.34    1 20000
#y.p1[17]    68.99   44.78   12.27   37.01   59.03   90.24  181.21    1 30000
#deviance  1584.52    2.46 1581.71 1582.73 1583.87 1585.65 1590.84    1  6600
#
#For each parameter, n.eff is a crude measure of effective sample size,
#and Rhat is the potential scale reduction factor (at convergence, Rhat=1).
#
#DIC info (using the rule, pD = var(deviance)/2)
#pD = 3.0 and DIC = 1587.6
#DIC is an estimate of expected predictive error (lower deviance is better).

save(jags.output.modelG310100, file= "outmodelG3101001000.Rdata")
#load("outmodelG3101001000.Rdata")

res.mcmc<-as.mcmc(jags.output.modelG310100)
res.mcmc.sel<-res.mcmc[][,8:9]

res.mcmc.sel<-res.mcmc[][,12:13]
res.list<-mcmc.list(res.mcmc.sel[[1]],res.mcmc.sel[[2]],res.mcmc.sel[[2]])
gelman.diag(res.list)
gelman.plot(res.list)

re<-(jags.output.modelG310100$BUGSoutput$median$y.p1[1:(N-w)]-y[(w+1):N])/ (y[(w+1):N]+1)
mean(abs(re))
#[1] 0.423446

loop.rpv<-function(n, y, output){
	y.p1<- output$BUGSoutput$sims.list$y.p1[,(n)]
	F<-ecdf(y.p1)
	rpv<- F(y[(n+w)])
}

rpv.modelG310100 <- apply(as.matrix(1: (N-w)), MARGIN = 1, loop.rpv, y= y, output= jags.output.modelG310100)

plot(ecdf(rpv.modelG310100), verticals=TRUE, do.p = FALSE, xlab = "Residual probability", ylab = "Cumulative distribution of cumulative probabilities", main = NULL, col.01line = NULL)
lines(c(0,1),c(0,1), col=8)

loop.ptest<-function(n, N){
	test<-binom.test(n, N, n/N)
	low <-test$conf.int[1]
	high <-test$conf.int[2]
	res<-c(low, high)
	names(res)<-c("low", "high")
	res
}
bounds<- as.data.frame(t(apply(as.matrix(4: (N-w-4)), MARGIN = 1, loop.ptest, N=(N-16))))

z<-(4: (N-w-4))/(N-w)
lines(z,bounds$low, lty=2, col=8)
lines(z,bounds$high, lty=2, col=8)


len<-length(rpv.modelG310100)
length(rpv.modelG310100[c(len-49): len])
plot(ecdf(rpv.modelG310100[c(len-49): len]), verticals=TRUE, do.p = FALSE, xlab = "Residual probability", ylab = "Cumulative distribution of cumulative probabilities", main = NULL, col.01line = NULL)
lines(c(0,1),c(0,1), col=8)
bounds<- as.data.frame(t(apply(as.matrix(1: (50-1)), MARGIN = 1, loop.ptest, N=50)))
z<-(1: (50-1))/(50)
lines(z,bounds$low, lty=2, col=8)
lines(z,bounds$high, lty=2, col=8)


##Plot both the negative binomial and the Gaussian model in the same graph.
plot(ecdf(rrpv.model310100), verticals=TRUE, do.p = FALSE, xlab = "(Randomized) residual probability", ylab = "Cumulative distribution of (randomized) cumulative probabilities", main = NULL, col.01line = NULL)
lines(c(0,1),c(0,1), col=8)

loop.ptest<-function(n, N){
	test<-binom.test(n, N, n/N)
	low <-test$conf.int[1]
	high <-test$conf.int[2]
	res<-c(low, high)
	names(res)<-c("low", "high")
res
}
bounds<- as.data.frame(t(apply(as.matrix(4: (N-w-4)), MARGIN = 1, loop.ptest, N=(N-16))))
z<-(4: (N-w-4))/(N-w)
lines(z,bounds$low, lty=2, col=8)
lines(z,bounds$high, lty=2, col=8)

plot.stepfun(rpv.modelG310100, col.hor=2, col.vert=2, do.points=FALSE, add=TRUE)

##Figure 2A in main text


len<-length(rrpv.model310100)
length(rrpv.model310100[c(len-49): len])

##Last 50 only
plot(ecdf(rrpv.model310100[c(len-49): len]), verticals=TRUE, do.p = FALSE, xlab = "(Randomized) residual probability", ylab = "Cumulative distribution of (randomized) cumulative probabilities", main = NULL, col.01line = NULL)
lines(c(0,1),c(0,1), col=8)
bounds<- as.data.frame(t(apply(as.matrix(1: (50-1)), MARGIN = 1, loop.ptest, N=50)))
z<-(1: (50-1))/(50)
lines(z,bounds$low, lty=2, col=8)
lines(z,bounds$high, lty=2, col=8)

plot.stepfun(rpv.modelG310100[c(len-49): len], col.hor=2, col.vert=2, do.points=FALSE, add=TRUE)


tiff(filename = "Figure2AB.tif",
     width = 12.35, height = 23.35, units = "cm", pointsize = 8,
     compression = c("lzw"),
     bg = "white", res = 300, family = "Arial", restoreConsole = TRUE,
     type = c("cairo"), antialias=c("subpixel"))
op <- par(mfrow=c(2,1), mai=c(0.2,0,0,0),omi=c(0.02778+0.4, 0.02778+0.6, 0.02778, 0.02778), mgp=c(2.5,1,0), cex.lab=1.4)
plot(ecdf(rrpv.model310100), verticals=TRUE, do.p = FALSE, xlab = "", ylab = "", main = NULL, col.01line = NULL, col="0", xaxt="n", yaxt="n")
axis(1, labels=FALSE)
axis(2)
lines(c(0,1),c(0,1), col=8)
z<-(4: (N-w-4))/(N-w)
bounds<- as.data.frame(t(apply(as.matrix(4: (N-w-4)), MARGIN = 1, loop.ptest, N=(N-16))))
lines(z,bounds$low, lty=2, col=8)
lines(z,bounds$high, lty=2, col=8)
plot.stepfun(rrpv.model310100, col.hor=1, col.vert=1, do.points=FALSE, add=TRUE)
plot.stepfun(rpv.modelG310100, col.hor=2, col.vert=2, do.points=FALSE, add=TRUE)
text(0, 0.9,"A", font=2, cex=1.5)


plot(ecdf(rrpv.model310100[c(len-49): len]), verticals=TRUE, do.p = FALSE, xlab = "", ylab = "", main = NULL, col.01line = NULL, col=0, yaxt="n")
axis(2)
lines(c(0,1),c(0,1), col=8)
bounds<- as.data.frame(t(apply(as.matrix(1: (50-1)), MARGIN = 1, loop.ptest, N=50)))
z<-(1: (50-1))/(50)
lines(z,bounds$low, lty=2, col=8)
lines(z,bounds$high, lty=2, col=8)
plot.stepfun(rrpv.model310100[c(len-49): len], col.hor=1, col.vert=1, do.points=FALSE, add=TRUE)
plot.stepfun(rpv.modelG310100[c(len-49): len], col.hor=2, col.vert=2, do.points=FALSE, add=TRUE)
text(0, 0.9,"B", font=2, cex=1.5)

mtext("(Randomized) residual probability", 1, outer=TRUE, line=1.9, cex=1.4)
mtext("Cumulative distribution of (randomized) residual probabilities", 2, outer=TRUE, line=3, cex=1.4) 

par(op)
dev.off()


 

nrqr<-qnorm(pmax(0.00001, pmin(0.99999, rpv.modelG310100)))

nrqr.ts<-ts(nrqr, start=c(1973,5), freq=12)
plot(nrqr.ts, type = "p")

plot(density(nrqr))

qqnorm(nrqr)
qqline(nrqr)

plot(diff(log(tsy+1)))

lmres<-lm(nrqr[2: (N-w)]~ diff(log(y[(w+1):N]+1)))
summary(lmres)

plot(diff(log(y[(w+1):N]+1)), nrqr[2:(N-w)])
lines(c(-3,3), c(lmres$coef[1]+ -3*lmres$coef[2], lmres$coef[1]+ 3*lmres$coef[2]))

acf(nrqr, lag.max=36)


pacf(nrqr, lag.max=36)

bt<-Box.test(nrqr, lag = 24, type = "Ljung-Box")
bt$statistic
#X-squared 
# 18.57597
pchisq(q= bt$statistic[[1]], df=24-1, lower.tail=FALSE)
#[1] 0.7255423


#Identity link version of negative binomial model:

library(R2jags)
model310100id<-function(){
	##Priors
#	beta~dnorm(0,0.001)
	beta<-0 #use this option in a model without external variable
	r~dgamma(0.01,0.01)
#	phi.star<-0
	#phi.star~dunif(-0.999999, 0.999999)
	r.phi.star ~dbeta(1,1)
	phi.star <-2*r.phi.star -1
	phi[1]<-0
	phi[2]<-0
	#phi[3]~dunif(-0.999999, 0.999999)
	r.phi ~dbeta(1,1)
	phi[3]<-2*r.phi -1

	##Ljung-Box test
	Chlag ~ dchisqr(df)
	df<-lag-2
	##Ljung-Box test on Pearson residuals e
	for(k in 1: lag){
		for(t in (k + 1 +3+1+12): (N)){
			e.lag[k,t] <- (y[t-k]-lambda[t-k])/sqrt(V[t-k])
			p1[k,t] <- e[t]*e.lag[k,t]
		}
		auto[k] <- sum(p1[k,(k + 1 +3+1+12):(N)])/sum(e2[(1 +3+1+12) : (N)])
		auto2[k] <- auto[k]*auto[k]
		auto2div[k] <- auto2[k]/(N-k)
	}
	LB <-  N*(N+2)*sum(auto2div[])
	P.LB <- step(LB-Chlag)
	
	##Ljung-Box test on relative residuals eta
	for(k in 1: lag){
		for(t in (k + 1 +3+1+12): (N)){
			eta.lag[k,t] <- (y[t-k]-lambda[t-k])/sqrt(V[t-k])
			p1.eta[k,t] <- eta[t]*eta.lag[k,t]
		}
		auto.eta[k] <- sum(p1.eta[k,(k + 1 +3+1+12):(N)])/sum(eta2[(1 +3+1+12) : (N)])
		auto.eta2[k] <- auto.eta[k]*auto.eta[k]
		auto.eta2div[k] <- auto.eta2[k]/(N-k)
	}
	LB.eta <-  N*(N+2)*sum(auto.eta2div[])
	P.LB.eta <- step(LB.eta-Chlag)
		
	##Predictive check on overdispersion
	Diagobs <- pow(sd(y[(1 +3+1+12) : (N)]),2)/mean(y[(1 +3+1+12) : (N)])
	Diagnew <- pow(sd(y.p1[(1 +3+1+12) : (N)]),2)/mean(y.p1[(1 +3+1+12) : (N)])
	PrCh <- step(Diagnew-Diagobs)
	
	##Deviance
	Dev <- -2*sum(LL[(3+1+12+1) : (N)])
	
	##Predicting preseries latent observations
	for (t in 1: (12+3+1)){
		u[t] <- 0
	}
	
	##likelihood
	for (t in (12+3+1+1):N){
		y[t]~dnegbin(pr[t],r)
		y.p1[t]~dnegbin(pr[t],r)
		pr[t]<-r/(r+lambda[t])
		#lambda[t]<-exp(m[t])
		lambda[t]<-max(0.000000001,m[t])

		m[t] <- beta * x[t] 
+((y[t-1])) 
-beta * x[t-1] 
+ sum(AR[ ,t]) + sum(ARSAR[ ,t]) + SAR[t]
		for (k in 1:p){
			AR[k,t]<-
			 (phi[k])*((y[t-k]))
			-phi[k] * beta * x[t-k] 
			-(phi[k])*((y[t-k-1]))
			+phi[k] * beta * x[t-k-1]
			ARSAR[k,t]<- 
			-(phi.star)*(phi[k])*((y[t-k-12]))
			+phi.star*phi[k] * beta * x[t-k-12] 
			+(phi.star) *(phi[k])*((y[t-k-1-12])) 
			-phi.star*phi[k] * beta * x[t-k-1-12] 
		}
		SAR[t]<-
		 (phi.star)*((y[t-12])) 
		-phi.star * beta * x[t-12]
		-(phi.star)*((y[t-1-12]))
		+ phi.star * beta * x[t-1-12]
		u[t] <- ((y[t])-lambda[t])
		V[t] <- lambda[t]+ lambda[t]*lambda[t]/r
		e[t] <- (y[t]-lambda[t])/sqrt(V[t])
		e2[t] <- e[t]*e[t]
		eta[t] <- (y[t]-lambda[t])/(y[t]+1)
		eta2[t] <- eta[t]*eta[t]
		LL[t] <-r*log(pr[t])+y[t]*log(1-pr[t])+loggam(y[t]+r)- loggam(y[t]+1) - loggam(r)
	}
##prediction out of series(activate in case N<M) 
	#for (t in (N+1):M){
#		y.p1[t]~dnegbin(pr[t],r)
#		pr[t]<-r/(r+lambda[t])
#		lambda[t]<-max(0.000000001,m[t])
#		m[t] <- beta * x[t] 
#			+((y[t-1])) 
#			-beta * x[t-1] 
#			+ sum(AR[ ,t]) + sum(ARSAR[ ,t]) + SAR[t]
#		for (k in 1:p){
#			AR[k,t]<-
#			 (phi[k])*((y[t-k]))
#			-phi[k] * beta * x[t-k] 
#			-(phi[k])*((y[t-k-1]))
#			+phi[k] * beta * x[t-k-1]
#			ARSAR[k,t]<- 
#			-(phi.star)*(phi[k])*((y[t-k-12]))
#			+phi.star*phi[k] * beta * x[t-k-12] 
#			+(phi.star) *(phi[k])*((y[t-k-1-12])) 
#			-phi.star*phi[k] * beta * x[t-k-1-12] 
#		}
#		SAR[t]<-
#		 (phi.star)*((y[t-12])) 
#		-phi.star * beta * x[t-12]
#		-(phi.star)*((y[t-1-12]))
#		+ phi.star * beta * x[t-1-12]
#		u[t] <- ((y[t])-lambda[t])
#}
}
write.model(model310100id, con = "model310100id.txt")

w<-16
N<-408
parameters<-c("r", "phi", "phi.star", paste("y.p1[",w+1,":",N,"]",sep=""), "LB", "Chlag", "P.LB", "Dev", "Diagnew", "PrCh", "P.LB.eta", "LB.eta") 
data<-list(lag=15, N=N, p=3, y= y[1:N], x=x[1:N]/1000)

#In case of predicting on out of sample part of the series
#M<-408
#N<-204
#parameters<-c("r", "phi", "phi.star", paste("y.p1[",w+1,":",M,"]",sep=""), "LB", "Chlag", "P.LB", "Dev", "Diagnew", "PrCh", "P.LB.eta", "LB.eta") 
#data<-list(lag=15, N=N, M=M, p=3, y= y[1:M], x=x[1:M]/1000)

#estimation:
rnorm(1)
inits<- list(list(r=10, r.phi=0.1, r.phi.star=0.1), list(r=5, r.phi=0.5, r.phi.star=0.1), list(r=2, r.phi=0.9, r.phi.star=0.1))

ptm <- proc.time()
jags.output.model310100id <- jags(data= data, inits, parameters, model.file= "model310100id.txt",
    n.iter=11000, n.burnin=1000, n.thin=1, n.chains=3)
proc.time() - ptm
alarm()


ptm <- proc.time()
jags.output.model310100id <- jags(data= data, inits, parameters, model.file= "model310100id.txt",
    n.iter=11000, n.burnin=1000, n.thin=1, n.chains=3)
proc.time() - ptm
alarm()

print(jags.output.model310100id, digits=2)

#Inference for Bugs model at "model310100id.txt", fit using jags,
# 3 chains, each with 11000 iterations (first 1000 discarded)
# n.sims = 30000 iterations saved
#               mu.vect     sd.vect    2.5%     25%     50%          75%        97.5% Rhat n.eff
#Chlag            13.00 5.11000e+00    5.01    9.32   12.35        15.95        24.76 1.00 30000
#Dev            4455.74 3.28500e+01 4420.51 4422.52 4432.87      4488.03      4493.47 2.67     4
#Diagnew         607.56 1.24710e+02  419.03  520.44  587.86       674.65       899.25 1.01   460
#LB               59.07 7.51000e+00   47.47   51.97   57.60        66.41        66.41 2.45     4
#LB.eta    218045799.66 2.19545e+08  270.88  286.39  324.43 437890099.69 468334380.31 2.69     4
#P.LB              1.00 0.00000e+00    1.00    1.00    1.00         1.00         1.00 1.00     1
#P.LB.eta          1.00 0.00000e+00    1.00    1.00    1.00         1.00         1.00 1.00     1
#PrCh              0.95 2.20000e-01    0.00    1.00    1.00         1.00         1.00 1.01  1500
#phi[1]            0.00 0.00000e+00    0.00    0.00    0.00         0.00         0.00 1.00     1
#phi[2]            0.00 0.00000e+00    0.00    0.00    0.00         0.00         0.00 1.00     1
#phi[3]           -0.05 6.00000e-02   -0.13   -0.11    0.00         0.01         0.01 2.55     4
#phi.star          0.01 4.00000e-02   -0.06   -0.03    0.03         0.05         0.06 2.37     4
#r                 4.10 3.30000e-01    3.47    3.87    4.09         4.32         4.78 1.01   460
#y.p1[17]         51.63 2.66400e+01   13.00   32.00   47.00        66.00       115.00 1.00  4700
#y.p1[408]         3.94 2.77000e+00    0.00    2.00    3.00         5.00        11.00 1.00  2300
#deviance       4455.74 3.28500e+01 4420.51 4422.52 4432.87      4488.03      4493.47 2.67     4

#For each parameter, n.eff is a crude measure of effective sample size,
and Rhat is the potential scale reduction factor (at convergence, Rhat=1).

#DIC info (using the rule, pD = var(deviance)/2)
#pD = 182.1 and DIC = 4637.8
#DIC is an estimate of expected predictive error (lower deviance is better).
 
save(jags.output.model310100id, file= "outmodel3101001000id.Rdata")
#load("outmodel3101001000id.Rdata")

res.mcmc<-as.mcmc(jags.output.model310100id)
res.mcmc.sel<-res.mcmc[][,8:9]

res.mcmc.sel<-res.mcmc[][,12:13]
res.list<-mcmc.list(res.mcmc.sel[[1]],res.mcmc.sel[[2]],res.mcmc.sel[[3]])
gelman.diag(res.list)

Potential scale reduction factors:

         Point est. Upper C.I.
phi[3]        10.22       43.4
phi.star       5.94       15.6

Multivariate psrf

8.51
re<-(jags.output.model310100id$BUGSoutput$median$y.p1[1:(N-w)]-y[(w+1):N])/ (y[(w+1):N]+1)
mean(abs(re))

#[1] 0.4054151

model310100idous<-function(){
	##Priors
#	beta~dnorm(0,0.001)
	beta<-0 #use this option in a model without external variable
	r~dgamma(0.01,0.01)
#	phi.star<-0
	#phi.star~dunif(-0.999999, 0.999999)
	r.phi.star ~dbeta(1,1)
	phi.star <-2*r.phi.star -1
	phi[1]<-0
	phi[2]<-0
	#phi[3]~dunif(-0.999999, 0.999999)
	r.phi ~dbeta(1,1)
	phi[3]<-2*r.phi -1

	##Ljung-Box test
	Chlag ~ dchisqr(df)
	df<-lag-2
	##Ljung-Box test on Pearson residuals e
	for(k in 1: lag){
		for(t in (k + 1 +3+1+12): (N)){
			e.lag[k,t] <- (y[t-k]-lambda[t-k])/sqrt(V[t-k])
			p1[k,t] <- e[t]*e.lag[k,t]
		}
		auto[k] <- sum(p1[k,(k + 1 +3+1+12):(N)])/sum(e2[(1 +3+1+12) : (N)])
		auto2[k] <- auto[k]*auto[k]
		auto2div[k] <- auto2[k]/(N-k)
	}
	LB <-  N*(N+2)*sum(auto2div[])
	P.LB <- step(LB-Chlag)
	
	##Ljung-Box test on relative residuals eta
	for(k in 1: lag){
		for(t in (k + 1 +3+1+12): (N)){
			eta.lag[k,t] <- (y[t-k]-lambda[t-k])/sqrt(V[t-k])
			p1.eta[k,t] <- eta[t]*eta.lag[k,t]
		}
		auto.eta[k] <- sum(p1.eta[k,(k + 1 +3+1+12):(N)])/sum(eta2[(1 +3+1+12) : (N)])
		auto.eta2[k] <- auto.eta[k]*auto.eta[k]
		auto.eta2div[k] <- auto.eta2[k]/(N-k)
	}
	LB.eta <-  N*(N+2)*sum(auto.eta2div[])
	P.LB.eta <- step(LB.eta-Chlag)
		
	##Predictive check on overdispersion
	Diagobs <- pow(sd(y[(1 +3+1+12) : (N)]),2)/mean(y[(1 +3+1+12) : (N)])
	Diagnew <- pow(sd(y.p1[(1 +3+1+12) : (N)]),2)/mean(y.p1[(1 +3+1+12) : (N)])
	PrCh <- step(Diagnew-Diagobs)
	
	##Deviance
	Dev <- -2*sum(LL[(3+1+12+1) : (N)])
	
	##Predicting preseries latent observations
	for (t in 1: (12+3+1)){
		u[t] <- 0
	}
	
	##likelihood
	for (t in (12+3+1+1):N){
		y[t]~dnegbin(pr[t],r)
		y.p1[t]~dnegbin(pr[t],r)
		pr[t]<-r/(r+lambda[t])
		#lambda[t]<-exp(m[t])
		lambda[t]<-max(0.000000001,m[t])

		m[t] <- beta * x[t] 
+((y[t-1])) 
-beta * x[t-1] 
+ sum(AR[ ,t]) + sum(ARSAR[ ,t]) + SAR[t]
		for (k in 1:p){
			AR[k,t]<-
			 (phi[k])*((y[t-k]))
			-phi[k] * beta * x[t-k] 
			-(phi[k])*((y[t-k-1]))
			+phi[k] * beta * x[t-k-1]
			ARSAR[k,t]<- 
			-(phi.star)*(phi[k])*((y[t-k-12]))
			+phi.star*phi[k] * beta * x[t-k-12] 
			+(phi.star) *(phi[k])*((y[t-k-1-12])) 
			-phi.star*phi[k] * beta * x[t-k-1-12] 
		}
		SAR[t]<-
		 (phi.star)*((y[t-12])) 
		-phi.star * beta * x[t-12]
		-(phi.star)*((y[t-1-12]))
		+ phi.star * beta * x[t-1-12]
		u[t] <- ((y[t])-lambda[t])
		V[t] <- lambda[t]+ lambda[t]*lambda[t]/r
		e[t] <- (y[t]-lambda[t])/sqrt(V[t])
		e2[t] <- e[t]*e[t]
		eta[t] <- (y[t]-lambda[t])/(y[t]+1)
		eta2[t] <- eta[t]*eta[t]
		LL[t] <-r*log(pr[t])+y[t]*log(1-pr[t])+loggam(y[t]+r)- loggam(y[t]+1) - loggam(r)
	}
##prediction out of series(activate in case N<M) 
	for (t in (N+1):M){
		y.p1[t]~dnegbin(pr[t],r)
		pr[t]<-r/(r+lambda[t])
		lambda[t]<-max(0.000000001,m[t])
		m[t] <- beta * x[t] 
			+((y[t-1])) 
			-beta * x[t-1] 
			+ sum(AR[ ,t]) + sum(ARSAR[ ,t]) + SAR[t]
		for (k in 1:p){
			AR[k,t]<-
			 (phi[k])*((y[t-k]))
			-phi[k] * beta * x[t-k] 
			-(phi[k])*((y[t-k-1]))
			+phi[k] * beta * x[t-k-1]
			ARSAR[k,t]<- 
			-(phi.star)*(phi[k])*((y[t-k-12]))
			+phi.star*phi[k] * beta * x[t-k-12] 
			+(phi.star) *(phi[k])*((y[t-k-1-12])) 
			-phi.star*phi[k] * beta * x[t-k-1-12] 
		}
		SAR[t]<-
		 (phi.star)*((y[t-12])) 
		-phi.star * beta * x[t-12]
		-(phi.star)*((y[t-1-12]))
		+ phi.star * beta * x[t-1-12]
		u[t] <- ((y[t])-lambda[t])
 }
}
write.model(model310100idous, con = "model310100idous.txt")

w<-16
#In case of predicting on out of sample part of the series
M<-408
N<-204
parameters<-c("r", "phi", "phi.star", paste("y.p1[",w+1,":",M,"]",sep=""), "LB", "Chlag", "P.LB", "Dev", "Diagnew", "PrCh", "P.LB.eta", "LB.eta") 
data<-list(lag=15, N=N, M=M, p=3, y= y[1:M], x=x[1:M]/1000)

#estimation:
rnorm(1)
inits<- list(list(r=10, r.phi=0.1, r.phi.star=0.1), list(r=5, r.phi=0.5, r.phi.star=0.1), list(r=2, r.phi=0.9, r.phi.star=0.1))

ptm <- proc.time()
jags.output.model310100idous <- jags(data= data, inits, parameters, model.file= "model310100idous.txt",
    n.iter=11000, n.burnin=1000, n.thin=1, n.chains=3)
proc.time() - ptm
alarm()

print(jags.output.model310100idous, digits=2)


#Inference for Bugs model at "model310100idous.txt", fit using jags,
# 3 chains, each with 11000 iterations (first 1000 discarded)
# n.sims = 30000 iterations saved
#          mu.vect sd.vect    2.5%     25%     50%     75%   97.5% Rhat n.eff
#Chlag       13.04    5.11    4.99    9.31   12.36   16.06   24.76 1.00 26000
#Dev       2263.62    2.43 2260.90 2261.86 2262.97 2264.68 2270.01 1.00 30000
#Diagnew    797.38  219.46  484.10  644.64  762.14  904.73 1331.57 1.00 30000
#LB          42.95    0.91   41.03   42.35   43.05   43.60   44.58 1.00 28000
#LB.eta     165.07   43.51   98.12  133.94  159.12  189.09  265.94 1.00  6200
#P.LB         1.00    0.01    1.00    1.00    1.00    1.00    1.00 1.29 30000
#P.LB.eta     1.00    0.00    1.00    1.00    1.00    1.00    1.00 1.00     1
#PrCh         0.90    0.30    0.00    1.00    1.00    1.00    1.00 1.00 30000
#phi[1]       0.00    0.00    0.00    0.00    0.00    0.00    0.00 1.00     1
#phi[2]       0.00    0.00    0.00    0.00    0.00    0.00    0.00 1.00     1
#phi[3]      -0.11    0.02   -0.14   -0.12   -0.11   -0.09   -0.07 1.00 15000
#phi.star    -0.03    0.02   -0.08   -0.05   -0.03   -0.02    0.01 1.00  6300
#r            3.40    0.38    2.71    3.14    3.39    3.65    4.19 1.00  6600
#y.p1[17]    51.81   29.21   11.00   30.00   47.00   68.00  123.00 1.00 21000
#y.p1[408]    3.81    2.86    0.00    2.00    3.00    5.00   11.00 1.00 22000
#deviance  2263.62    2.43 2260.90 2261.86 2262.97 2264.68 2270.01 1.00 30000
#
#For each parameter, n.eff is a crude measure of effective sample size,
#and Rhat is the potential scale reduction factor (at convergence, Rhat=1).
#
#DIC info (using the rule, pD = var(deviance)/2)
#pD = 3.0 and DIC = 2266.6
#DIC is an estimate of expected predictive error (lower deviance is better).
 
save(jags.output.model310100idous, file= "outmodel3101001000idous.Rdata")
#load("outmodel3101001000idous.Rdata")

res.mcmc<-as.mcmc(jags.output.model310100idous)
res.mcmc.sel<-res.mcmc[][,8:9]

res.mcmc.sel<-res.mcmc[][,12:13]
res.list<-mcmc.list(res.mcmc.sel[[1]],res.mcmc.sel[[2]],res.mcmc.sel[[3]])
gelman.diag(res.list)

#Potential scale reduction factors:
#
#         Point est. Upper C.I.
#phi[3]        10.22       43.4
#phi.star       5.94       15.6
#
#Multivariate psrf
#
#8.51

#In case of out of sample prediction on the second half of the series
re<-(jags.output.model310100idous$BUGSoutput$median$y.p1[(N-w+1):(M-w)]-y[(N+1):M])/ (y[(N+1):M]+1)
mean(abs(re))
#[1] 0.3969862

hist(res.mcmc[][,14])


TODO: GENERATE OUTLINE HISTOGRAMS FOR BOTH POISSON AND GAUSSIAN PREDICTIONS (IN DIFFERENT COLOUR) FOR THE LAST 10 OBSERVATIONS, AND ADD THE OBSERVED VALUE.
THE GAUSSIAN ONES MAY HAVE TO BE ROUNDED TO THE NEAREST INTEGER VALUE? OR MAYBE THAT IS DONE AUTOMATICALLY INSIDE THE HISTOGRAM.

y <- c(62,59,70,42,85,52,97,98,85,54,92,134,101,59,46,49,56,82,106,178,140,162,204,390,442,407,607,374,254,275,283,223,145,186,166,221,279,221,170,105,141,296,298,226,169,239,177,145,141,155,110,178,191,573,2462,915,1005,507,530,279,965,668,818,463,392,297,379,508,333,393,401,213,206,90,158,110,125,132,86,70,105,183,152,72,97,103,171,107,251,239,401,149,151,134,96,82,73,87,91,64,62,51,48,63,64,35,38,57,37,26,25,35,30,35,44,20,12,3,8,6,1,9,9,26,63,39,10,3,5,0,13,29,35,40,79,187,1402,2507,2008,240,476,711,653,710,603,826,770,625,390,326,279,301,231,155,137,170,132,94,107,51,46,68,95,89,104,103,127,149,126,132,123,72,134,159,194,335,262,389,186,408,751,703,973,1287,1864,1771,1536,923,1778,1039,895,496,657,317,395,298,287,262,202,171,134,103,138,92,139,80,66,124,399,489,188,190,117,85,113,99,156,59,253,452,405,366,196,193,111,204,381,721,1506,580,315,224,164,212,176,211,223,268,167,264,530,333,237,194,631,989,684,439,342,444,512,515,675,425,451,424,467,393,295,326,350,377,473,638,703,543,515,356,323,404,397,380,379,404,310,305,352,286,298,149,154,130,111,87,105,121,150,140,178,201,207,215,206,543,1163,724,339,246,197,199,244,313,239,247,223,352,406,248,228,119,87,103,95,86,113,105,97,108,224,251,213,111,127,98,104,99,82,55,87,74,82,70,76,133,141,204,208,148,95,57,90,149,197,310,282,150,100,63,84,55,38,28,29,27,31,24,19,24,34,32,30,25,17,16,14,21,20,11,12,8,11,7,10,8,5,4,6,5,2,5,3,2,2,1,2,1,2,3,1,1,15,4,4,2,1,1,2,2,0,1,3,20,19,0,2,1,4,2,5,1,0,0,1,2,1,0,0,1,0,0)


load("Z:/Projects/Active/Programme MCN/6. Manuscripts/Submitted/Briet_GSARIMA/DATA/outmodel3101001000.Rdata")

load("Z:/Projects/Active/Programme MCN/6. Manuscripts/Submitted/Briet_GSARIMA/DATA/outmodelG3101001000.Rdata")

output.NB<- jags.output.model310100

output.G<- jags.output.modelG310100


lastobs<-12
w<-16
N<-408

#max height = height = 23.35

tiff(filename = "Figure7.tif",
     width = 8.3, height = 18, units = "cm", pointsize = 8,
     compression = c("lzw"),
     bg = "white", res = 300, family = "Arial", restoreConsole = TRUE,
     type = c("cairo"), antialias=c("subpixel"))
#op <- par(mar=c(3.6,3.6,0,0),omi=c(0.02778, 0.02778, 0.02778, 0.02778), mgp=c(2.5,1,0), cex.lab=1.4)


#pdf(file = "FigureS5.pdf",     width = 6.83, height = 6.83, pointsize = 8,     bg = "white")

op <- par(mfrow=c(lastobs,1), mar=c(0.8,2.5,0,0),oma=c(3.5, 3.5, 0.5, 0.5), mgp=c(2.5,1,0), cex.lab=1.4)


for(i in 1:lastobs){

outobs.NB<-output.NB$BUGSoutput$sims.list$y.p1[,N-w-(lastobs-i)]
outobs.G<-output.G$BUGSoutput$sims.list$y.p1[,N-w-(lastobs-i)]


histobs.NB1<-hist(outobs.NB, breaks=c(0:1000)-0.5, plot=FALSE)
histobs.G1<-hist(outobs.G, breaks=c(0:1000)-0.5, plot=FALSE)
histmax<-max(max(histobs.NB1$density), max(histobs.G1$density))


histobs.NB<-hist(outobs.NB, freq=FALSE, xlim=c(-0.5,100), ylim=c(0,histmax), breaks=c(0:1000)-0.5, col=NULL, border=0, xaxt="n", yaxt="n", main=NULL, xlab=NULL, ylab=NULL)
axis(2, at=c(0, round(histmax,2)), labels= c(0, round(histmax,2)), las=1)

density.dub.NB<-c(0, rep(histobs.NB$density, each=2)[])
breaks.dub.NB<-rep(histobs.NB$breaks, each=2)[1:(2*length(histobs.NB$breaks)-1)]


histobs.G<-hist(outobs.G, breaks=c(0:1000)-0.5, plot=FALSE)

density.dub.G<-c(0, rep(histobs.G$density, each=2)[])
breaks.dub.G<-rep(histobs.G$breaks, each=2)[1:(2*length(histobs.G$breaks)-1)]

clip(-1, 101, 0, histmax*1.05)
lines(breaks.dub.G, density.dub.G, col=2, lwd=1.5)
lines(breaks.dub.NB, density.dub.NB, col=1, lwd=1.5)

clip(usr[1], usr[2], usr[3], usr[4])

points(y[N-(lastobs-i)],0, col=4, pch=19)
axis(1, labels=FALSE)

MONTH<-c("Jan","Feb","Mar","Apr","May","Jun","Jul","Aug","Sep","Oct","Nov","Dec")

text(80, 0.5* histmax, paste(MONTH[trunc(1+12*time(tsy)[N-(lastobs-i)]%%.999999,0)], ", ", time(tsy)[N-(lastobs-i)]%/%1, sep=""))

}
axis(1)
mtext("Cases", side=1, line=2, outer=TRUE)
mtext("Density", side=2, line=2, outer=TRUE)

par(op)


dev.off()

system(paste(getOption("pdfviewer"), "FigureS5.pdf"))

Sys.setenv(R_GSCMD = "C:/Program Files/gs/gs9.04/bin/gswin64c.exe")
embedFonts(file= "FigureS5.pdf", outfile= "FigureS5e.pdf", format="pdfwrite")
system(paste(getOption("pdfviewer"), "FigureS5e.pdf"))
