# Supplementary material for: Generalized Seasonal Autoregressive Integrated Moving Average Models for Count Data with Application to Malaria Time Series with Low Case Numbers
Source: PLoS One. 2013 Jun 13;8(6):e65761. doi: 10.1371/journal.pone.0065761 (PMC3681978; doi:10.1371/journal.pone.0065761)
Supplement: Additional File S3 — R code for examples illustrating how plots of a cumulative distribution function of residual probability values, here called "C-R plots" can be used to estimate the appropriateness of the posterior predictive distributions (RTF) [file pone.0065761.s007.rtf]

Additional file S3
##Additional File S3, supporting information to Briët et al.: “Generalized seasonal autoregressive integrated moving average models for count data with application to malaria time series with low case numbers”
##This file contains computer code for use in the free software R [http://cran.r-project.org/]. It gives examples illustrating how plots of a cumulative distribution function of residual probability values, here called "C-R plots" can be used to assess the appropriateness of the posterior predictive distributions, and compares these for Poisson and Gaussian models fitted to Poisson data. For the code to run, it requires a few R packages to be installed. 

##Consider a process y~Poisson(exp(beta*x)).
##Suppose that in a lab, measurements are taken at three levels of a covariate x: for x=0.25 x=1, and x=4, n measurements each.

##n samples are taken
n<-100
beta<-0.5
##Suppose that the data generating process is unknown to the laboratory statistician:
yx025s<-rpois(n, lambda=exp(beta*0.25)) 
yx1s<-rpois(n, lambda=exp(beta*1)) 
yx4s<-rpois(n, lambda=exp(beta*4))
##

xs<-c(rep(0.25,n), rep(1,n), rep(4, n))
ys<-c(yx025s, yx1s, yx4s)
plot(xs,ys) #Note that many points are superimposed.
##Suppose now that the lab statistician correctly presumes the data is Poisson distributed, and tries the regression:
glm.result<-glm(ys~xs-1, family="poisson")
summary(glm.result)

beta.hat<-glm.result$coef
beta.hat

##How does (s)he assess that the Poisson model was the correct one?
##At each level of x, the shape of the distribution of y should match that of the theoretical distribution.
##The following are the histograms at each level of x:
hist(yx025s, 100) 
hist(yx1s, 100) 
hist(yx4s, 100) 
##Note that these distributions have different shapes.

##Generate histograms of the theoretical distributions by sampling many samples from these theoretical distributions: 
N<-100000
yx025<-rpois(N, lambda=exp(beta.hat*0.25)) 
yx1<-rpois(N, lambda=exp(beta.hat*1)) 
yx4<-rpois(N, lambda=exp(beta.hat*4))

##The following are the histograms of the theoretical distributions at each level of x:
hist(yx025, 100)
hist(yx1, 100) 
hist(yx4, 100) 
##These theoretical distributions also have different shapes, but appear to match the ones of the observations (at each level of x).

##One could use a Q-Q plot to establish whether the shapes of the distributions of the observations match their theoretical distribution shapes, but these Q-Q plots are difficult to read:


qqplot(x=yx025, y= yx025s)
lines(c(0:100), c(0:100), col=8, lty=3) #The diagonal

qqplot(x=yx1, y= yx1s)
lines(c(0:100), c(0:100), col=8, lty=3)

qqplot(x=yx4,y=yx4s)
lines(c(0:100), c(0:100), col=8, lty=3)

##Now, consider a plot of a cumulative distribution function of residual probability values, here called "C-R plots".
##Consider the theoretical cumulative distribution function at x=0.25, F, approximated by the N samples:
F<-ecdf(yx025)
plot(F)
##For each observed y value (in yx025s), calculate the residual probability value (RPV), which is the probability that, given the theoretical distribution of y values at x=0.25, a value less than or equal to the actually observed y value could have been found. Because of discontinuity in the theoretical cumulative distribution function (because these are count data and therefore integers), adjust for this by calculating the randomized residual probability value (RRPV) instead. Each RRPV should occur approximately equally often in the sample: e.g. 10% of observations should have a (randomized) residual probability value of less than 0.1.

yx025s.rrpv<-vector(length=n)
for(i in 1:n){
yx025s.rrpv[i]<- runif(1,F(yx025s[i]-1), F(yx025s[i]))
}
hist(yx025s.rrpv, 20) #This should indicate a approximately uniform distribution.

##A plot of the empirical cumulative distribution function (ECDF) of the population of RRPVs should approximate a straight line on the diagonal.
plot(ecdf(c(yx025s.rrpv)), xlim=c(0,1), ylim=c(0,1), col.01line=0, do.points=FALSE, verticals=TRUE)
lines(c(0,1),c(0,1), col=8)
#Construct 95% confidence boundary lines around the diagonal (these depend on the number of observations n), using an exact binomial test.
loop.ptest<-function(n, N){
	test<-binom.test(n, N, n/N)
	low <-test$conf.int[1]
	high <-test$conf.int[2]
	res<-c(low, high)
	names(res)<-c("low", "high")
	res
}
bounds<- as.data.frame(t(apply(as.matrix(1: (n-1)), MARGIN = 1, loop.ptest, N=n)))
z<-(1: (n-1))/(n)
lines(z,bounds$low, lty=2, col=8)
lines(z,bounds$high, lty=2, col=8)


##Do the same for the measurements at x=1
F<-ecdf(yx1)
plot(F)
yx1s.rrpv<-vector(length=n)
F<-ecdf(yx1)
for(i in 1:n){
yx1s.rrpv[i]<- runif(1,F(yx1s[i]-1), F(yx1s[i]))
}

hist(yx1s.rrpv, 10)
plot(ecdf(c(yx1s.rrpv)), xlim=c(0,1), ylim=c(0,1), col.01line=0, do.points=FALSE, verticals=TRUE)
lines(c(0,1),c(0,1), col=8)
lines(z,bounds$low, lty=2, col=8)
lines(z,bounds$high, lty=2, col=8)

##Do the same for the measurements at x=4
F<-ecdf(yx4)
plot(F)
yx4s.rrpv<-vector(length=n)
F<-ecdf(yx4)
for(i in 1:n){
yx4s.rrpv[i]<- runif(1,F(yx4s[i]-1), F(yx4s[i]))
}
hist(yx4s.rrpv, 10)
plot(ecdf(c(yx4s.rrpv)), xlim=c(0,1), ylim=c(0,1), col.01line=0, do.points=FALSE, verticals=TRUE)
lines(c(0,1),c(0,1), col=8)
lines(z,bounds$low, lty=2, col=8)
lines(z,bounds$high, lty=2, col=8)


##RPVs or RRPVs can be combined into a single ECDF for all measurements at each level of x in the study, even if the shapes of the theoretical distributions of y are different depending on the level of x. For instance:
plot(ecdf(c(yx025s.rrpv,yx1s.rrpv,yx4s.rrpv)), xlim=c(0,1), ylim=c(0,1), col.01line=0, do.points=FALSE, verticals=TRUE)
lines(c(0,1),c(0,1), col=8)
bounds<- as.data.frame(t(apply(as.matrix(1: (3*n-1)), MARGIN = 1, loop.ptest, N=3*n)))
z<-(1: (3*n-1))/(3*n)
lines(z,bounds$low, lty=2, col=8)
lines(z,bounds$high, lty=2, col=8)

##So a single plot can be used to display the combined goodness of fit of multiple distributions to their respective theoretical distribution, three in this case.
##This can also be done if measurements are taken over a range of x levels, where only one measurement is taken per x level. The shape of the theoretical distribution of each measurement can be unique for each unique x value, and it would not be possible to compare Q-Q plots, since only one measured observation (y-value) is available per unique x value. However, for each unique level of x, the theoretical cumulative distribution function can be calculated, and thus also the RRPV. Then, all these RRPVs can be combined into a single C-R plot (via their ECDF) to assess the overall fit to the presumed underlying distribution. This is illustrated in another example further down. First, what happens if the lab statistician transforms the data and presumes a Gaussian underlying distribution of the logarithmically transformed data?

lm.result<-lm(log(ys+0.25)~xs)
summary(lm.result)
intercept.hat<-lm.result$coef[1]
beta.hat<-lm.result$coef[2]
sigma.hat<-summary(lm.result)$sigma

##Calculate the theoretical distribution by sampling from the Gaussian distribution: 
N<-100000
#The distributions are
nyx025<-rnorm(N, exp(beta.hat*0.25)+intercept.hat, sigma.hat) 
nyx1<-rnorm(N, exp(beta.hat*1)+intercept.hat, sigma.hat) 
nyx4<-rnorm(N, exp(beta.hat*4)+intercept.hat, sigma.hat) 

hist(nyx025, 100)
hist(nyx1, 100) 
hist(nyx4, 100) 

##The Gaussian theoretical distributions all have the same general shapes (but different mean), and their shapes do not match the shapes of the observations ones as well as the Poisson theoretical distributions.

##One could use qqplot again to establish whether the distributions match:
qqplot(x=nyx025, y=yx025s, xlim=c(-5,5), ylim=c(-1,5))
lines(c(0:100), c(0:100), col=8, lty=3) #The diagonal

#or alternatively
qqnorm(y=yx025s, xlim=c(-5,5), ylim=c(-1,5))
qqline(y=yx025s, xlim=c(-5,5), ylim=c(-1,5))

qqplot(nyx1, yx1s)
lines(c(0:100), c(0:100), col=8, lty=3) #The diagonal

qqplot(nyx4, yx4s)
lines(c(0:100), c(0:100), col=8, lty=3) #The diagonal

##With the C-R plot: 
F<-ecdf(nyx025)
plot(F)
yx025s.rpv<-vector(length=n)
for(i in 1:n){
yx025s.rpv[i]<- F(yx025s[i])
}
hist(yx025s.rpv, 20)

plot(ecdf(c(yx025s.rpv)), xlim=c(0,1), ylim=c(0,1), col.01line=0, do.points=FALSE, verticals=TRUE)
lines(c(0,1),c(0,1), col=8)
bounds<- as.data.frame(t(apply(as.matrix(1: (n-1)), MARGIN = 1, loop.ptest, N=n)))
z<-(1: (n-1))/(n)
lines(z,bounds$low, lty=2, col=8)
lines(z,bounds$high, lty=2, col=8)

F<-ecdf(nyx1)
plot(F)
yx1s.rpv<-vector(length=n)
for(i in 1:n){
yx1s.rpv[i]<- F(yx1s[i])
}
hist(yx1s.rpv, 20)
plot(ecdf(c(yx1s.rpv)), xlim=c(0,1), ylim=c(0,1), col.01line=0, do.points=FALSE, verticals=TRUE)
lines(c(0,1),c(0,1), col=8)
lines(z,bounds$low, lty=2, col=8)
lines(z,bounds$high, lty=2, col=8)

F<-ecdf(nyx4)
plot(F)
yx4s.rpv<-vector(length=n)
for(i in 1:n){
yx4s.rpv[i]<- F(yx4s[i])
}
hist(yx4s.rpv, 20)

plot(ecdf(c(yx4s.rpv)), xlim=c(0,1), ylim=c(0,1), col.01line=0, do.points=FALSE, verticals=TRUE)
lines(c(0,1),c(0,1), col=8)
lines(z,bounds$low, lty=2, col=8)
lines(z,bounds$high, lty=2, col=8)

##All combined:
plot(ecdf(c(yx025s.rpv,yx1s.rpv,yx4s.rpv)), xlim=c(0,1), ylim=c(0,1), col.01line=0, do.points=FALSE, verticals=TRUE)
lines(c(0,1),c(0,1), col=8)
bounds<- as.data.frame(t(apply(as.matrix(1: (3*n-1)), MARGIN = 1, loop.ptest, N=3*n)))
z<-(1: (3*n-1))/(3*n)
lines(z,bounds$low, lty=2, col=8)
lines(z,bounds$high, lty=2, col=8)

##The C-R plot shows that the data do not follow the diagonal, and that the model is thus misspecified.


##The following is an example for measurements at unique levels of x, where it is not possible to generate meaningful Q-Q plots.
#Data generation for unique x:
n<-500
beta<-2
xs<-rnorm(n,1,0.2)
ys<-vector(length=n)
for(i in 1:n){
ys[i]<-rpois(1, lambda=exp(beta*xs[i]))
}
glm.result<-glm(ys~xs-1, family="poisson")
summary(glm.result)
beta.hat<-glm.result$coef
beta.hat

##Generate theoretical distributions for each unique measurement
N<-10000
ymat<-matrix(nrow=n, ncol=N)
for(i in 1:n){
ymat[i,]<-rpois(N, lambda=exp(beta.hat*xs[i]))
}
rrpv<-vector(length=n)
for(i in 1:n){
	y.d<-ymat[i,]
	F<-ecdf(y.d)
	rrpv[i]<- runif(1,F(ys[i]-1), F(ys[i]))
}
plot(ecdf(rrpv), xlim=c(0,1), ylim=c(0,1), col.01line=0, do.points=FALSE, verticals=TRUE)
lines(c(0,1),c(0,1), col=8)
bounds<- as.data.frame(t(apply(as.matrix(1: (n-1)), MARGIN = 1, loop.ptest, N=n)))
z<-(1: (n-1))/(n)
lines(z,bounds$low, lty=2, col=8)
lines(z,bounds$high, lty=2, col=8)

##Now compared to an example of misspecification of the underlying distribution (assuming it to be Gaussian), here with log transformation:
ys.prime<- log(ys+0.25)
lm.result<-lm(ys.prime~xs)
summary(lm.result)
intercept.hat<-lm.result$coef[1]
beta.hat<-lm.result$coef[2]
sigma.hat<-summary(lm.result)$sigma
ymat<-matrix(nrow=n, ncol=N)
for(i in 1:n){
ymat[i,]<- exp(rnorm(N, beta.hat*xs[i]+intercept.hat, sigma.hat))-0.25
}

rpv<-vector(length=n)
for(i in 1:n){
	y.d<-ymat[i,]
	F<-ecdf(y.d)
	rpv[i]<- F(ys[i])
}
plot(ecdf(rpv), xlim=c(0,1), ylim=c(0,1), col.01line=0, do.points=FALSE, verticals=TRUE, col=2, add=TRUE)

plot(xs, log(ys+0.25))
lines(xs, (lm.result$fitted.values))
