# Supplementary material for: Generalized Seasonal Autoregressive Integrated Moving Average Models for Count Data with Application to Malaria Time Series with Low Case Numbers
Source: PLoS One. 2013 Jun 13;8(6):e65761. doi: 10.1371/journal.pone.0065761 (PMC3681978; doi:10.1371/journal.pone.0065761)
Supplement: Additional File S4 — R code for an example of simulating and estimating a time series with a Poisson GARIMA(1,1,0) structure, and the use of C-R plots to estimate the appropriateness of the posterior predictive distributions, comparing Poisson and Gaussian models fitted to Poisson data. (RTF) [file pone.0065761.s008.rtf]

Additional file S4
##Additional File S4, supporting information to Briët et al.: “Generalized seasonal autoregressive integrated moving average models for count data with application to malaria time series with low case numbers”
##This file contains computer code for use in the free software R [http://cran.r-project.org/]. It gives an example of simulating and estimating a time series with a Poisson GARIMA(1,1,0) structure. This example uses plots of cumulative distribution function of residual probability values, here called "C-R plot" to estimate the appropriateness of the posterior predictive distributions, and compares these for Poisson and Gaussian models fitted to Poisson data. For the code to run, it requires a few R packages, and JAGS [http://mcmc-jags.sourceforge.net/] to be installed. 

##Simulation
#install.packages("gsarima")
library(gsarima)
N<-1000
phi<-c(0.5)
ar<-arrep(phi=phi, d=1, frequency=12)
intercept<-2
frequency<-1
X=matrix(c(rep(log(intercept), N+length(ar))), ncol=1)
set.seed(123)
y.sim <- garsim(n=(N+length(ar)), phi=ar, beta=c(1), link= "log", family= "poisson", zero.correction = "zq1", c=1, X=X) 
y<-y.sim[(1+length(ar)):(N+length(ar))]
tsy<-ts(y, freq=frequency)
plot(tsy)
#cat(y, sep=" ,") #This simulated data is printed below:

y<-c(5 ,10 ,20 ,28 ,33 ,46 ,57 ,53 ,38 ,39 ,41 ,44 ,46 ,43 ,49 ,58 ,62 ,57 ,46 ,39 ,31 ,33 ,33 ,26 ,17 ,12 ,5 ,5 ,5 ,7 ,11 ,16 ,22 ,28 ,31 ,30 ,22 ,17 ,10 ,11 ,15 ,12 ,12 ,8 ,6 ,5 ,5 ,4 ,3 ,6 ,8 ,13 ,22 ,27 ,38 ,34 ,26 ,23 ,23 ,20 ,27 ,23 ,22 ,23 ,23 ,27 ,40 ,45 ,41 ,34 ,34 ,33 ,28 ,26 ,24 ,23 ,24 ,22 ,17 ,16 ,19 ,22 ,22 ,27 ,35 ,43 ,49 ,47 ,54 ,54 ,65 ,69 ,72 ,88 ,97 ,113 ,115 ,115 ,106 ,107 ,105 ,98 ,99 ,96 ,99 ,103 ,106 ,100 ,106 ,118 ,129 ,138 ,149 ,178 ,202 ,203 ,204 ,190 ,198 ,218 ,253 ,275 ,280 ,301 ,286 ,289 ,289 ,295 ,279 ,245 ,218 ,198 ,204 ,220 ,209 ,214 ,227 ,238 ,227 ,221 ,216 ,190 ,191 ,186 ,176 ,154 ,122 ,111 ,112 ,107 ,108 ,129 ,126 ,124 ,146 ,149 ,173 ,173 ,178 ,174 ,160 ,149 ,118 ,102 ,93 ,105 ,103 ,104 ,101 ,113 ,113 ,134 ,153 ,147 ,128 ,143 ,167 ,176 ,162 ,149 ,142 ,128 ,120 ,117 ,118 ,131 ,131 ,143 ,151 ,188 ,213 ,226 ,217 ,221 ,239 ,259 ,263 ,258 ,244 ,247 ,230 ,220 ,218 ,206 ,212 ,228 ,247 ,254 ,245 ,254 ,266 ,252 ,242 ,214 ,181 ,145 ,125 ,100 ,90 ,100 ,104 ,117 ,131 ,137 ,121 ,110 ,105 ,115 ,145 ,182 ,202 ,187 ,192 ,186 ,196 ,210 ,196 ,185 ,182 ,181 ,186 ,188 ,166 ,145 ,132 ,139 ,158 ,165 ,165 ,164 ,174 ,174 ,166 ,164 ,143 ,117 ,101 ,86 ,75 ,61 ,52 ,34 ,31 ,33 ,24 ,23 ,14 ,14 ,12 ,14 ,17 ,15 ,17 ,19 ,25 ,21 ,13 ,12 ,11 ,12 ,17 ,20 ,26 ,23 ,14 ,12 ,14 ,15 ,12 ,12 ,15 ,23 ,28 ,30 ,21 ,13 ,7 ,4 ,5 ,4 ,0 ,1 ,0 ,1 ,1 ,1 ,0 ,1 ,1 ,1 ,1 ,1 ,1 ,1 ,0 ,0 ,4 ,5 ,5 ,6 ,8 ,13 ,17 ,23 ,30 ,33 ,36 ,31 ,41 ,66 ,91 ,112 ,117 ,103 ,96 ,75 ,60 ,54 ,43 ,40 ,44 ,52 ,51 ,52 ,47 ,47 ,46 ,31 ,33 ,29 ,25 ,21 ,17 ,12 ,9 ,9 ,4 ,3 ,2 ,3 ,5 ,4 ,2 ,0 ,1 ,1 ,1 ,0 ,1 ,4 ,10 ,27 ,26 ,28 ,30 ,33 ,26 ,24 ,23 ,28 ,26 ,28 ,32 ,33 ,26 ,22 ,21 ,21 ,16 ,14 ,9 ,10 ,12 ,14 ,16 ,22 ,20 ,18 ,14 ,14 ,14 ,16 ,15 ,17 ,18 ,19 ,19 ,24 ,37 ,43 ,34 ,33 ,30 ,32 ,28 ,35 ,39 ,48 ,67 ,76 ,72 ,68 ,67 ,80 ,84 ,71 ,65 ,64 ,74 ,80 ,89 ,101 ,117 ,119 ,93 ,81 ,87 ,102 ,98 ,94 ,84 ,80 ,81 ,86 ,82 ,89 ,92 ,88 ,85 ,75 ,76 ,79 ,75 ,74 ,81 ,86 ,82 ,81 ,70 ,56 ,49 ,49 ,49 ,65 ,85 ,98 ,109 ,108 ,97 ,78 ,60 ,58 ,65 ,71 ,68 ,63 ,56 ,48 ,50 ,57 ,51 ,53 ,56 ,51 ,43 ,39 ,46 ,52 ,62 ,65 ,64 ,64 ,57 ,45 ,41 ,40 ,45 ,48 ,54 ,53 ,59 ,50 ,44 ,43 ,35 ,35 ,35 ,26 ,18 ,14 ,8 ,5 ,2 ,4 ,5 ,5 ,6 ,10 ,10 ,9 ,9 ,2 ,0 ,1 ,2 ,4 ,3 ,2 ,3 ,5 ,7 ,5 ,4 ,3 ,4 ,1 ,0 ,2 ,1 ,3 ,4 ,7 ,13 ,17 ,19 ,13 ,11 ,19 ,25 ,33 ,43 ,60 ,71 ,87 ,79 ,84 ,62 ,54 ,38 ,40 ,31 ,26 ,20 ,13 ,10 ,1 ,0 ,2 ,1 ,0 ,4 ,8 ,9 ,6 ,6 ,3 ,2 ,4 ,3 ,6 ,3 ,3 ,3 ,3 ,5 ,7 ,10 ,13 ,13 ,16 ,20 ,19 ,16 ,14 ,16 ,14 ,16 ,9 ,9 ,11 ,12 ,8 ,4 ,2 ,3 ,2 ,2 ,0 ,1 ,0 ,1 ,0 ,2 ,2 ,4 ,5 ,11 ,15 ,17 ,19 ,17 ,15 ,16 ,20 ,19 ,15 ,11 ,10 ,4 ,0 ,0 ,0 ,0 ,1 ,0 ,2 ,2 ,2 ,2 ,1 ,1 ,1 ,0 ,0 ,0 ,1 ,0 ,0 ,0 ,0 ,1 ,1 ,1 ,2 ,2 ,5 ,3 ,3 ,3 ,2 ,3 ,1 ,1 ,0 ,0 ,2 ,1 ,0 ,4 ,9 ,12 ,13 ,15 ,9 ,5 ,1 ,0 ,0 ,2 ,3 ,7 ,6 ,9 ,11 ,9 ,7 ,8 ,7 ,9 ,10 ,15 ,12 ,15 ,21 ,29 ,32 ,35 ,39 ,34 ,31 ,33 ,33 ,29 ,30 ,31 ,27 ,19 ,19 ,10 ,4 ,2 ,1 ,1 ,0 ,1 ,1 ,1 ,1 ,1 ,6 ,18 ,35 ,55 ,88 ,118 ,139 ,123 ,123 ,132 ,126 ,140 ,146 ,155 ,162 ,163 ,185 ,186 ,158 ,135 ,131 ,136 ,118 ,101 ,105 ,104 ,96 ,76 ,68 ,59 ,57 ,70 ,77 ,78 ,73 ,77 ,87 ,92 ,83 ,88 ,72 ,86 ,80 ,84 ,89 ,89 ,100 ,108 ,97 ,98 ,102 ,97 ,78 ,72 ,70 ,68 ,84 ,96 ,101 ,116 ,135 ,134 ,142 ,143 ,158 ,157 ,141 ,151 ,175 ,204 ,201 ,194 ,191 ,206 ,225 ,207 ,209 ,231 ,239 ,245 ,258 ,271 ,283 ,279 ,321 ,335 ,346 ,383 ,375 ,391 ,431 ,451 ,473 ,482 ,499 ,513 ,526 ,497 ,475 ,465 ,433 ,420 ,385 ,376 ,355 ,310 ,294 ,301 ,322 ,367 ,439 ,512 ,598 ,666 ,751 ,832 ,871 ,828 ,802 ,778 ,778 ,749 ,732 ,734 ,708 ,702 ,660 ,651 ,628 ,641 ,636 ,639 ,614 ,612 ,570 ,575 ,562 ,567 ,567 ,573 ,637 ,640 ,671 ,718 ,762 ,785 ,773 ,778 ,825 ,837 ,865 ,815 ,809 ,843 ,857 ,869 ,862 ,864 ,825 ,784 ,763 ,736 ,713 ,696 ,677 ,658 ,668 ,665 ,666 ,649 ,644 ,665 ,678 ,668 ,667 ,672 ,683 ,646 ,636 ,596 ,599 ,619 ,686 ,726 ,748 ,761 ,769 ,721 ,671 ,632 ,599 ,546 ,530 ,507 ,478 ,462 ,470 ,451 ,418 ,395 ,413 ,406 ,404 ,410 ,403 ,413 ,396 ,366 ,357 ,350 ,327 ,306 ,293 ,269 ,262 ,232 ,233 ,237 ,276 ,309 ,318 ,319 ,297 ,289 ,295 ,301 ,290 ,289 ,318 ,330 ,349 ,383 ,384 ,388 ,422 ,469 ,503 ,537 ,526 ,542 ,495 ,493 ,466 ,447 ,438 ,471 ,465 ,435 ,415 ,395 ,359 ,354 ,347 ,349 ,346 ,355 ,343 ,347 ,333 ,312 ,311 ,336 ,337 ,301 ,281 ,266 ,266 ,264 ,291 ,314 ,319 ,298 ,293 ,282 ,260 ,247 ,236 ,235 ,238 ,213 ,199)

#install.packages("R2jags")
library(R2jags)

##Estimation with an appropriate Poisson log link ZQ1 model
model110poisZQ1<-function(){
	##likelihood
	for (t in 3:N){
		y[t]~dpois(lambda[t])
		y.f[t]~dpois(lambda[t])
		lambda[t]<-exp(mu[t])
		mu[t] <- log(max(c,y[t-1]))+phi1*log(max(c,y[t-1])) -phi1*log(max(c,y[t-2]))
	}
	##priors
	r.phi1~dbeta(1,1)
	phi1<-2*r.phi1-1
}
write.model(model110poisZQ1, con = "model110poisZQ1.txt")

data<-list(N=N, y= y[1:N], c=1)
inits<- (list(list(r.phi1=0.1), list(r.phi1=0.8), list(r.phi1=0.5)))
parameters<-c("phi1",  paste("y.f[3:",N,"]",sep=""))

set.seed(123)
ptm <- proc.time()
jags.output.110poisZQ1 <- jags(data= data, inits, parameters, model.file= "model110poisZQ1.txt",
    n.iter=2000, n.burnin=1000, n.chains=3, n.thin=1)
proc.time() - ptm
alarm()

save(jags.output.110poisZQ1 , file= "jagsoutput110poisZQ1.Rdata")
#load("jagsoutput110poisZQ1.Rdata")

print(jags.output.110poisZQ1, digits=2)

#Inference for Bugs model at "model110poisZQ1.txt", fit using jags,
# 3 chains, each with 2000 iterations (first 1000 discarded)
# n.sims = 3000 iterations saved
#          mu.vect sd.vect    2.5%     25%     50%     75%   97.5% Rhat n.eff
#phi1         0.50    0.03    0.45    0.49    0.50    0.52    0.56 1.00  3000
#DIC info (using the rule, pD = var(deviance)/2)
#pD = 1.1 and DIC = 6662.4
#DIC is an estimate of expected predictive error (lower deviance is better).

res.mcmc<-as.mcmc(jags.output.110poisZQ1)
res.mcmc.sel<-res.mcmc[][,1:2]
res.list<-mcmc.list(res.mcmc.sel[[1]],res.mcmc.sel[[2]],res.mcmc.sel[[2]])
gelman.diag(res.list)
gelman.plot(res.list)
w<-2
re<-(jags.output.110poisZQ1$BUGSoutput$median$y.f[1:(N-w)]-y[(w+1):N])/ (y[(w+1):N]+1)
mean(abs(re))
#[1] 0.1587711
##Note that this MARE is the lowest one achievable on this data, with the correct model specification.

##The "loop.rrpv" function calculates the randomized residual probability for discrete data
loop.rrpv<-function(n, y, output){
	y.p1<-output$BUGSoutput$sims.list$y.f[,(n)]
	F<-ecdf(y.p1)
	rrpv<- runif(1,F(y[(n+w)]-1), F(y[(n+w)]))
}

rrpv.jags.output.110poisZQ1 <- apply(as.matrix(1: (N-w)), MARGIN = 1, loop.rrpv, y= y, output= jags.output.110poisZQ1)
plot(ecdf(rrpv.jags.output.110poisZQ1), verticals=TRUE, do.p = FALSE, xlab = "Randomized residual probability", ylab = "Cumulative distribution of randomized cumulative probabilities", main = NULL, col.01line = NULL)


loop.ptest<-function(n, N){
	test<-binom.test(n, N, n/N)
	low <-test$conf.int[1]
	high <-test$conf.int[2]
	res<-c(low, high)
	names(res)<-c("low", "high")
	res
}
bounds<- as.data.frame(t(apply(as.matrix(1: (N-1)), MARGIN = 1, loop.ptest, N=N)))
z<-(1: (N-1))/(N)
lines(c(0,1),c(0,1), col=8)
lines(z,bounds$low, lty=2, col=8)
lines(z,bounds$high, lty=2, col=8)


##This C-R plot shows that the cumulative distribution function follows the diagonal closely, and is within the 95% confidence bounds.


##Estimation with an (inappropriate) Gaussian identity link model on untransformed data

model110gausidentity<-function(){
	##likelihood
	for (t in 3:N){
		y[t]~dnorm(mu[t], tau)
		y.f[t]~dnorm(mu[t], tau)
		mu[t] <- y[t-1]+phi1*y[t-1] -phi1*y[t-2]
	}
	##priors
	r.phi1~dbeta(1,1)
	phi1<-2*r.phi1-1
	tau~dgamma(0.01, 0.01)
}
write.model(model110gausidentity, con = "model110gausidentity.txt")
data<-list(N=N, y= y[1:N])
inits<- (list(list(r.phi1=0.1, tau=1), list(r.phi1=0.8, tau=2), list(r.phi1=0.5, tau=3)))
parameters<-c("phi1",  paste("y.f[3:",N,"]",sep=""), "tau")

set.seed(123)
ptm <- proc.time()
jags.output.110gausidentity <- jags(data= data, inits, parameters, model.file= "model110gausidentity.txt",
    n.iter=2000, n.burnin=1000, n.chains=3, n.thin=1)
proc.time() - ptm
alarm()

save(jags.output.110gausidentity , file= "jagsoutput110gausidentity.Rdata")
#load("jagsoutput110gausidentity.Rdata")

print(jags.output.110gausidentity, digits=2)

#Inference for Bugs model at "model110gausidentity.txt", fit using jags,
# 3 chains, each with 2000 iterations (first 1000 discarded)
# n.sims = 3000 iterations saved
#          mu.vect sd.vect    2.5%     25%     50%     75%   97.5% Rhat n.eff
#phi1         0.56    0.03    0.51    0.54    0.56    0.58    0.61 1.00  3000
#tau          0.01    0.00    0.01    0.01    0.01    0.01    0.01 1.00  3000
#DIC info (using the rule, pD = var(deviance)/2)
#pD = 2.0 and DIC = 7816.8
#DIC is an estimate of expected predictive error (lower deviance is better).

##Note that the the estimate for phi1 is reasonably accurate

res.mcmc<-as.mcmc(jags.output.110gausidentity)
res.mcmc.sel<-res.mcmc[][,c(1:2,4)]
res.list<-mcmc.list(res.mcmc.sel[[1]],res.mcmc.sel[[2]],res.mcmc.sel[[2]])
gelman.diag(res.list)
gelman.plot(res.list)
w<-2
re<-(jags.output.110gausidentity$BUGSoutput$median$y.f[1:(N-w)]-y[(w+1):N])/ (y[(w+1):N]+1)
mean(abs(re))
#[1] 0.1812186

##Note that the mean obsolute relative error (MARE) is higher (worse) than that for the appropriate Poisson model (MARE= 0.159)

##The "loop.rpv" function calculates the residual probability for continuous data
loop.rpv<-function(n, y, output){
	y.p1<-output$BUGSoutput$sims.list$y.f[,(n)]
	F<-ecdf(y.p1)
	rpv<- F(y[(n+w)])
}

w<-2

rrpv.jags.output.110gausidentity <- apply(as.matrix(1: (N-w)), MARGIN = 1, loop.rpv, y= y, output= jags.output.110gausidentity)

plot(ecdf(rrpv.jags.output.110gausidentity), verticals=TRUE, do.p = FALSE, xlab = "Residual probability", ylab = "Cumulative distribution of cumulative probabilities", main = NULL, col.01line = NULL)
lines(c(0,1),c(0,1), col=8)

lines(z,bounds$low, lty=2, col=8)
lines(z,bounds$high, lty=2, col=8)


##This plot shows that the cumulative distribution function does not follow the diagonal closely, and is mostly outside the 95% confidence bounds. For values of the residual probability below 0.5, there are too few observations, and for values above 0.5, there are too many. This indicates that predictive distributions are well centred but platykurtic. 

##Estimation with an (inappropriate) Gaussian identity link model on transformed data

qqnorm(y, main=NULL)
qqline(y) 
##A visual check shows that the data are not normally distributed. A common transformation is log(y+1):
y.bc<-log(y+1)
qqnorm(y.bc, main=NULL)
qqline(y.bc) 

model110gausidentitybcL<-function(){
	#likelihood
	for (t in 3:N){
		y.bc[t]~dnorm(mu[t], tau)
		y.bc.f[t]~dnorm(mu[t], tau)
		mu[t] <- y.bc[t-1]+phi1*y.bc[t-1] -phi1*y.bc[t-2]
	y.f[t]<- exp(y.bc.f[t])-1 
	}
	#priors
	r.phi1~dbeta(1,1)
	phi1<-2*r.phi1-1
	tau~dgamma(0.01, 0.01)
}
write.model(model110gausidentitybcL, con = "model110gausidentitybcL.txt")
data<-list(N=N, y.bc= y.bc3[1:N])
inits<- (list(list(r.phi1=0.1, tau=1), list(r.phi1=0.8, tau=2), list(r.phi1=0.5, tau=3)))
parameters<-c("phi1", paste("y.f[3:",N,"]",sep=""), "tau")

set.seed(123)
ptm <- proc.time()
jags.output.110gausidentitybcL <- jags(data= data, inits, parameters, model.file= "model110gausidentitybcL.txt",
    n.iter=2000, n.burnin=1000, n.chains=3, n.thin=1)
proc.time() - ptm
alarm()

save(jags.output.110gausidentitybcL , file= "jagsoutput110gausidentitybcL.Rdata")
#load("jagsoutput110gausidentitybcL.Rdata")

print(jags.output.110gausidentitybcL, digits=2)
#Inference for Bugs model at "model110gausidentitybcL.txt", fit using jags,
# 3 chains, each with 2000 iterations (first 1000 discarded)
# n.sims = 3000 iterations saved
#          mu.vect sd.vect   2.5%    25%    50%     75%   97.5% Rhat n.eff
#phi1         0.14    0.03   0.08   0.12   0.14    0.16    0.20 1.00  2200
#tau         12.38    0.56  11.31  11.99  12.38   12.76   13.48 1.00  3000
#DIC info (using the rule, pD = var(deviance)/2)
#pD = 2.1 and DIC = 324.3
#DIC is an estimate of expected predictive error (lower deviance is better).

##Note that the the estimate for phi1 is not accurate

res.mcmc<-as.mcmc(jags.output.110gausidentitybcL)
res.mcmc.sel<-res.mcmc[][,c(1:3)]
res.list<-mcmc.list(res.mcmc.sel[[1]],res.mcmc.sel[[2]],res.mcmc.sel[[2]])
gelman.diag(res.list)
gelman.plot(res.list)
w<-2
re<-(jags.output.110gausidentitybcL$BUGSoutput$median$y.f[1:(N-w)]-y[(w+1):N])/ (y[(w+1):N]+1)
mean(abs(re))
#[1] 0.1726938
## Note that this MARE is intermediate between the Gaussian analysis on untransformed data, and the correct Poisson analysis, despite the inaccurate phi1 estimate.

set.seed(123)
rrpv.jags.output.110gausidentitybcL <- apply(as.matrix(1: (N-w)), MARGIN = 1, loop.rpv, y= y, output= jags.output.110gausidentitybcL)

plot(ecdf(rrpv.jags.output.110gausidentitybcL), verticals=TRUE, do.p = FALSE, xlab = "Residual probability", ylab = "Cumulative distribution of cumulative probabilities", main = NULL, col.01line = NULL)
lines(c(0,1),c(0,1), col=8)
lines(z,bounds$low, lty=2, col=8)
lines(z,bounds$high, lty=2, col=8)


##This C-R plot shows that the cumulative distribution function does not follow the diagonal closely, and is mostly outside the 95% confidence bounds. For values of the residual probability below 0.5, there are too few observations, and for values above 0.5, there are too many. This indicates that predictive distributions are well centred but platykurtic. In this example, the C-R plot for the transformed series is even worse than for the not transformed series. Thus, despite the better median predictions (as given by the MARE), the shape of the prediction distributions is inappropriate. 

##Estimation with an (inappropriate) Poisson identity link model (on untransformed data)

model110poisidentity<-function(){
	##likelihood
	for (t in 3:N){
		y[t]~dpois(lambda[t]) 
		y.f[t]~dpois(lambda[t])
		lambda[t]<-max(0.000000001,mu[t])
	#	lambda[t]<-ifelse (mu[t]<=0, 0.0000001, mu[t])
		mu[t] <-  y[t-1]+phi1*y[t-1] -phi1*y[t-2]
	}
	##priors
	r.phi1~dbeta(1,1)
	phi1<-2*r.phi1-1
}
write.model(model110poisidentity, con = "model110poisidentity.txt")
data<-list(N=N, y= y[1:N])
inits<- (list(list(r.phi1=0.1), list(r.phi1=0.2), list(r.phi1=0.3)))
parameters<-c("phi1",  paste("y.f[3:",N,"]",sep=""))

set.seed(123)
ptm <- proc.time()
jags.output.110poisidentity <- jags(data= data, inits, parameters, model.file= "model110poisidentity.txt",
    n.iter=2000, n.burnin=1000, n.chains=3, n.thin=1)
proc.time() - ptm
alarm()

save(jags.output.110poisidentity  , file= "jagsoutput110poisidentity.Rdata")
#load("jagsoutput110poisidentity.Rdata")

print(jags.output.110poisidentity, digits=2)
#Inference for Bugs model at "model110poisidentity.txt", fit using jags,
# 3 chains, each with 2000 iterations (first 1000 discarded)
# n.sims = 3000 iterations saved
#          mu.vect sd.vect    2.5%     25%     50%     75%   97.5% Rhat n.eff
#phi1        -0.04    0.01   -0.05   -0.04   -0.03   -0.03   -0.02 1.00  1500
#deviance  7623.70    1.44 7622.65 7622.76 7623.18 7624.06 7627.81 1.00  3000
#
#For each parameter, n.eff is a crude measure of effective sample size,
#and Rhat is the potential scale reduction factor (at convergence, Rhat=1).
#
#DIC info (using the rule, pD = var(deviance)/2)
#pD = 1.0 and DIC = 7624.7
#DIC is an estimate of expected predictive error (lower deviance is better).

##Note that the the estimate for phi1 is not accurate 

res.mcmc<-as.mcmc(jags.output.110poisidentity)
res.mcmc.sel<-res.mcmc[][,1:2]
res.list<-mcmc.list(res.mcmc.sel[[1]],res.mcmc.sel[[2]],res.mcmc.sel[[2]])
gelman.diag(res.list)
gelman.plot(res.list)
w<-2
re<-(jags.output.110poisidentity$BUGSoutput$median$y.f[1:(N-w)]-y[(w+1):N])/ (y[(w+1):N]+1)
mean(abs(re))
# [1] 0.1780247
##Note that this MARE is higher (worse) than that of the Gaussian analysis on transformed data.

##The "loop.rrpv" function calculates the randomized residual probability value for discrete data
loop.rrpv<-function(n, y, output){
	y.p1<-output$BUGSoutput$sims.list$y.f[,(n)]
	F<-ecdf(y.p1)
	rrpv<- runif(1,F(y[(n+w)]-1), F(y[(n+w)]))
}

rrpv.jags.output.110poisidentity <- apply(as.matrix(1: (N-w)), MARGIN = 1, loop.rrpv, y= y, output= jags.output.110poisidentity)
plot(ecdf(rrpv.jags.output.110poisidentity), verticals=TRUE, do.p = FALSE, xlab = "Randomized residual probability", ylab = "Cumulative distribution of randomized cumulative probabilities", main = NULL, col.01line = NULL)
lines(c(0,1),c(0,1), col=8)
lines(z,bounds$low, lty=2, col=8)
lines(z,bounds$high, lty=2, col=8)


##This C-R plot shows that the cumulative distribution function is mostly outside the 95% confidence bounds, yet it looks better than for the Gaussian models. The aberrations are also in the opposite direction compared to the Gaussian models: for values of the residual probability below 0.5, there are too many observations, and for values above 0.5, there are too few. This indicates that predictive distributions are well centred but leptokurtic. Even though the Gaussian model on transformed data gave better median predictions (as given by the MARE), the shape of the prediction distributions of the (still) misspecified Poisson model with identity link are more appropriate. 
