# Supplementary material for: Generalized Seasonal Autoregressive Integrated Moving Average Models for Count Data with Application to Malaria Time Series with Low Case Numbers
Source: PLoS One. 2013 Jun 13;8(6):e65761. doi: 10.1371/journal.pone.0065761 (PMC3681978; doi:10.1371/journal.pone.0065761)
Supplement: Additional File S5 — R code for an example of simulating and estimating a time series with a Poisson GARIMA(1,0,0) structure, and the effects of misspecification. (RTF) [file pone.0065761.s009.rtf]

Additional file S5
##Additional File S5, supporting information to Briët et al.: “Generalized seasonal autoregressive integrated moving average models for count data with application to malaria time series with low case numbers”
##This file contains computer code for use in the free software R [http://cran.r-project.org/]. It gives an example of simulating and estimating a time series with a Poisson GARIMA(1,0,0) structure, and the effects of misspecification. For the code to run, it requires a few R packages, and JAGS [http://mcmc-jags.sourceforge.net/] to be installed. 

##Simulation
#install.packages("gsarima")
library(gsarima)
N<-1000
ar<-c(0.5)
intercept<-2
frequency<-1
X=matrix(c(rep(log(intercept), N+length(ar))), ncol=1)
y.sim <- garsim(n=(N+length(ar)), phi=ar, beta=c(1), link= "log", family= "poisson", zero.correction = "zq1", c=1, X=X) 
y<-y.sim[(1+length(ar)):(N+length(ar))]
tsy<-ts(y, freq=frequency)
plot(tsy)

#y<-c(4, 0, 0, 0, 1, 1, 0, 5, 0, 1, 2, 1, 1, 0, 4, 1, 1, 2, 2, 1, 1, 0, 0, 0, 3, 2, 5, 0, 0, 2, 1, 1, 2, 2, 5, 3, 4, 0, 4, 10, 3, 3, 4, 4, 3, 4, 3, 3, 2, 3, 1, 1, 1, 3, 2, 3, 0, 2, 1, 0, 1, 1, 1, 0, 2, 3, 4, 1, 2, 3, 6, 8, 1, 1, 0, 0, 3, 4, 2, 1, 0, 2, 2, 3, 0, 0, 1, 1, 3, 2, 0, 0, 3, 7, 8, 6, 5, 2, 3, 0, 0, 1, 1, 5, 5, 4, 4, 1, 1, 1, 2, 6, 5, 1, 3, 1, 3, 3, 1, 0, 1, 1, 1, 0, 2, 3, 2, 0, 1, 0, 3, 1, 1, 3, 3, 1, 0, 0, 2, 6, 4, 4, 2, 2, 2, 0, 3, 3, 5, 2, 1, 0, 3, 3, 2, 1, 1, 1, 4, 1, 0, 3, 3, 6, 4, 5, 2, 4, 3, 2, 4, 7, 3, 3, 0, 2, 2, 2, 3, 2, 4, 4, 5, 2, 4, 1, 0, 1, 0, 1, 1, 1, 1, 1, 1, 2, 1, 0, 3, 0, 1, 1, 1, 1, 0, 2, 2, 1, 0, 1, 1, 1, 2, 2, 2, 1, 1, 2, 1, 1, 2, 5, 4, 2, 1, 2, 1, 4, 1, 1, 0, 2, 0, 2, 2, 3, 2, 1, 3, 3, 6, 3, 5, 2, 2, 2, 2, 3, 5, 6, 8, 2, 4, 8, 7, 3, 2, 2, 1, 1, 1, 0, 0, 1, 3, 4, 0, 1, 1, 1, 0, 1, 1, 0, 2, 1, 1, 0, 1, 1, 0, 3, 1, 1, 1, 1, 1, 3, 2, 4, 6, 2, 3, 3, 2, 3, 2, 1, 1, 0, 0, 0, 3, 4, 0, 3, 3, 0, 2, 0, 0, 3, 1, 1, 2, 1, 0, 2, 4, 1, 2, 1, 1, 1, 0, 2, 5, 4, 3, 1, 1, 0, 2, 2, 0, 2, 4, 1, 1, 0, 0, 3, 4, 0, 4, 2, 2, 2, 2, 1, 0, 3, 2, 2, 4, 3, 2, 5, 2, 0, 1, 0, 3, 1, 1, 6, 2, 0, 2, 1, 1, 0, 1, 2, 2, 2, 5, 3, 4, 3, 1, 1, 0, 2, 1, 0, 0, 1, 1, 2, 2, 0, 5, 2, 2, 0, 2, 0, 0, 1, 2, 3, 1, 2, 3, 2, 0, 1, 1, 2, 5, 2, 5, 1, 1, 1, 2, 0, 3, 3, 2, 3, 2, 2, 0, 0, 1, 0, 1, 2, 0, 3, 3, 2, 3, 4, 2, 2, 0, 1, 0, 2, 1, 1, 2, 1, 0, 2, 2, 2, 3, 3, 2, 0, 0, 2, 4, 5, 5, 4, 5, 3, 2, 3, 4, 9, 5, 4, 2, 1, 1, 4, 4, 5, 0, 2, 2, 1, 0, 4, 2, 3, 2, 0, 0, 0, 2, 0, 2, 5, 4, 3, 4, 0, 2, 3, 4, 5, 5, 2, 4, 1, 1, 1, 2, 2, 4, 2, 1, 2, 4, 3, 2, 1, 2, 3, 1, 0, 3, 2, 3, 6, 3, 2, 2, 0, 3, 3, 1, 2, 3, 5, 4, 2, 2, 3, 3, 4, 2, 2, 3, 2, 1, 2, 1, 2, 3, 4, 2, 1, 0, 1, 2, 2, 1, 2, 1, 0, 1, 3, 2, 3, 1, 0, 1, 2, 3, 0, 1, 1, 1, 3, 2, 3, 1, 2, 1, 1, 1, 4, 1, 1, 0, 1, 0, 2, 2, 0, 4, 2, 1, 2, 2, 0, 2, 1, 1, 0, 4, 3, 0, 0, 2, 4, 3, 4, 2, 1, 1, 0, 1, 2, 2, 2, 1, 3, 5, 6, 5, 3, 1, 1, 0, 2, 3, 2, 2, 0, 0, 0, 0, 1, 5, 3, 3, 2, 0, 0, 1, 1, 1, 1, 1, 1, 0, 2, 4, 4, 1, 2, 2, 1, 1, 1, 0, 1, 0, 0, 0, 2, 3, 3, 3, 1, 3, 1, 0, 0, 0, 4, 1, 0, 2, 2, 2, 2, 2, 4, 2, 1, 0, 3, 3, 5, 3, 0, 0, 1, 2, 1, 1, 0, 3, 0, 1, 2, 0, 2, 3, 3, 2, 0, 5, 3, 2, 3, 4, 2, 3, 2, 1, 4, 2, 0, 3, 4, 2, 0, 1, 0, 0, 0, 1, 2, 1, 2, 4, 1, 1, 2, 2, 2, 1, 4, 2, 2, 2, 1, 2, 3, 1, 2, 2, 3, 2, 4, 5, 5, 1, 2, 2, 2, 1, 3, 1, 2, 0, 2, 1, 1, 1, 3, 1, 1, 1, 0, 0, 0, 1, 1, 2, 1, 0, 0, 1, 2, 4, 0, 1, 0, 0, 1, 2, 1, 2, 1, 0, 2, 3, 2, 1, 3, 2, 0, 0, 1, 0, 1, 4, 1, 1, 4, 5, 2, 0, 1, 2, 4, 3, 0, 0, 2, 0, 2, 1, 2, 2, 2, 2, 1, 2, 2, 1, 1, 0, 2, 0, 1, 4, 2, 2, 3, 3, 0, 3, 3, 1, 4, 1, 1, 1, 1, 1, 1, 1, 2, 1, 1, 3, 6, 3, 1, 4, 4, 5, 6, 1, 1, 3, 4, 2, 0, 0, 2, 2, 0, 1, 3, 2, 1, 1, 1, 3, 1, 0, 0, 4, 3, 3, 1, 1, 1, 1, 2, 2, 1, 0, 0, 2, 3, 1, 1, 2, 1, 1, 2, 3, 4, 2, 2, 3, 0, 0, 2, 1, 1, 0, 2, 3, 1, 1, 0, 0, 0, 1, 1, 0, 2, 2, 3, 2, 0, 0, 2, 2, 1, 4, 2, 0, 2, 7, 2, 2, 3, 5, 5, 4, 3, 2, 2, 2, 1, 3, 5, 5, 3, 2, 1, 0, 1, 2, 3, 3, 1, 1, 2, 4, 5, 3, 6, 4, 6, 6, 10, 2, 3, 1, 2, 2, 3, 1, 1, 0, 2, 2, 3, 3, 2, 0, 1, 2, 4, 3, 2, 0, 1, 2, 1, 1, 1, 3, 1, 1, 2, 2, 0, 0, 2, 3, 3)
 
#install.packages("R2jags")
library(R2jags)
model100poisidentity<-function(){
	##likelihood
	for (t in 2:N){
		y[t]~dpois(lambda[t])
		y.f[t]~dpois(lambda[t])
		lambda[t]<-max(0,mu[t])
		mu[t] <- beta.0 + phi1*y[t-1] - phi1* beta.0
	}
	##priors
	r.phi1~dbeta(1,1)
	phi1<-2*r.phi1-1
	beta.0~dnorm(0, 0.001) #Vague normal prior
	intercept<- beta.0
}
write.model(model100poisidentity, con = "model100poisidentity.txt")
data<-list(N=N, y= y[1:N])
inits<- (list(list(r.phi1=0.1, beta.0=10), list(r.phi1=0.8, beta.0=10), list(r.phi1=0.5, beta.0=20)))
parameters<-c("phi1", "beta.0", "intercept", paste("y.f[2:",N,"]",sep=""))

ptm <- proc.time()
jags.output.100poisidentity <- jags(data= data, inits, parameters, model.file= "model100poisidentity.txt",
    n.iter=2000, n.burnin=1000, n.chains=3, n.thin=1)
proc.time() - ptm
alarm()
#  user  system elapsed 
#  86.64    0.63   87.41 
print(jags.output.100poisidentity, digits=2)
#Inference for Bugs model at "model100poisidentity.txt", fit using jags,
# 3 chains, each with 2000 iterations (first 1000 discarded)
# n.sims = 3000 iterations saved
#          mu.vect sd.vect    2.5%     25%     50%     75%   97.5% Rhat n.eff
#beta.0       1.94    0.07    1.81    1.90    1.94    1.99    2.08 1.00  2400
#intercept    1.94    0.07    1.81    1.90    1.94    1.99    2.08 1.00  2400
#phi1         0.37    0.03    0.32    0.35    0.37    0.39    0.43 1.00  3000
#pD = 1.9 and DIC = 3377.4

res.mcmc<-as.mcmc(jags.output.100poisidentity)
res.mcmc.sel<-res.mcmc[][,c(1:2,4)]
res.list<-mcmc.list(res.mcmc.sel[[1]],res.mcmc.sel[[2]],res.mcmc.sel[[2]])
gelman.diag(res.list)
gelman.plot(res.list)
w<-1
re<-(jags.output.100poisidentity$BUGSoutput$median$y.f[1:(N-w)]-y[(w+1):N])/ (y[(w+1):N]+1)
mean(abs(re))
#[1] 0.4741512

##Estimation log link zq1#
model100poisZQ1<-function(){
	##likelihood
	for (t in 2:N){
		y[t]~dpois(lambda[t])
		y.f[t]~dpois(lambda[t])
		lambda[t]<-exp(mu[t])
		mu[t] <- beta.0 + phi1*log(max(c,y[t-1])) - phi1* beta.0
	}
	##priors
	r.phi1~dbeta(1,1)
	phi1<-2*r.phi1-1
	beta.0~dnorm(0,0.001) #Vague normal prior
	intercept<-exp(beta.0)
}
write.model(model100poisZQ1, con = "model100poisZQ1.txt")

data<-list(N=N, y= y[1:N], c=1)
inits<- (list(list(r.phi1=0.1, beta.0=1), list(r.phi1=0.8, beta.0=1), list(r.phi1=0.5, beta.0=2)))
parameters<-c("phi1", "beta.0", "intercept", paste("y.f[2:",N,"]",sep=""))

ptm <- proc.time()
jags.output.100poisZQ1 <- jags(data= data, inits, parameters, model.file= "model100poisZQ1.txt",
    n.iter=2000, n.burnin=1000, n.chains=3, n.thin=1)
proc.time() - ptm
alarm()
print(jags.output.100poisZQ1, digits=2)

#Inference for Bugs model at "model100poisZQ1.txt", fit using jags,
# 3 chains, each with 2000 iterations (first 1000 discarded)
# n.sims = 3000 iterations saved
#          mu.vect sd.vect    2.5%     25%     50%     75%   97.5% Rhat n.eff
#beta.0       0.66    0.05    0.56    0.63    0.66    0.69    0.75 1.00  3000
#intercept    1.94    0.10    1.74    1.87    1.94    2.00    2.13 1.00  3000
#phi1         0.54    0.04    0.46    0.51    0.54    0.56    0.61 1.00  3000
#pD = 1.9 and DIC = 3354.5

res.mcmc<-as.mcmc(jags.output.100poisZQ1)
res.mcmc.sel<-res.mcmc[][,1:4]
summary(res.mcmc)
plot(res.mcmc)
autocorr.plot(res.mcmc)
res.list<-mcmc.list(res.mcmc.sel[[1]],res.mcmc.sel[[2]],res.mcmc.sel[[2]])
gelman.diag(res.list)
gelman.plot(res.list)
w<-1
re<-(jags.output.100poisZQ1$BUGSoutput$median$y.f[1:(N-w)]-y[(w+1):N])/ (y[(w+1):N]+1)
mean(abs(re))
#[1] 0.4747097

data<-list(N=N, y= y[1:N], c=0.1)
ptm <- proc.time()
jags.output.100poisZQ1c01 <- jags(data= data, inits, parameters, model.file= "model100poisZQ1.txt",
    n.iter=2000, n.burnin=1000, n.chains=3, n.thin=1)
proc.time() - ptm
alarm()
print(jags.output.100poisZQ1c01, digits=2)
#Inference for Bugs model at "model100poisZQ1.txt", fit using jags,
# 3 chains, each with 2000 iterations (first 1000 discarded)
# n.sims = 3000 iterations saved
#          mu.vect sd.vect    2.5%     25%     50%     75%   97.5% Rhat n.eff
#beta.0       0.76    0.03    0.70    0.74    0.76    0.78    0.82 1.00  2100
#intercept    2.13    0.06    2.01    2.09    2.13    2.18    2.26 1.00  2000
#phi1         0.21    0.02    0.17    0.20    0.21    0.23    0.26 1.00  3000
#pD = 2.1 and DIC = 3441.7
res.mcmc<-as.mcmc(jags.output.100poisZQ1c01)
res.mcmc.sel<-res.mcmc[][,1:4]
res.list<-mcmc.list(res.mcmc.sel[[1]],res.mcmc.sel[[2]],res.mcmc.sel[[2]])
gelman.diag(res.list)
gelman.plot(res.list)
w<-1
re<-(jags.output.100poisZQ1c01$BUGSoutput$median$y.f[1:(N-w)]-y[(w+1):N])/ (y[(w+1):N]+1)
mean(abs(re))
#[1] 0.5508359

##ZQ2
library(R2jags)
model100poisZQ2<-function(){
	##likelihood
	for (t in 2:N){
		y[t]~dpois(lambda[t])
		y.f[t]~dpois(lambda[t])
		lambda[t]<-exp(mu[t])
		mu[t] <- beta.0 + phi1*log(y[t-1]+c) - phi1* log(exp(beta.0)+c)
	}
	##priors
	r.phi1~dbeta(1,1)
	phi1<-2*r.phi1-1
	beta.0~dnorm(0,0.001) #Vague normal prior
	intercept<-exp(beta.0)
}
write.model(model100poisZQ2, con = "model100poisZQ2.txt")

##c=1
data<-list(N=N, y= y[1:N], c=1)
inits<- (list(list(r.phi1=0.1, beta.0=1), list(r.phi1=0.8, beta.0=1), list(r.phi1=0.5, beta.0=2)))
parameters<-c("phi1", "beta.0", "intercept", paste("y.f[2:",N,"]",sep=""))

ptm <- proc.time()
jags.output.100poisZQ2 <- jags(data= data, inits, parameters, model.file= "model100poisZQ2.txt",
    n.iter=2000, n.burnin=1000, n.chains=3, n.thin=1)
proc.time() - ptm
alarm()
print(jags.output.100poisZQ2, digits=2)
#Inference for Bugs model at "model100poisZQ2.txt", fit using jags,
# 3 chains, each with 2000 iterations (first 1000 discarded)
# n.sims = 3000 iterations saved
#          mu.vect sd.vect    2.5%     25%     50%     75%   97.5% Rhat n.eff
#beta.0       0.72    0.04    0.64    0.69    0.72    0.74    0.79 1.00  3000
#intercept    2.05    0.07    1.91    2.00    2.05    2.10    2.20 1.00  3000
#phi1         0.54    0.04    0.46    0.51    0.54    0.57    0.62 1.00  3000
#pD = 1.9 and DIC = 3393.1

res.mcmc<-as.mcmc(jags.output.100poisZQ2)
res.mcmc.sel<-res.mcmc[][,1:4]
res.list<-mcmc.list(res.mcmc.sel[[1]],res.mcmc.sel[[2]],res.mcmc.sel[[2]])
gelman.diag(res.list)
gelman.plot(res.list)
w<-1
re<-(jags.output.100poisZQ2$BUGSoutput$median$y.f[1:(N-w)]-y[(w+1):N])/ (y[(w+1):N]+1)
mean(abs(re))
#[1] 0.4666005

##c=0.1
data<-list(N=N, y= y[1:N], c=0.1)
inits<- (list(list(r.phi1=0.1, beta.0=1), list(r.phi1=0.8, beta.0=1), list(r.phi1=0.5, beta.0=2)))
parameters<-c("phi1", "beta.0", "intercept", paste("y.f[2:",N,"]",sep=""))

ptm <- proc.time()
jags.output.100poisZQ2c01 <- jags(data= data, inits, parameters, model.file= "model100poisZQ2.txt",
    n.iter=2000, n.burnin=1000, n.chains=3, n.thin=1)
proc.time() - ptm
alarm()
print(jags.output.100poisZQ2c01, digits=2)
#Inference for Bugs model at "model100poisZQ2.txt", fit using jags,
# 3 chains, each with 2000 iterations (first 1000 discarded)
# n.sims = 3000 iterations saved
#          mu.vect sd.vect    2.5%     25%     50%     75%   97.5% Rhat n.eff
#beta.0       0.76    0.03    0.70    0.74    0.76    0.77    0.82 1.00  3000
#intercept    2.13    0.06    2.01    2.09    2.13    2.17    2.26 1.00  3000
#phi1         0.21    0.02    0.17    0.19    0.21    0.22    0.25 1.00  1600
#pD = 2.3 and DIC = 3447.4

res.mcmc<-as.mcmc(jags.output.100poisZQ2c01)
res.mcmc.sel<-res.mcmc[][,1:4]
res.list<-mcmc.list(res.mcmc.sel[[1]],res.mcmc.sel[[2]],res.mcmc.sel[[2]])
gelman.diag(res.list)
gelman.plot(res.list)
w<-1
re<-(jags.output.100poisZQ2c01$BUGSoutput$median$y.f[1:(N-w)]-y[(w+1):N])/ (y[(w+1):N]+1)
mean(abs(re))
#[1] 0.5508193
###############
