# Supplementary material for: Generalized Seasonal Autoregressive Integrated Moving Average Models for Count Data with Application to Malaria Time Series with Low Case Numbers
Source: PLoS One. 2013 Jun 13;8(6):e65761. doi: 10.1371/journal.pone.0065761 (PMC3681978; doi:10.1371/journal.pone.0065761)
Supplement: Appendix S1 — Appendix. (PDF) [file pone.0065761.s010.pdf]

Appendix S1

Example of $\text{GARMA}(1,1)$ *model with logarithmic link function and ZQ1 transformation*

For a $\text{GARMA}(1,1)$ model, thus with $p = q = 1$, with logarithmic link function and ZQ1 transformation, $\Phi_p(B) = 1 - \phi_1 B^1$ and $\Theta_q(B) = 1 - \theta_1 B^1$.

Equation 2 can then be written:

$$\begin{aligned}\log(\lambda_t) &= (1-\phi_1 B^1)\left[\mathbf{x}_t^T\boldsymbol{\beta} - \log(y'_t)\right] + \log(y'_t) \\ &\quad -(1-\theta_1 B^1)\log(y'_t/\lambda_t) + \log(y'_t/\lambda_t)\end{aligned},$$

which simplifies into:

$$\log(\lambda_t) = \mathbf{x}_t^T\boldsymbol{\beta} - \phi_1 B^1\left[\mathbf{x}_t^T\boldsymbol{\beta} - \log(y'_t)\right] + \theta_1 B^1\left[\log(y'_t/\lambda_t)\right].$$

Assuming a non zero mean and single covariate $x_{1,t}$, (thus $v = 1$), this can be written:

$$\log(\lambda_t) = x_{0,t}\beta_0 + x_{1,t}\beta_1 - \phi_1 x_{0,t-1}\beta_0 - \phi_1 x_{1,t-1}\beta_1 + \phi_1\log(y'_{t-1}) + \theta_1\log(y'_{t-1}/\lambda_{t-1}).$$

Assuming $x_{0,t} = x_0 = 1$, this simplifies into:

$$\log(\lambda_t) = \beta_0 + x_{1,t}\beta_1 - \phi_1\beta_0 - \phi_1 x_{1,t-1}\beta_1 + \phi_1\log(y'_{t-1}) + \theta_1\log(y'_{t-1}/\lambda_{t-1}).$$

Example of $\text{GSARIMA}(0,0,0)\times(1,1,0)_s$ *model with logarithmic link function and ZQ1 transformation*

For a $\text{GSARIMA}(0,0,0)\times(1,1,0)_s$ model, thus with $P = D = 1$, with logarithmic link function and ZQ1 transformation, $\Phi_P^*(B^s) = 1 - \phi_1^* B^s$ and $\Phi_p(B) = \Theta_q(B) = \Theta_q^*(B) = 1$.

Equation 2 can then be written:

$$\log(\lambda_t) = (1-B^s)^1(1-\phi_1^* B^s)\left[\mathbf{x}_t^T\boldsymbol{\beta} - \log(y'_t)\right] + \log(y'_t),$$

which simplifies to:

$$\log(\lambda_t) = \mathbf{x}_t^T\boldsymbol{\beta} - (\phi_1^* + 1)\left[\mathbf{x}_{t-s}^T\boldsymbol{\beta} - \log(y'_{t-s})\right] + \phi_1^*\left[\mathbf{x}_{t-2s}^T\boldsymbol{\beta} - \log(y'_{t-2s})\right].$$

Assuming a non zero mean and single covariate $x_{1,t}$, (thus $v = 1$), this can be written:

$$\log(\lambda_t) = x_{0,t}\beta_0 + x_{1,t}\beta_1 - (\phi_1^* + 1)\left[x_{0,t-s}\beta_0 + x_{1,t-s}\beta_1 - \log(y'_{t-s})\right] + \phi_1^*\left[x_{0,t-2s}\beta_0 + x_{1,t-2s}\beta_1 - \log(y'_{t-2s})\right]$$

Assuming $x_{0,t} = x_0 = 1$, this simplifies into:

$$\log(\lambda_t) = \beta_0 + x_{1,t}\beta_1 - (\phi_1^* + 1)\left[\beta_0 + x_{1,t-s}\beta_1 - \log(y'_{t-s})\right] + \phi_1^*\left[\beta_0 + x_{1,t-2s}\beta_1 - \log(y'_{t-2s})\right]$$

The choice of link function and choices for data transformations in case of a logarithmic link function and observations with the value zero

For Poisson AR(1) models with identity link function and logarithmic link function (with transformation ZQ1 and ZQ2), the distribution properties of simulated series of length 1,000,000 were compared for an intercept $\exp(\beta_0)=2$, $\exp(\beta_0)=10$ or $\exp(\beta_0)=100$, and a coefficient $\phi_1=-0.5$ or $\phi_1=0.5$, and a constant $c=0.1$, or $c=1$ (Table 1 in this Appendix). The software used for simulation of the series has been made available as the R package 'gsarima' [1].

For a (high) intercept of $\exp(\beta_0)=100$, all models had near identical results, although the log-link models resulted in slightly lower mean, variance, skewness and kurtosis with a positive ϕ_1, and vice versa for a negative ϕ_1. This effect became stronger at a lower intercept of $\exp(\beta_0)=10$. The impact of the choice for constant *c* (in the log-link models) was strong at the lower intercepts $\exp(\beta_0)=2$ and $\exp(\beta_0)=10$ (for the latter, for $\phi_1=-0.5$), and a value of $c=1$ gave results most similar to the model with identity link function. The choice for the transformation method gave variable results: at a low intercept $\exp(\beta_0)=2$ (and $c=1$), the mean, variance, skewness and kurtosis were more similar to the identity link model for ZQ1 (except the variance for $\phi_1=-0.5$), whereas at the intercept of $\exp(\beta_0)=10$ (and $c=1$), the mean, variance, skewness and kurtosis were more similar to the identity link model for ZQ2 (except the variance and kurtosis for $\phi_1=0.5$).

Table 1 - Distribution properties of simulated series of different Poisson AR(1) models.

Model				mean	variance	skewness	kurtosis
Link	Exp(β_0)	ϕ_1	c				
identity	2	0.5		2.00	2.67	1.06	4.54
log-ZQ1	2	0.5	0.1	1.54	2.32	1.14	4.41
log-ZQ1	2	0.5	1.0	1.98	2.35	0.91	4.02
log-ZQ2	2	0.5	0.1	1.55	2.30	1.12	4.29
log-ZQ2	2	0.5	1.0	1.91	2.15	0.84	3.80
identity	2	-0.5		2.01	2.62	0.83	3.63
log-ZQ1	2	-0.5	0.1	3.01	10.05	1.73	5.90
log-ZQ1	2	-0.5	1.0	2.14	2.50	0.82	3.73
log-ZQ2	2	-0.5	0.1	3.03	10.47	1.75	5.94
log-ZQ2	2	-0.5	1.0	2.16	2.55	0.87	3.95
identity	10	0.5		10.00	13.37	0.47	3.31
log-ZQ1	10	0.5	0.1	9.61	13.37	0.40	3.18
log-ZQ1	10	0.5	1.0	9.62	13.34	0.40	3.17
log-ZQ2	10	0.5	0.1	9.63	13.23	0.39	3.17
log-ZQ2	10	0.5	1.0	9.75	12.50	0.40	3.16
identity	10	-0.5		10.00	13.34	0.37	3.14
log-ZQ1	10	-0.5	0.1	10.42	17.30	2.40	41.83
log-ZQ1	10	-0.5	1.0	10.41	16.00	0.84	4.99
log-ZQ2	10	-0.5	0.1	10.41	16.80	2.14	36.58
log-ZQ2	10	-0.5	1.0	10.31	13.99	0.61	3.97
identity	100	0.5		99.98	133.58	0.15	3.04
log-ZQ1	100	0.5	0.1	99.67	133.47	0.12	3.02
log-ZQ1	100	0.5	1.0	99.68	133.23	0.12	3.01
log-ZQ2	100	0.5	0.1	99.66	133.21	0.13	3.02
log-ZQ2	100	0.5	1.0	99.66	132.45	0.13	3.02
identity	100	-0.5		100.00	133.27	0.11	3.01
log-ZQ1	100	-0.5	0.1	100.34	134.70	0.18	3.05
log-ZQ1	100	-0.5	1.0	100.34	134.72	0.18	3.06
log-ZQ2	100	-0.5	0.1	100.34	134.68	0.18	3.07
log-ZQ2	100	-0.5	1.0	100.33	134.13	0.18	3.06

Legend: ZQ1: transformation method corresponding to equation 2.2 in Zeger and Qaqish [2]; ZQ2: transformation method corresponding to equation 2.4 in Zeger and Qaqish.

Effect of (mis)specification of the link function

The effect of choice of the link function, of the ZQ transformation and of the value of the parameter *c* on parameter estimates was studied on a simulated Poisson time series of length 1,000, with AR(p = 1) structure with $\phi_1 = 0.5$, with a logarithmic link function using transformation method ZQ1 with $c = 1$, and an intercept $\exp(\beta_0) = 2$. Models were estimated using three chains with each a length of 2,000 iterations, including a burn-in of 1,000 iterations (Table 2 in this Appendix). Convergence was assessed by studying plots of the Gelman-Rubin convergence statistic (on estimated parameters) as modified by Brooks and Gelman [3]. The computer code used, written for the R package is provided as supporting information [see Additional file S5].

The model "log-ZQ1" with c = 1 performs best, as expected. The identity link model appears to do better than the model "log-ZQ2" with $c = 1$, based on the DIC and MARE, but for the identity link model, the 95% credible interval of ϕ_1 was below 0.5, which was the value used for simulation. With $c = 0.1$, for both ZQ1 and ZQ2 transformations, the 95% credible intervals for both the intercept and ϕ_1 did not include the parameter values used for the simulation. Thus, again, the impact of the choice for constant c (in the log-link models) was strong at the (low) intercept of $\exp(\beta_0) = 2$. Thus, if a count time series has a low mean, the value of the constant should be varied in order to find the best fitting model.

Table 2 – Parameter estimates and 95% credible intervals for three types of models on a simulated Poisson AR(1) series of length 1,000 with log link function, "ZQ1" transformation, intercept = 2, $c = 1$, and $\phi_1 = 0.5$.

Link	c	intercept	ϕ_1	MARE	DIC
identity	NA	1.94 (1.81 - 2.08)	0.37 (0.32 - 0.43)	0.474	3377
log-ZQ1	0.1	2.13 (2.01 - 2.26)	0.21 (0.17 - 0.26)	0.551	3442
log-ZQ1	1.0	1.94 (1.74 - 2.13)	0.54 (0.46 - 0.61)	0.475	3355
log-ZQ2	0.1	2.13 (2.01 - 2.26)	0.21 (0.17 - 0.25)	0.551	3447
log-ZQ2	1.0	2.05 (1.91 - 2.20)	0.54 (0.46 - 0.62)	0.467	3393

Ability to estimate GSARIMA structure

Two series with GSARIMA structure were simulated, again with the R software package 'gsarima' [1], by writing the (invertible) GSARIMA model in the form of an infinite AR representation, approximated by a finite order AR representation. The model structure was then estimated using JAGS with three chains each of a length of 2000 iterations including a burn-in of 1000 iterations [see Additional file S1]. The two series were:

a) A negative binomial GSARIMA(2,1,0)×(0,0,1)$_s$ time series of length 1,000 was simulated, with a logarithmic link function, ZQ1 transformation with $c=1$, an external variable sampled $x_t \sim N(0,1)$, and $\beta_1 = 0.7$, $\phi_1 = 0.5$, $\phi_2 = 0.2$, $\theta_1^* = 0.5$, $s = 12$, $\psi = 5$.

b) A negative binomial GSARIMA(0,1,2)×(1,0,0)$_s$ time series of length 1,000 was simulated, with a logarithmic link function, ZQ1 transformation with c = 1, an external variable sampled $x_t \sim N(0,1)$, and $\beta_1 = -0.3$, $\theta_1 = 0.6$, $\theta_2 = 0.2$, $\phi_1^* = 0.4$, $s = 12$, $\psi = 3$.

The results in Table 3 in this Appendix show that the method proposed here was able to estimate the parameters correctly.

Table 3 – Parameter estimates and 95% credible intervals on simulated negative binomial GSARIMA time series of length 1,000 with log link function, ZQ1 zero transformation, an external variable sampled $x_t \sim N(0,1)$, s = 12, and c parameter 1.

Parameter	Series a		Series b	
	Target	Estimate (95% CI)	Target	Estimate (95% CI)
ψ	5	5.03 (4.49 – 5.64)	3	3.29 (2.85 – 3.77)
β_1	0.7	0.70 (0.68 – 0.71)	-0.3	-0.32 (-0.34 – -0.29)
ϕ_1	0.5	0.47 (0.41 – 0.53)		
ϕ_2	0.2	0.17 (0.11 – 0.23)		
θ_1^*	0.5	0.50 (0.46 – 0.55)		
θ_1			0.6	0.60 (0.53 – 0.66)
θ_2			0.2	0.22 (0.16 – 0.28)
ϕ_1^*			0.4	0.38 (0.32 – 0.44)

Series a) GSARIMA(2,1,0)×(0,0,1)$_s$X series with β_1=0.7, $\phi_1 = 0.5$, $\phi_2 = 0.2$, $\theta_1^* = 0.5$, and ψ = 5; Series b) GSARIMA(0,1,2)×(1,0,0)$_s$X series with β_1=-0.3, $\theta_1 = 0.6$, $\theta_2 = 0.2$, $\phi_1^* = 0.4$, and $\psi = 3$.

References

1. Briët OJT (2008) gsarima: Two functions for Generalized SARIMA time series simulation, R package version 0.0-2 [computer program]. Vienna: R Foundation for Statistical Computing.

2. Zeger SL, Qaqish B (1988) Markov regression models for time series: a quasi-likelihood approach. Biometrics 44: 1019-1031.

3. Brooks SP, Gelman A (1998) Alternative methods for monitoring convergence of iterative simulations. Journal of Computational and Graphical Statistics 7: 434-455.
